# Phylogenetic analysis and ontogenetic changes in the cone opsins of the western mosquitofish (*Gambusia affinis*)

**DOI:** 10.1371/journal.pone.0240313

**Published:** 2020-10-13

**Authors:** Chia-Hao Chang, Yu-Chun Wang, Yi Ta Shao, Shih-Hui Liu

**Affiliations:** 1 TIGP Biodiversity Program, Tunghai University, Taichung City, Taiwan; 2 Center for Ecology and Environment, Tunghai University, Taichung City, Taiwan; 3 Planning and Information Division, Fisheries Research Institute, Keelung City, Taiwan; 4 Institute of Marine Biology, National Taiwan Ocean University, Keelung, Taiwan; 5 Department of Biological Sciences, National Sun Yat-sen University, Kaohsiung City, Taiwan; University of Ferrara, ITALY

## Abstract

To convert external light into internal neural signal, vertebrates rely on a special group of proteins, the visual opsins. Four of the five types of visual opsins—short-wavelength sensitive 1 (Sws1), short-wavelength sensitive 2 (Sws2), medium-wavelength sensitive (Rh2), and long-wavelength sensitive (Lws)—are expressed in cone cells for scotopic vision, with the fifth, rhodopsin (Rh1), being expressed in rod cells for photopic vision. Fish often display differing ontogenetic cone opsin expression profiles, which may be related to dietary and/or habitat ontogenetic shift. The western mosquitofish (*Gambusia affinis*) is an aggressive invader that has successfully colonized every continent except Antarctica. The strong invasiveness of this species may be linked to its visual acuity since it can inhabit turbid waters better than other fishes. By genome screening and transcriptome analysis, we identify seven cone opsin genes in the western mosquitofish, including one *sws1*, two *sws2*, one *rh2*, and three *lws*. The predicted maximal absorbance wavelength (λ_max_) values of the respective proteins are 353 nm for Sws1, 449 nm for Sws2a, 408 nm for Sws2b, 516 nm for Rh2-1, 571 nm for Lws-1, and 519 nm for Lws-3. Retention of an intron in the *lws-r* transcript likely renders this visual opsin gene non-functional. Our real-time quantitative PCR demonstrates that adult male and female western mosquitofish do not differ in their cone opsin expression profiles, but we do reveal an ontogenetic shift in cone opsin expression. Compared to adults, larvae express proportionally more *sws1* and less *lws-1*, suggesting that the western mosquitofish is more sensitive to shorter wavelengths in the larval stage, but becomes more sensitive to longer wavelengths in adulthood.

## Introduction

Vertebrates rely on a group of specialized photoreceptor cells in the retina of eyes to convert external light into neural signals. The visual pigment located in the photoreceptor outer segment is responsible for deciphering light information and it comprises a light-absorbing protein (visual opsin) and a vitamin A-derived chromophore. Visual opsins are membrane-bound G-protein coupled receptors (GPCRs), and vitamin A-derived chromophores include vitamin A1 (retinal) or A2 (3,4-dehydroretinal). Differences in phylogenetic classes of visual opsin genes or in the types of chromophore can alter the maximal absorbance wavelength (λ_max_) of visual pigments [1]. Photoreceptor cells can be categorized as rod cells (scotopic or low-light vision) or cone cells (photopic or well-lit vision), with cone cells being further divided into single-, double-, twin-, triple-, or quadruple-cone cells [2]. Based on their expression of visual opsin genes, photoreceptor cells can also be categorized into five main classes: Rh1, Sws1, Sws2, Rh2, and Lws [3]. Most vertebrates only express one kind of rhodopsin *rh1* gene in rods [4], and the other four classes of visual opsins are all expressed in cones with different spectral sensitivities: Sws1 is sensitive to UV and violet light, Sws2 to blue, Rh2 to green, and Lws to red [1].

Habitat has a profound effect on the visual system of the animals living in it [5, 6]. For example, the cichlids in Lake Victoria have distinct *lws* alleles with different λ_max_ values that correspond to varied lighting environments with depth [7], and the opsin expression profiles of bluefin killifish (*Lucania goodei*) differ between clear springs and tannin-stained swamps [8, 9]. Apart from their lighting environments, habitats can also exert a selection pressure on visual systems in other ways. For example, predation risk variation in guppy has been demonstrated to alter *lws* allele frequencies and opsin expression profiles among populations [10].

Aquatic habitats provide more diverse lighting environments than terrestrial habitats since water itself selectively absorbs light and suspended or dissolved substances can absorb and/or scatter light [11, 12]. Multiple independent opsin gene duplication events have occurred over the evolutionary history of teleosts [13]. For example, the haplochromine cichlids have up to six cone opsins with distinct λ_max_ values, including one *sws1*, two *sws2*, two *rh2*, and one *lws* [14]. Notably even for animals with multiple cone opsins, these opsins are not expressed equally or constantly [15–17]. Lighting environments, diurnal periodicity, and diet all contribute to changes in cone opsin expression profiles [6, 15, 16]. Many fishes exhibit the ability to adjust their cone opsin expression profiles, termed “phenotypic plasticity”. One type of phenotypic plasticity is changes in expression profile as an individual grows. Such ontogenetic changes in opsin profiles have been reported for giant mottled eel (*Anguilla marmorata*), milkfish (*Chanos chanos*), barfin flounder (*Verasper moseri*), and spotted unicornfish (*Naso brevirostris*) [17–20]. Two other types of phenotypic plasticity are developmental and adult plasticity; with respect to opsins, the former pertains to how different rearing conditions can result in distinct cone opsin expression profiles [21, 22], and the latter explains how adult fish can alter their expression profiles to adapt to local lighting environments [23–25]. Both developmental and adult plasticity may enable fishes to flourish in turbid waters [22, 25].

Currently, the genomic arrangement of cone opsin genes has been fully investigated for two poeciliid fishes, i.e., guppy and green swordtail (*Xiphophorous helleri*) [26, 27]. Both of these poeciliids possess nine cone opsin genes, including one *sws1*, two *sws2*, two *rh2*, and four *lws*. The duplicated opsin paralogs are tandemly arranged, except for *lws-r*, with the two *sws2*, three *lws*, and two *rh2* opsins forming a linkage group [28]. The evolutionary trajectories of *lws* genes in poeciliid fishes reflect interactions between genomic organization and sexual selection [29].

Not all poeciliid fishes have had their cone opsin genes investigated. Although the western mosquitofish (*Gambusia affinis*) (Poeciliidae, Cyprinodotiformes) has been introduced in many countries to control mosquito populations since the early 1900s [30], becoming one of the worst invasive species in the process [31], our understanding of its cone opsins remains limited [32]. To date, research has only demonstrated that the western mosquitofish possesses all four phylogenetic classes of cone opsin genes, including two *sws2* genes (*sws2a* and *sws2b*), but exactly how many cone opsins it has remains uncertain [32]. Moreover, previous studies indicate that the western mosquitofish may display ontogenetic changes in cone opsin expression profiles because it exhibits an ontogenetic dietary shift, colors are critical to it in the mating choice of both sexes, and sex steroid estrogen influences its cone opsin expression profiles [32–36].

Sensory-based approaches can be applied to protecting endangered species and controlling alien ones [37]. A comprehensive understanding of the visual spectral characteristics of western mosquitofish could reveal how this species is so aggressively invasive and provide insights into the sensory biology of other invasive species. Here, we sought to determine how many cone opsin genes are present in the western mosquitofish and to predict the λ_max_ values of the respective opsins. Moreover, since longer-wavelength signals, i.e., yellow, are more attractive to adult individuals [34, 35], we also hypothesized that the western mosquitofish would display an ontogenetic change in cone opsin expression profiles, with the visual spectrum shifting to red during development.

## Materials and methods

Overall, a total of 84 western mosquitofish were used in this study. One adult specimen was used for transcriptome analysis, ten adult specimens were used for cloning cone opsin genes, 30 adult specimens were used for reproductive experiments, and the resulting 43 laboratory-raised specimens (comprising 30 larva, seven adult males, and six adult females) were used to quantify ontogenetic cone expression.

### Fish collection and experimental setup

Western mosquitofish samples were caught using a hand-net at two different localities, Tunghai Lake at Tunghai University (Taichung City, Taiwan) and Linyuan Ocean Wetland Park (Kaohsiung City, Taiwan). Adult fish were caught from 9:00 to 11:00 AM and then euthanized using 0.025% MS-222 (Ethyl 3-aminobenzoate, methanesulphonic acid salt). After each specimen was rendered comatose, its two eyes were removed and stored in RNA*later* solution (AM7020, Invitrogen), with the rest of the body being preserved in 100% ethanol at -20˚C until DNA extraction. Eye samples for cone opsin gene cloning and RNA-seq were first kept at 4˚C for 24 hours and then stored at -80 ˚C until RNA extraction. Overall, three males and three females from Tunghai University and two males and two females from Linyuan Ocean Wetland Park were prepared for DNA and RNA extraction.

A further 30 live adult specimens from Tunghai University were transported to the Marine Research Station of Academia Sinica (Yilan, Taiwan). These specimens were housed indoors in a glass aquarium (90 x 40 x 40 cm length:width:height) with continuous dripping influx of dechlorinated tap water to maintain water conditions as clean as possible. A full-spectrum LED grow light (Hydroponics Lin Agritech) was used as light source and placed approximately 28 cm above the water’s surface. The photoperiod of the climate-controlled room was set to 14:10 h light: dark, and the spectral irradiance of the light source was measured using a spectrometer (MK350N, Gamma Scientific) (S1 Fig). Fish were fed *ad libitum* with artificial fish feed (Otohime B2, Marubeni Nisshin Feed) twice a day. After one month, we prepared three floating fish-breeding cages in the aquarium to host pregnant females and to collect F1 offspring. When we had collected more than 50 F1 individuals, all parental individuals were removed from the aquarium and all F1 offspring were released from the cages into the main aquarium. These F1 offspring then mated to produce F2 individuals under the same lighting conditions as their parents. The floating fish-breeding cages were again employed for pregnant F1 females. The F1 adults and newborn F2 larvae (less than 10 days old) were used for ontogenetic study. In order to limit the effect of diurnal variation in opsin expression, fish were euthanized (0.025% MS-222) between 10:00 and 11:00 AM. After each specimen was rendered comatose, larval specimens were directly preserved in RNA*later* solution (AM7020, Invitrogen) whereas, for adult specimens, body weight and standard length were first determined before removing both eyes and storing them in RNA*later* solution (AM7020, Invitrogen). These samples were also kept at 4˚C for 24 hours before being transferred to -80˚C until RNA was extracted. All experiments were performed and specimens handled with approval (107–28) from the Institutional Animal Care and Use Committee (IACUC) of Tunghai University.

### DNA and RNA extraction, and cDNA preparation

DNA samples were extracted from fin tissues using a DNA extraction kit (Cat No./ID: GS100, Geneaid). An RNeasy Plus Universal Kit (Cat No./ID: 73404, QIAGEN) was used to isolate total RNA according to the manufacturer’s protocol. One eyeball from each adult specimen or five larval heads as a pooled sample was placed in a 2 ml microcentrifuge tube with stainless steel beads for tissue homogenization. Cone opsin genes are exclusively expressed in the retina, so RNA extraction from whole larval heads does not bias quantification of cone opsins [38]. The tissue was homogenized using a Roche MagNA Lyser system (Roche). Total RNA content and quality were measured using a NanoDrop 1000 (Thermo Scientific). Two micrograms (μg) of total RNA were reverse-transcribed using a Verso cDNA Synthesis Kit (Cat No. 00764129, ThermoFisher Scientific) with Random Hexamer and Anchored Oligo-dT 3:1 (v/v) in a final volume of 20 μl. The remaining RNA was preserved at -80˚C after adding RNase inhibitor (RG90925, Lucigen).

### Transcriptomics

A female specimen collected from Tunghai University was subjected to RNA sequencing (RNA-seq) by Genomics Tech. (Taipei, Taiwan). The mRNA was purified from total RNA using poly-T oligo-attached magnetic beads and then fragmented by heating. The RNA library was prepared with the TruSeq® Stranded mRNA Library Prep system (Illumina, San Diego, CA, USA). An Agilent Bioanalyzer 2100 system and a DNA High Sensitivity Chip was employed for quality control. Then, the RNA library was sequenced on an Illumina NovaSeq 6000 platform using a 150-bp paired-end strategy with an output of 6 Gb.

The raw reads were trimmed using Trimmomatic v0.36 [39] and then de-novo assembled in Trinity v2.8.4 [40]. Identical transcripts were eliminated using CD‐HIT‐EST v4.8 [41, 42], with a 95% clustering cut-off. Then, the trimmed reads were aligned onto the transcripts to estimate the sequencing depth of each transcript using Bowtie2 v2.3.4.3 [43] and RSEM v1.2.28 [44]. The average Fragments Per Kilobase Million (FPKM) was 52.67. Open reading frames (ORFs) were predicted in Transdecoder v5.3.0 (https://github.com/TransDecoder/TransDecoder) and annotated in BLASTX v2.5.0 [45]. Finally, the transcripts were translated into amino acid sequences and annotated using BLASTP v2.5.0 [45].

### Identification of cone opsin genes

Known guppy cone opsin genes––*sws1*, *sws2a*, *sws2b*, *rh2*, and *lws* (Table 1)––were used as references to query and annotate the cone opsin genes from the western mosquitofish draft genome [46] and our draft transcriptome using BLASTN 2.8.1+ [45]. We designed specific pairs of primers for each of the identified cone opsin genes. Furthermore, we aligned the complete *β*-actin gene sequence of guppy (EU143771) and the partial *β*-actin gene sequence (KP284099) of mosquitofish to design a pair of primers spanning the *β*-actin exon and intron regions. All primer sequences are presented in Table 2.

**Table 1:** List of cone opsin genes from cyprinodontiform fishes, medaka (*Oryzias latipes*), and zebrafish (*Danio rerio*).

Scientific name	*tmt-opsin*	*val-opsin*	*sws1*	*sws2*	*rh2*	*lws*
**Cypriniformes**						
*Danio rerio*	KT008411	NM_131586	BC060894	NM_131192	NM_131253	NM_001002443
			(*sws1*)	(*sws2*)	(*rh2-1*)	(*lws-1*)
					NM_182891	NM_001313715
					(*rh2-2*)	(*lws-2*)
					NM_182892	
					(*rh2-3*)	
					NM_131254	
					(*rh2-4*)	
**Beloniformes**						
*Oryzias latipes*			AB223058	AB223056	AB223053	AB223051
			(*sws1*)	(*sws2a*)	(*rh2-a*)	(*lws-a*)
				AB223057	AB223054	AB223052
				(*sws2b*)	(*rh2-b*)	(*lws-b*)
					AB223055	
					(*rh2-c*)	
**Cyprinodontiformes**						
**Poeciliidae**						
*Poecilia reticulata*			HQ260685	HQ260684	HQ391990	HQ260679
			(*sws1*)	(*sws2a*)	(*rh2-1*)	(*lws-1*)
				HQ391991	HQ260683	HQ260680
				(*sws2b*)	(*rh2-2*)	(*lws-2*)
						HQ260681
						(*lws-3*)
						HQ260682
						(*lws-r*)
*Poecilia caymanensis*			KX768650	KX768635	KX768663	KX768609
			(*sws1*)	(*sws2a*)	(*rh2-1*)	(*lws-1*)
				KX768622		KX768598
				(*sws2b*)		(*lws-2*)
						KX768587
						(*lws-3*)
						KX768576
						(*lws-r*)
*Poecilia velifera*			KX768639	KX768624	KX768654	KX768600
			(*sws1*)	(*sws2a*)	(*rh2-1*)	(*lws-1*)
				KX768611		KX768589
				(*sws2b*)		(*lws-2*)
						KX768578
						(*lws-3*)
						KX768567
						(*lws-r*)
*Limia nigrofasciata*			KX768649	KX768634	KX768662	KX768608
			(*sws1*)	(*sws2a*)	(*rh2-1*)	(*lws-1*)
				KX768621		KX768597
				(*sws2b*)		(*lws-2*)
						KX768586
						(*lws-3*)
						KX768575
						(*lws-r*)
*Heterandria formosa*			KX768651	KX768636	KX768664	KX768610
			(*sws1*)	(*sws2a*)	(*rh2-1*)	(*lws-1*)
				KX768623		KX768599
				(*sws2b*)		(*lws-2*)
						KX768588
						(*lws-3*)
						KX768577
						(*lws-r*)
*Micropoecilia bifurca*			KX768646	KX768631	KX768659	KX768605
			(*sws1*)	(*sws2a*)	(*rh2-1*)	(*lws-1*)
				KX768618		KX768594
				(*sws2b*)		(*lws-2*)
						KX768583
						(*lws-3*)
						KX768572
						(*lws-r*)
*Pamphorichthys minor*			KX768647	KX768632	KX768660	KX768606
			(*sws1*)	(*sws2a*)	(*rh2-1*)	(*lws-1*)
				KX768619		KX768595
				(*sws2b*)		(*lws-2*)
						KX768584
						(*lws-3*)
						KX768573
						(*lws-r*)
*Xiphophorus hellerii*			GU454734		GU454732	DQ075246
			(*sws1*)		(*rh2-1*)	(*lws-1*)
					GU454733	
					(*rh2-2*)	
*Gambusia affinis*			MN817658	MN817660	MN817659	MN817662
			(*sws1*)	(*sws2a*)	(*rh2-1*)	(*lws-1*)
				MN817661		MN817663 (*lws-3*)
				(*sws2b*)		
						Hoffberg, Troendle [46]
						(*lws-r*)

Two non-visual opsins—*tmt-opsin* and *val-opsin*—from zebrafish are used as outgroups for cone opsins.

**Table 2:** Sequences of the primers used for amplifying *β*-actin and cone opsin genes and for quantifying expression levels of cone opsin genes in the western mosquitofish.

Gene	Primer sequence (5'-3')	Length of Complete cDNA (bp)	Length of target cDNA segment (bp)	Reference
Gene amplification				
*sws1*	For: TGAGGTCGGAGGAACTCA	1008	1068	this study
	Rev: TGGATCCTCTGAACTCAGATC			
*sws2a*	For: AATTCAACCGAGTCGTAGAC	1056	1061	this study
	Rev: GTTTGGACATAGCATTTACGA			
*sws2b*	For: TATACCGCATCCAGCTCGTA	1065	1143	this study
	Rev: ACACGTCAATGTTGGTCATTC			
*rh2-1*	For: CAGTGAAATCTCCCACTCAC	1059	1081	this study
	Rev: CAGACTTGAAGACGCGATTA			
*lws-1*	For: GAGGAAGGTCTGAGAGCTGA	1071	1040	this study
	Rev: TGATGTGGACACTTCAGAGC			
*lws-3*	For: GCCGAGAGAAGATCTCCAGA	1071	1040	this study
	Rev: TGATGTGGACACTTCAGAGC			
*lws-r*	For: ATCAAGCAGCTCAGATCGTC	*	1310	this study
	Rev: TGATGTGGACACTTCAGAGC			
*β-*actin	For: CCTGTACGCTTCTGGTCGTA	1128	566 (776^a^)	this study
	Rev: CCTCCAATCCAGACAGAGTA			
qPCR				
*sws1* (*Ei* = 96.65%)	For: CCGACTGGTACACCCACAAT		56	Friesen et al. 2017 [32]
	Rev: TGGACAGAGGCATGCAGAAG			
*sws2a* (*Ei* = 100.05%)	For: TCTGCTTCTGCTTTGCCGTA		92	Friesen et al. 2017 [32]
	Rev: TGGTCACCTCCTTTTCAGCC			
*sws2b* (*Ei* = 100.04%)	For: TTATCTGCAAGCCACTCGGG		104	Friesen et al. 2017 [32]
	Rev: AACACTGCATTCCCTCAGGG			
*rh2-1* (*Ei* = 96.45%)	For: TCAGAACAAAAAGCTGCGCC		46	Friesen et al. 2017 [32]
	Rev: TGAAACCAAAGGCGCACATG			
*lws-1* (*Ei* = 97.95%)	For: CTGAGAGCTGAGAAACCTTC		54	this study
	Rev: CTCTTGTTGTATCTTCGTGC			
*lws-3* (*Ei* = 97.10%)	For: AGAGAAGTGTGAGGTGAAGC		112	this study
	Rev: CTCTTGTTGTATCTTCGTGC			

*Ei* = efficiency of qPCR primers.

*lws-r is likely a pseudogene.

^a^Length of target DNA segment.

PCR amplifications of opsin genes were performed in a final reaction volume of 25 μL, containing 2 ng cDNA, 1 μl each of the forward and reverse primers (10 μM), 12.5 μl of Fast-Run^TM^ Advanced Taq Master Mix (ProTech, Taipei, Taiwan), and distilled water. The thermal cycling protocol was as follows: one cycle at 94°C for 4 min; 35 cycles of denaturation at 94°C for 30 sec, 55–60°C for 30 sec, and 72°C for 1 min; and a final single extension step at 72°C for 5 min. Sequencing was performed by Mission Biotech Inc., Taipei, Taiwan. The contig sequences were constructed using the program CodonCode Aligner 6.0.2, and the results were identified by BLAST analysis against the database of the National Center for Biotechnology Information (NCBI).

### Phylogenetic analyses

The cone opsin gene sequences of other poeciliid fishes, medaka (*Oryzias latipes*), and zebrafish (*Danio rerio*) were downloaded from NCBI, and two non-visual opsin genes of the zebrafish—*val-opsin* and *tmt-opsin*—were selected as outgroups [38]. All opsin gene sequences included in our phylogenetic analysis are shown in Table 1. Gene sequences were aligned using MACSE v2, which is designed to align protein-coding nucleotide sequences based on their corresponding amino acid translations [47]. Phylogenetic analyses were performed using a partitioned Maximum Likelihood (ML) approach. RAxML 8 [48] was used for ML analyses (MLA). Partitions were set with respect to codon position, and the GTR+G+I model (with four discrete rate categories) was adopted for each partition [49]. The ML tree was obtained by performing 100 different runs using the default algorithm. The best ML tree was chosen from likelihood scores among suboptimal trees from each run. Nodal support for MLA was determined from bootstrap analysis using RAxML [50], employing 10,000 non-parametric bootstrap replications with the ML criterion.

Apart from targeting the coding region of opsins, we also conducted phylogenetic analysis on the 5’ untranslated region (UTR) of *lws* from many poeciliid fishes given that Watson et al. (2011) [26] and Sandkam et al. (2017) [29] have demonstrated that gene conversion between poeciliid *lws* paralogs is more frequent in coding regions than in non-coding regions. Upon adding our 5’ UTR data from western mosquitofish to the poeciliid data of Sandkam et al. (2017) [29], the sequences were aligned using MUSCEL in Seqotron [51]. MLA was performed with the GTR+G+I model (with four discrete rate categories) [49] in RAxML 8 [48]. The ML tree was obtained by performing 100 different runs using the default algorithm. The best ML tree was chosen from likelihood scores among suboptimal trees from each run. Nodal support for MLA was determined from bootstrap analysis with RAxML [50], employing 10,000 non-parametric bootstrap replications with the ML criterion.

### Prediction of the λ_max_ values of the cone opsins

To predict the λ_max_ values of western mosquitofish cone opsins, we relied on inferences based on its closest evolutionary relative, the guppy, for which λ_max_ values were determined previously from *in vitro* expression data [52]. Moreover, given that some amino acid residues of a visual opsin have been demonstrated to have more pronounced effects on the λ_max_ value than other residues, for this study, not only did we target the five residues (the 180^th^, 197^th^, 277^th^, 285^th^, and 308^th^ amino acid residues corresponding to the human M/LWS pigment) based on the “5-sites rule” [1, 53], but also an additional five residues (the 110^th^, 112^th^, 138^th^, 223^th^, and 227^th^ amino acid residues corresponding to the human M/LWS pigment), which also influence the λ_max_ value of Sws2a in barfin flounder (*Verasper moseri*) and Rh-2 opsins in *Oryzia* [20, 54]. We assumed that the λ_max_ values of two opsins were identical when a western mosquitofish cone opsin presented the same ‘10 key-sites’ composition as the orthologous opsin in guppy.

### Real-time quantitative PCR (qPCR)

The specific primers designed for qPCR were based on the annotated sequences from our whole-genome BLAST and our draft transcriptome. The amplification efficiency and melting curve of each qPCR primer pair was tested by 5-fold serial dilutions of the templates, with three replicates for each gene and sample. Each qPCR primer pair was adopted only when its amplification efficiency fell between 90% and 110% and the melting curve analysis revealed each pair of specific primers only generated a single product. Expression of opsin genes was determined by qPCR in a Roche LightCycler480 system (Roche). Each reaction contained 10 μl of Roche LightCycler480 SYBR Green I Master (Roche Applied Science, Indianapolis, IN), 50 ng of cDNA, and 1 μl of each primer (10 μM) (Table 2) in a final volume of 20 μl. The qPCR reactions were performed in a LightCycler 480 Multiwell Plate system (Roche 04729692001) with Optical Adhesive Film (Applied Biosystems Ref 4360954). The following thermal cycles were performed: one cycle of 50°C for 2 min and 95°C for 10 min; followed by 45 cycles of 95°C for 10 sec, 60°C for 10 sec, and 72°C for 10 sec; and then one cycle of 95°C for 5 sec and 65°C for 1 min. qPCR products were qualified according to a melting-curve analysis. Additionally, representative samples were electrophoresed to verify that only a single product (band) was present. RNA-free water was used as a template in the control reactions to determine non-specific primer amplification background levels. Three replicates were performed for each cone opsin gene for each specimen.

Proportional expression of each cone opsin was calculated using the following equation:

$$\frac{T_{i}}{T_{all}}=\frac{\left(1/{\left(1+E_{i}\right)}^{C_{ti}}\right)}{\sum (1/{\left(1+E_{i}\right)}^{C_{ti}})}$$

where *T*_*i*_*/T*_*all*_ is the proportional expression of a given opsin gene *i*, and *E*_*i*_ is the amplification efficiency for each pair of opsin primers.

Expression of each cone opsin was first evaluated using Levene’s test and then compared using one-way ANOVA with a *post-hoc* Tukey HSD when Levene’s test was not significant or using Welch’s ANOVA with a Games-Howell *post-hoc* test when Levene’s test was significant. Statistical tests were performed in R version 3.6.0 (R Foundation for Statistical Computing, Vienna, Austria).

## Results

### Identification of cone opsins

The naming of poeciliid cone opsins has varied through time, but was recently reviewed by Sandkam et al. (2018) [28]. Based on that review, we have labelled the cone opsins we identified in western mosquitofish according to the orthologous opsins in guppy. We identified five cone opsin genes from the published draft genome of western mosquitofish [46]: *sws1*, *sws2a*, *sws2b*, *rh2-1*, and *lws-r*. We annotated six cone opsins from our draft transcriptome, including *sws1*, *sws2a*, *sws2b*, *rh2-1*, *lws-1*, and *lws-3*. These newly identified cone opsin genes (apart from *lws-r*, see below) were amplified from cDNA using specific primers (Table 2), with accession numbers MN817658 to MN817663 (Table 1).

**Fig 1:**
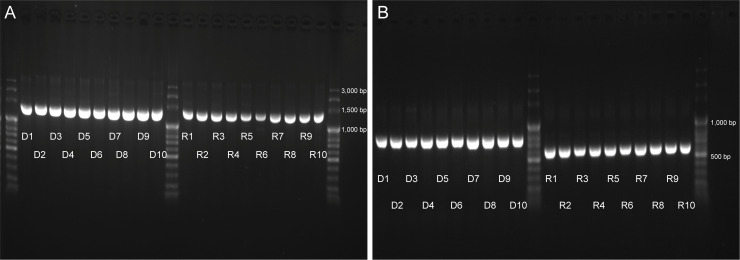
PCR-amplified fragment sizes of *lws-r* and *β*-actin. (A) *lws-r* opsin, and (B) *β*-actin. The initial character of each lane label represents DNA (D) or cDNA (R), and the number represents a specific specimen: 1 to 3 are female specimens collected at Tunghai University; 4 to 6 are male specimens collected from Tunghai University; 7 and 8 are female specimens collected at Linyuan Ocean Wetland Park; and 9 and 10 are male specimens collected at Linyuan Ocean Wetland Park.

The size of *β*-actin fragments amplified using DNA or cDNA as template differed (Fig 1B), with DNA templates producing larger fragments. However, amplified *lws-r* fragments were all identical in size no matter which template was employed (Fig 1A). Compared to the cDNA sequence of *lws-r* in guppy (KX768568), the western mosquitofish *lws-r* transcript contains an intron and also has a 46-basepair deletion in the coding region (equivalent to positions 1166 to 1211 of the guppy complete *lws-r* sequence). This intronic retention in the western mosquitofish *lws-r* transcript would result in an early stop codon during translation (S2 Fig), so it was excluded from further analyses apart from our phylogenetic assessment of the 5’ UTR region of *lws*.

### Phylogenies of western mosquitofish cone opsins

**Fig 2:**
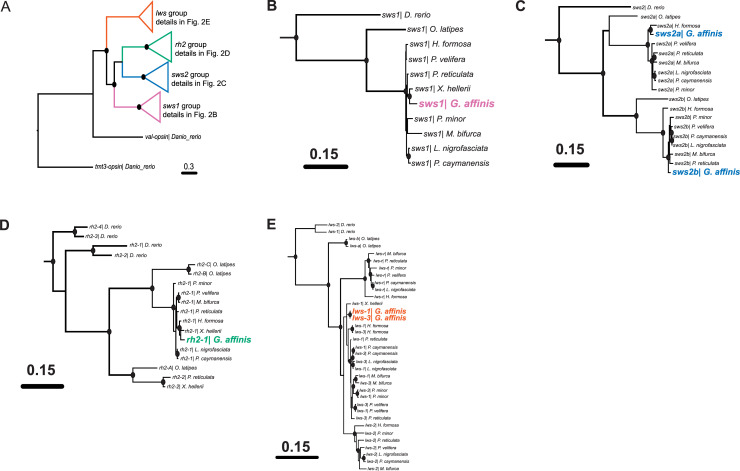
Phylogenetic tree of the coding regions from cone opsin genes based on partitioned maximum likelihood analysis (three partitions). Branch colors represent different cone opsin families. Phylogenies for each opsin family are shown in panels B to E. Solid circles on branch nodes indicate statistically robust nodes with bootstrap values ≥ 70.

The cone opsin gene dataset comprised a total of 85 taxa and 1344 aligned nucleotide positions, with 983 variable sites and 848 parsimony-informative sites (S1 Appendix). In our cone opsin ML tree, the *sws1*, *sws2*, *rh2*, and *lws* families are all respectively clustered with high statistical support (bootstrap values ≥ 70), with the *lws* family lying basal to these other three cone opsin families (Fig 2). This cone opsin ML tree also demonstrates that all six cone opsins from the western mosquitofish cluster with their corresponding cone opsin genes from other poeciliid fishes with high statistical support. The two western mosquitofish *lws* genes (*lws-1* and *lws-3*) are clustered together and are paralogous to the corresponding genes of other poeciliid fishes (Fig 2E), and the other four cone opsin genes of the western mosquitofish (*sws1*, *sws2a*, *sws2b*, and *rh2-1*) are all orthologous to their corresponding genes in other poeciliids (Fig 2B to 2D).

**Fig 3:**
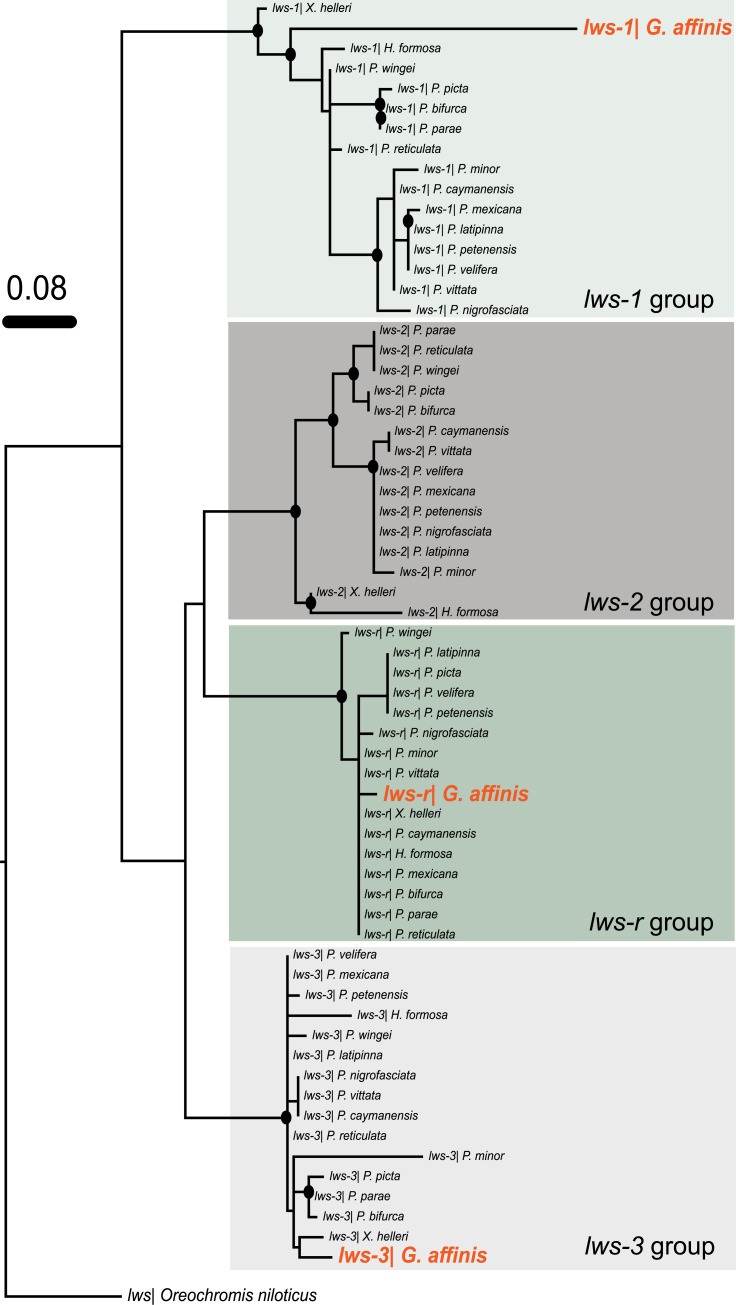
Phylogenetic tree of the 5’ untranslated region (UTR) of poeciliid *lws* opsin genes based on maximum likelihood analysis. *Oreochromis niloticus* was used as outgroup. Solid circles on branch nodes indicate statistically robust nodes with bootstrap values ≥ 70.

The 5’ UTR dataset of *lws* genes comprised a total of 64 taxa and 140 aligned nucleotide positions (S2 Appendix), with 111 variable sites and 84 parsimony-informative sites. From our *lws* 5’ UTR ML tree (Fig 3), it is clear that *lws* genes from poeciliid fishes can be divided into four strongly supported groups: *lws-1* group, *lws-2* group, *lws-3* group, and *lws-r* group. The three *lws* genes possessed by western mosquitofish (*lws-1*, *lws-3*, and *lws-r*) are all orthologous to the corresponding *lws* genes of other poeciliid fishes. Notably, our genomic (BLAST) and transcriptomic analyses reveal *lws-2* to be absent from western mosquitofish.

### Predicted λ_max_ values of western mosquitofish cone opsins

The λ_max_ values of guppy cone opsins are 353 nm (Sws1), 438 nm (Sws2a), 408 nm (Sws2b), 516 nm (Rh2-1), 571 nm (Lws-1), and 519 nm (Lws-3) [52]. Apart from Sws2a, all western mosquitofish cone opsins have the same ‘10-sites’ composition as their orthologs in guppy, so we infer the λ_max_ values of western mosquitofish cone opsins to be as follows: Sws1 = 353 nm, Sws2b = 408 nm, Rh2-1 = 516 nm, Lws-1 = 571 nm, and Lws-3 = 519 nm. Compared to western mosquitofish Sws2a, guppy Sws2a has an Ala-to-Thr mutation at residue 110 (_Ala_110_Thr_) and a Thr-to-Ala mutation at residue 285 (_Thr_285_Ala_). Comparing the two orthologous Sws2a opsins from guppy and medaka, we assume that the effect of _Ala_110_Thr_ on the λ_max_ value is -1 nm. Moreover, Cowing et al. (2002) [55] inserted the _Thr_285_Ala_ mutation into the Sws2 opsin of *Cottus gobio* and observed an approximatley -10 nm shift in the λ_max_ value. Accordingly, we infer a λ_max_ value for western mosquitofish Sws2a of 449 (= 438+1+10) nm (Table 3).

**Table 3:** Ten-site amino acid haplotypes for cone opsin genes in guppy and western mosquitofish.

Scientific name	Opsin	Key site position										Measured λmax (nm)	Predicted λmax (nm)
		110	112	138	180	197	223	227	277	285	308		
*Oryzias latipes*	Lws-A	S	C	I	S	H	L	C	Y	T	A	561^a^	
	Lws-B	S	C	I	S	H	L	C	Y	T	A	562^a^	
*Poecilia reticulata*	Lws-1	S	C	I	A/ S	H	L	C	Y	T	A	A: 562^b^/ S: 571^b^	
	Lws-2	S	C	I	P	H	L	C	F	A	A	516^b^	
	Lws-3	S	C	I	S	H	L	C	Y	T	A	519^b^	
	Lws-r	S	C	I	S	H	L	C	Y	T	A		
*Gambusia affinis*	Lws-1	S	C	I	S	H	L	C	Y	T	A		571
	Lws-3	S	C	I	S	H	L	C	Y	T	A		519
*Oryzias latipes*	Rh2-A	C	Y	Q	A	E	M	H	F	A	S	452^a^	
	Rh2-B	T	T	E	A	E	M	H	F	A	A	516^a^	
	Rh2-C	T	N	Q	A	E	M	H	F	A	A	492^a^	
*Poecilia reticulata*	Rh2-1	T	T	E	A	E	M	H	F	A	A	516^b^	
	Rh2-2	T	Y	Q	A	E	L	H	F	A	A	476^b^	
*Gambusia affinis*	Rh2-1	T	T	E	A	E	M	H	F	A	A		516
*Oryzias latipes*	Sws2a	A	C	M	A	E	L	C	F	A	S	439^a^	
*Poecilia reticulata*	Sws2a	T	F	M	A	E	L	C	F	A	S	438^b^	
*Gambusia affinis*	Sws2a	A	C	M	A	E	L	C	F	T	S		449^c^
*Oryzias latipes*	Sws2b	C	V	M	G	E	L	C	F	A	S	405^a^	
*Poecilia reticulata*	Sws2b	C	Y	M	A	E	L	C	F	A	S	408^b^	
*Gambusia affinis*	Sws2b	C	Y	M	A	E	L	C	F	A	S		408
*Oryzias latipes*	Sws1	V	L	L	G	E	L	C	F	A	A	356^a^	
*Poecilia reticulata*	Sws1	V	V	L	G	E	L	C	F	A	A	353^b^	
*Gambusia affinis*	Sws1	V	V	L	G	E	L	C	F	A	A		353

Measured λ_max_ of guppy refers to reconstituted opsins. Predicted λ_max_ of western mosquitofish refers to predictions based on the orthologous opsin in guppy.

^a^λ_max_ value from Matsumoto et al. (2006) [56]

^b^λ_max_ value from Kawamura et al. (2016) [52].

^c^Inferred from the λ_max_ value of the orthologous opsin in guppy and the putative -10 nm effect of the Thr-to-Ala mutation at residue 285 [55] and the putative -1 nm effect of the Ala-to-Thr mutation at residue 110.

### Cone opsin gene expression

**Fig 4:**
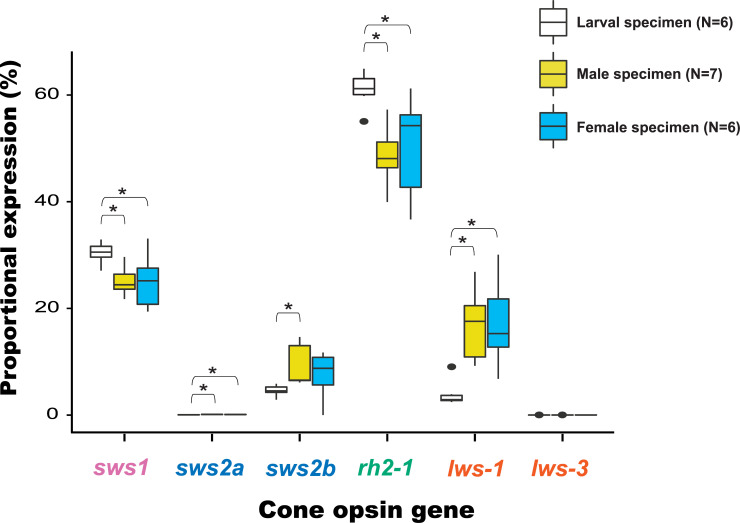
Box-plot showing the median (—), 25^th^ and 75^th^ percentiles (box), 95% range (|), and outliers (•) of cone opsin proportional expression values for adult and larval specimens. An asterisk (*) indicates p < 0.05.

The *rh2-1* cone opsin gene exhibited the greatest expression in all developmental stages we tested and for both sexes, whereas proportional expression levels of both the *sws2a* and *lws-3* opsin genes were always lower than 1% (Fig 4). Apart from *lws-3* (ANOVA *F*_2, 16_ = 1.3571, p > 0.05), proportional expression of all of the western mosquitofish cone opsin genes was significantly different among larval, adult male, and adult female specimens (Fig 4). One-way ANOVA and *post-hoc* Tukey HSD revealed that larvae expressed more Sws1 and Sws2a opsins than adults of both sexes (Sws1 *F*_2, 16_ = 4.9853, p < 0.05; Sws2a *F*_2, 16_ = 13.529, p < 0.05). A Welch’s ANOVA test also supported that expression levels of the *sws2b* (*F*_2, 9.108_ = 6.0534, p < 0.05), *rh2-1* (*F*_2, 10.672_ = 13.231, p < 0.05), and *lws-1* (*F*_2, 9.81_ = 16.72, p < 0.05) genes were all significantly different among larval, adult male, and adult female specimens. A post-hoc Games-Howell test revealed that larvae expressed less Sws2b opsin than adult males, less Lws-1 than adults of both sexes, but more Rh2-1 opsin than adults of both sexes.

## Discussion

We identified a total of seven cone opsin genes, including one *sws1*, two *sws2*, one *rh2*, and potentially three *lws*, in this study. In teleosts, a visual opsin genic tandem array is commonly observed for *sws2*, *rh2*, and *lws* paralogs [13], and the *sws2-lws* synteny is a synapomorphy from the common ancestor of mammals, birds, reptiles, and fish [57]. However, the western mosquitofish draft genome [46] only revealed that two *sws2* genes are tandemly arrayed, indicating that the draft genome may not be complete. Lin et al. (2017) [13] considered one of the *lws* genes, *lws-r*, to be apomorphic in the Cyprinodontoidei since it lacks intron II-V but has a highly conserved exon/intron I. The *lws-r* gene is also thought to have arisen by retrotransposition and is located in an intron of *gephyrin* (*gphn*) [26, 58]. Here, we confirm by whole-genome BLAST analysis that the western mosquitofish has the *lws-r* gene and, as for guppy and green swordtail, its *lws-r* also contains only one intron and is located in a *gphn* gene intron.

We identified a further two western mosquitofish *lws* opsin transcripts (*lws-1* and *lws-3*) by RNA-seq. In our phylogenetic tree of *lws* 5’ UTRs, these two *lws* genes cluster separately and with high statistical support with other poeciliid *lws-1* and *lws-3* genes, respectively (Fig 3). However, the phylogenetic analysis based on the coding regions revealed a very different topology in that these two *lws* genes grouped together with high statistical support rather than separately clustering with their own orthologous *lws* genes (Fig 2E). Thus, the *lws-1* and *lws-3* genes appear to group by species as if independent gene duplication events have occurred in poeciliid fishes. Sandkam et al. (2017) [29] stated that the genomic environment in poeciliid fishes tends to homogenize their *lws-1* and *lws-3* genes through gene conversion because of their genomic proximity, gene structure, and orientation, but that this gene conversion may be curbed in some lineages where bright red/orange male coloration is critical to sexual selection. Moreover, the distinct topologies of the coding region-based tree and the 5’ UTR-based tree is consistent with gene conversion being more frequent in coding regions than in non-coding ones [62]. Although the draft western mosquitofish genome does not reveal if *lws-1* and *lws-3* are in close proximity, have a similar gene structure, or are in inverted orientations, our coding region-based phylogenetic tree may indicate that gene conversion has homogenized the western mosquitofish *lws-1* and *lws-3* genes.

Compared to the guppy (nine cone opsins), western mosquitofish appear to lack two cone opsin genes, i.e., *rh2-2* and *lws-2*. It is possible that these two opsins have been lost from the western mosquitofish genome, which is not unusual among the Cyprinodontoidei [13]. Alternatively, these two opsins do exist in the western mosquitofish genome but they are not (or barely) expressed in the larval/adult stages we sampled.

That the *lws-r* gene is located in the *gphn* intronic region raises two questions. First, does *lws-r* interfere with the expression of *gphn* and, second, is a hybrid transcript generated [27]? Based on our draft transcriptome, we can confirm that the western mosquitofish expresses *gphn* as well as the green swordtail, and can also rule out gene fusion between these two genes. However, although western mosquitofish *lws-r* is present in the draft genome and we successfully amplified it from cDNA, it is absent from our transcriptome assembly. Interestingly, the *lws-r* sequences from DNA and cDNA template (Fig 1A) were of the same length and both contained the intron region (S2 Fig). This outcome is not likely due to DNA contamination because *β*-actin fragments amplified from cDNA samples exhibited a single size and were shorter than those of DNA samples (Fig 1B).

In the process of library preparation for RNA-seq, we used poly-T oligo-attached magnetic beads to purify mRNA. Thus, the absence of *lws-r* in our RNA-seq dataset may be attributable to *lws-r* transcript not undergoing full post-transcriptional modification so that the poly(A) tail had not been added. Alternatively, transcription of the *gphn* gene may enable *lws-r* in guppy and green swordtail to manifest [27, 58]. This “hijack” hypothesis may explain why the premature *lws-r*, containing the intronic sequence and lacking the poly(A) tail, could be amplified from cDNA template. In our study, cDNA samples were reverse-transcribed using both Hexamer and Anchored Oligo-dT, so the spliced intronic fragments from post-transcriptional *gphn* modification could be converted into cDNA. It should be noted that we did not detect any “mature” mRNA of *lws-r* from either larval or adult samples, so it is doubtful that Lws-r exerts a light-sensing function in western mosquitofish. Yokoyama et al. (2014) [59] reported that the *sws1* opsin gene of the pearleye (*Benthalbella dentata*) became a pseudogene about 130 million years ago, but it still produces transcripts and has a low nucleotide substitution rate, perhaps indicating that it retains some unknown functions. It would be interesting to determine if *lws-r* in the western mosquitofish is a functional visual opsin, has other unknown functions, or its transcript is just a side product of *gphn* expression.

Apart from Sws2a, we found that the other five identified cone opsins in western mosquitofish possess the same “10-sites” composition as their guppy orthologues. Accordingly, we inferred that these five cone opsins have the same λ_max_ values as defined for guppy. A shift in the λ_max_ value of a visual opsin can alter the evolutionary trajectory of another one. For instance, in many species of flounder, a shift to green of Sws2a has been suggested as having compensated for the functional loss of one of their Rh2 paralogs [20, 60]. This green-shift phenomenon might also occur in western mosquitofish. Compared to guppy Sws2a, the _Ala_110_Thr_ and _Thr_285_Ala_ mutations in western mosquitofish Sws2a may induce an 11-nm shift in long-wavelength [55, 56]. By incorporating more closely-related poeciliids in further genomic studies, it should be possible to determine if the greenward shift of Sws2a is a response to loss of the *rh2-2* gene from the western mosquitofish genome, as has been reported for *Verasper* fishes [60].

Our qPCR results reveal that apart from *lws-3* with less than 0.05% proportional expression for all developmental stages and sexes, the remaining five cone opsins all display significant differences in proportional expression between larval and adult stages. The significant ontogenetic decrease in *sws1* expression and increase in *lws-1* suggest that the visual spectrum of adult western mosquitofish is more sensitive to longer wavelengths than that of larvae. UV sensitivity is critical for detecting zooplankton [61–66]. Many fishes display an ontogenetic long-wavelength shift in spectral sensitivity when they no longer feed on zooplankton [67–70]. Since *Gambusia* fishes exhibit an ontogenetic dietary shift from micro to large invertebrates [33, 71], the decline in ultraviolet sensitivity of western mosquitofish is consistent with its ontogenetic dietary shift.

The *lws-1* allele of western mosquitofish is 180S, which is a more red-sensitive allele (180S, λ_max_ = 571 nm, compared to 180A λ_max_ = 562 nm; Table 3). Sandkam et al. (2015) [10] demonstrated that female guppy carrying the 180S allele prefer males with more abundant red/orange coloration than those carrying the 180A allele. Therefore, allele type and expression levels of *lws-1* could not only explain why adult male western mosquitofish are strongly attracted to the yellow spot around the urogenital opening of mature females [34], but also why female western mosquitofish are more inclined to breed with male guppies than conspecific males [72].

In conclusion, we have verified that the western mosquitofish has seven cone opsin genes, including one *sws1*, two *sws2*, one *rh2*, and three *lws*, but whether the *lws-r* gene is functional or not remains unclear. The predicted λ_max_ value for Sws1 is 353 nm, it is 449 nm for Sws2a, 408 nm for Sws2b, 526 nm for Rh2-1, 571 nm for Lws-1, and 519 nm for Lws-3. The mutations at residues 110 and 285 of Sws2a have likely induced a green-shift in the λ_max_ value of that opsin, which may be associated with loss of the *rh2-2* gene. We have also revealed an ontogenetic red shift in the visual spectrum of western mosquitofish. The ontogenetic decline in UV-sensitivity could be correlated with a dietary shift. Furthermore, *lws-1* allele type and expression level appears to reflect the asymmetric negative reproductive interference reported between sympatric guppy and western mosquitofish. The comprehensive understanding of cone opsins in western mosquitofish from this study will facilitate further explorations of the mechanisms, such as developmental and/or adult plasticity of opsin expression, that enable western mosquitofish to flourish better in turbid waters compared to other fishes [73].

## Supporting information

S1 Fig The lighting spectrum of the full-spectrum LED light deployed above the aquarium in this study. (JPG)

S2 Fig Nucleotide sequence alignment of the western mosquitofish *lws-r* fragment from the published draft genome [51], the sequences we generated from DNA and cDNA templates in this study, and the published *lws-r* complete gene and cDNA sequences of guppy (KX768568). (PDF)

S1 Appendix (DOCX)

S2 Appendix (DOCX)

S1 Original image (JPG)

S2 Original image (JPG)

## References

[1] Yokoyama S. Molecular evolution of vertebrate visual pigments. Prog Retin Eye Res. 2000;19(4):385–491. 10.1016/s1350-9462(00)00002-1 10785616

[2] Locket NA. Vertebrate photoreceptors Archer SN, Djamgoz MBA, Loew ER, Partridge JC, Vallerga S, editors. Dordrecht: Springer; 1999.

[3] Chang BSW, Crandall KA, Carulli JP, Hartl DL. Opsin phylogeny and evolution: a model for blue shifts in wavelength regulation. Mol Phylogenet Evol. 1995;4(1):31–43. 10.1006/mpev.1995.1004 7620634

[4] Musilova Z, Cortesi F, Matschiner M, Davies WIL, Patel JS, Stieb SM, et al Vision using multiple distinct rod opsins in deep-sea fishes. Science. 2019;364(6440):588–92. 10.1126/science.aav4632 31073066 PMC6628886

[5] Lythgoe JN. Ecology of Vision. New York: Oxford University Press; 1979.

[6] Hauser FE, Chang BS. Insights into visual pigment adaptation and diversity from model ecological and evolutionary systems. Curr Opin Genet Dev. 2017;47:110–20. 10.1016/j.gde.2017.09.005 29102895

[7] Seehausen O, Terai Y, Magalhaes IS, Carleton KL, Mrosso HDJ, Miyagi R, et al Speciation through sensory drive in cichlid fish. Nature. 2008;455:620–6. 10.1038/nature07285 18833272

[8] Fuller RC, Fleishman LJ, Leal M, Travis J, Loew E. Intraspecific variation in retinal cone distribution in the bluefin killifish, *Lucania goodei*. J Comp Physiol A. 2003;189(8):609–16. 10.1007/s00359-003-0435-x 12879350

[9] Fuller RC, Carleton KL, Fadool JM, Spady TC, Travis J. Population variation in opsin expression in the bluefin killifish, *Lucania goodei*: a real-time PCR study. J Comp Physiol A. 2004;190(2):147–54. 10.1007/s00359-003-0478-z 14685760

[10] Sandkam B, Young CM, Breden F. Beauty in the eyes of the beholders: colour vision is tuned to mate preference in the Trinidadian guppy (*Poecilia reticulata*). Mol Ecol. 2015;24(3):596–609. 10.1111/mec.13058 25556876

[11] Kirk JTO. Use of a quanta meter to measure attenuation and underwater reflectance of photosynthetically active radiation in some inland and coastal south-eastern Australian waters. Australian J Mar Freshwater Res. 1977;28(1):9–21. 10.1071/MF9770009

[12] Kirk JTO. Attenuation of light in natural waters. Australian J Mar Freshwater Res. 1977;28(4):497–508. 10.1071/MF9770497

[13] Lin J-J, Wang F-Y, Li W-H, Wang T-Y. The rises and falls of opsin genes in 59 ray-finned fish genomes and their implications for environmental adaptation. Sci Rep. 2017;7:15568 10.1038/s41598-017-15868-7 29138475 PMC5686071

[14] Carleton K. Cichlid fish visual systems: mechanisms of spectral tuning. Integr Zool. 2009;4(1):75–86. 10.1111/j.1749-4877.2008.00137.x 21392278

[15] Yourick MR, Sandkam BA, Gammerdinger WJ, Escobar‐Camacho D, Nandamuri SP, Clark FE, et al Diurnal variation in opsin expression and common housekeeping genes necessitates comprehensive normalization methods for quantitative real‐time PCR analyses. Mol Ecol Resour. 2019;19(6):1447–60. 10.1111/1755-0998.13062 31325910 PMC6995727

[16] Sandkam BA, Deere-Machemer KA, Johnson AM, Grether GF, Rodd FH, Fuller RC. Exploring visual plasticity: dietary carotenoids can change color vision in guppies (*Poecilia reticulata*). J Comp Physiol A. 2016;202:527–34. 10.1007/s00359-016-1097-9 27283858

[17] Tettamanti V, de Busserolles F, Lecchini D, Marshall NJ, Cortesi F. Visual system development of the spotted unicornfish, *Naso brevirostris* (Acanthuridae). J Exp Biol. 2019;222:jeb209916 10.1242/jeb.209916 31776185

[18] Wang F-Y, Fu W-C, Wang I-L, Yan HY, Wang T-Y. The giant mottled eel, *Anguilla marmorata*, uses blue-shifted rod photoreceptors during upstream migration. PLoS One. 2014;9(8):e103953 10.1371/journal.pone.0103953 25101636 PMC4125165

[19] Chang C-H, Chiao C-C, Yan HY. Ontogenetic changes in color vision in the milkfish (*Chanos chanos* Forsskål, 1775). Zool Sci. 2009;26(5):349–55. 10.2108/zsj.26.349 19715505

[20] Kasagi S, Mizusawa K, Murakami N, Andoh T, Furufuji S, Kawamura S, et al Molecular and functional characterization of opsins in barfin flounder (*Verasper moseri*). Gene. 2015;556(2):182–91. 10.1016/j.gene.2014.11.054 25433330

[21] Kranz AM, Forgan LG, Cole GL, Endler JA. Light environment change induces differential expression of guppy opsins in a multi‐generational evolution experiment. Evolution. 2018 10.1111/evo.13519 29920667

[22] Ehlman SM, Sandkam BA, Breden F, Sih A. Developmental plasticity in vision and behavior may help guppies overcome increased turbidity. J Comp Physiol A. 2015;201(12):1125–35. 10.1007/s00359-015-1041-4 26427995

[23] Nandamuri SP, Yourick MR, Carleton KL. Adult plasticity in African cichlids: Rapid changes in opsin expression in response to environmental light differences. Mol Ecol. 2017;26(21):6036–52. 10.1111/mec.14357 28926160 PMC5690868

[24] Fuller RC, Claricoates KM. Rapid light-induced shifts in opsin expression: finding new opsins, discerning mechanisms of change, and implications for visual sensitivity. Mol Ecol. 2011;20(16):3321–35. 10.1111/j.1365-294X.2011.05180.x 21749514

[25] Chang C-H, Yan HY. Plasticity of opsin gene expression in the adult red shiner (*Cyprinella lutrensis*) in response to turbid habitats. PLoS One. 2019;14(4):e0215376 10.1371/journal.pone.0215376 30978235 PMC6461250

[26] Watson CT, Gray SM, Hoffmann M, Lubieniecki KP, Joy JB, Sandkam BA, et al Gene duplication and divergence of long wavelength-sensitive opsin genes in the guppy, *Poecilia reticulata*. J Mol Evol. 2011;72(2):240–22. 10.1007/s00239-010-9426-z 21170644

[27] Watson CT, Lubieniecki KP, Loew E, Davidson WS, Breden F. Genomic organization of duplicated short wave-sensitive and long wave-sensitive opsin genes in the green swordtail, *Xiphophorus helleri*. BMC Evol Biol. 2010;10:87 10.1186/1471-2148-10-87 20353595 PMC3087554

[28] Sandkam B, Dalton B, Breden F, Carleton K. Reviewing guppy color vision: integrating the molecular and physiological variation in visual tuning of a classic system for sensory drive. Curr Zool. 2018;64(4):535–45. 10.1093/cz/zoy047 30108634 PMC6084590

[29] Sandkam BA, Joy JB, Watson CT, Breden F. Genomic environment impacts color vision evolution in a family with visually based sexual selection. Genome Biol Evol. 2017;9(11):3100–7. 10.1093/gbe/evx228 29121209 PMC5714168

[30] Francis RA. A Handbook of Global Freshwater Invasive Species. New York: Earthscan; 2012.

[31] Pyke GH. Plague minnow or mosquito fish? A review of the biology and impacts of introduced *Gambusia* species. Annu Rev Ecol Evol Syst. 2008;39:171–91. 10.1146/annurev.ecolsys.39.110707.173451

[32] Friesen CN, Ramsey ME, Cummings ME. Differential sensitivity to estrogen-induced opsin expression in two poeciliid freshwater fish species. Gen Comp Endocrinol. 2017;246:200–10. 10.1016/j.ygcen.2016.12.009 28013033

[33] Lee F, Simon KS, Perry GLW. Prey selectivity and ontogenetic diet shift of the globally invasive western mosquitofish (*Gambusia affinis*) in agriculturally impacted streams. Ecol Freshw Fish. 2018;27(3):822–33. 10.1111/eff.12395

[34] Kodama I, Yamanaka A, Endo K, Koya Y. Role of the yellow spot around the urogenital opening of female mosquitofish (*Gambusia affinis*) as a cue for copulation. Zool Sci. 2008;25(12):1199–204. 10.2108/zsj.25.1199 19267646

[35] Polverino G, Liao JC, Porfiri M. Mosquitofish (*Gambusia affinis*) preference and behavioral response to animated images of conspecifics altered in their color, aspect ratio, and swimming depth. PLoS ONE. 2013;8(1):e54315 10.1371/journal.pone.0054315 23342131 PMC3546983

[36] Casner AM, Fackelman HC, Degtyareva O, Kight SL. Do female western mosquitofish, *Gambusia affinis*, prefer ornaments that males lack? Ethology. 2016;122(7):561–70. 10.1111/eth.12507

[37] Madliger CL. Toward improved conservation management: a consideration of sensory ecology. Biodivers Conserv. 2012;21:3277–86. 10.1007/s10531-012-0363-6

[38] Terakita A. The opsins. Genome Biol. 2005;6(3):213–22. 10.1186/gb-2005-6-3-213 15774036 PMC1088937

[39] Bolger AM, Lohse M, Usadel B. Trimmomatic: a flexible trimmer for Illumina sequence data. Bioinformatics. 2014;30(15):2114–20. 10.1093/bioinformatics/btu170 24695404 PMC4103590

[40] Grabherr MG, Haas BJ, Yassour M, Levin JZ, Thompson DA, Amit I, et al Trinity: reconstructing a full-length transcriptome without a genome from RNA-Seq data. Nat Biotechnol. 2011;29(7):644–52. 10.1038/nbt.1883 21572440 PMC3571712

[41] Fu L, Niu B, Zhu Z, Wu S, Li W. CD-HIT: accelerated for clustering the next-generation sequencing data. Bioinformatics. 2012;28(23):3150–2. 10.1093/bioinformatics/bts565 23060610 PMC3516142

[42] Li W, Jaroszewski L, Godzik A. Clustering of highly homologous sequences to reduce the size of large protein databases. Bioinformatics. 2001;17(3):282–3. 10.1093/bioinformatics/17.3.282 11294794

[43] Langmead B, Salzberg SL. Fast gapped-read alignment with Bowtie 2. Nat Methods. 2012;9:357–9. 10.1038/nmeth.1923 22388286 PMC3322381

[44] Li B, Dewey CN. RSEM: accurate transcript quantification from RNA-Seq data with or without a reference genome. BMC Bioinformatics. 2011;12:323 10.1186/1471-2105-12-323 21816040 PMC3163565

[45] Camacho C, Coulouris G, Avagyan V, Ma N, Papadopoulos J, Bealer K, et al BLAST+: architecture and applications. BMC Bioinformatics. 2009;10:421 10.1186/1471-2105-10-421 20003500 PMC2803857

[46] Hoffberg SL, Troendle NJ, Glenn TC, Mahmud O, Louha S, Chalopin D, et al A high-quality reference genome for the invasive mosquitofish *Gambusia affinis* using a Chicago library. G3-Genes Genomes Genet. 2018;8(6):1855–61. 10.1534/g3.118.200101 29703783 PMC5982815

[47] Ranwez V, Douzery EJP, Cambon C, Chantret N, Delsuc F. MACSE v2: toolkit for the alignment of coding sequences accounting for frameshifts and stop codons. Mol Biol Evol. 2018;35(10):2582–4. 10.1093/molbev/msy159 30165589 PMC6188553

[48] Stamatakis A. RAxML version 8: a tool for phylogenetic analysis and post-analysis of large phylogenies. Bioinformatics. 2014;30(9):1312–3. 10.1093/bioinformatics/btu033 24451623 PMC3998144

[49] Abadi S, Azouri D, Pupko T, Mayrose I. Model selection may not be a mandatory step for phylogeny reconstruction. Nat Commun. 2019;10:934 10.1038/s41467-019-08822-w 30804347 PMC6389923

[50] Felsenstein J. Confidence limits on phylogenies: an approach using the bootstrap. Evolution. 1985;39(4):783–91. 10.1111/j.1558-5646.1985.tb00420.x 28561359

[51] Fourment M, Holmes EC. Seqotron: a user-friendly sequence editor for Mac OS X. BMC Res Notes. 2016;9:106 10.1186/s13104-016-1927-4 26887850 PMC4756450

[52] Kawamura S, Kasagi S, Kasai D, Tezuka A, Shoji A, Takahashi A, et al Spectral sensitivity of guppy visual pigments reconstituted in vitro to resolve association of opsins with cone cell types. Vision Res. 2016;127:67–73. 10.1016/j.visres.2016.06.013 27476645

[53] Yokoyama S, Radlwimmer FB. The "five-sites" rule and the evolution of red and green color vision in mammals. Mol Biol Evol. 1998;15(5):560–7. 10.1093/oxfordjournals.molbev.a025956 9580985

[54] Matsumoto Y, Oda S, Mitani H, Kawamura S. Orthologous divergence and paralogous anticonvergence in molecular evolution of triplicated green opsin genes in medaka fish, genus *Oryzias*. Genome Biol Evol. 2020;12(6):911–23. 10.1093/gbe/evaa111 32467976 PMC7337190

[55] Cowing JA, Poopalasundaram S, Wilkie SE, Bowmaker JK, Hunt DM. Spectral tuning and evolution of short wave-sensitive cone pigments in cottoid fish from Lake Baikal. Biochemistry. 2002;41(19):6019–25. 10.1021/bi025656e 11993996

[56] Matsumoto Y, Fukamachi S, Mitani H, Kawamura S. Functional characterization of visual opsin repertoire in Medaka (*Oryzias latipes*). Gene. 2006;371(2):268–78. 10.1016/j.gene.2005.12.005 16460888

[57] Wakefield MJ, Anderson M, Chang E, Wei K-J, Kaul R, Graves JAM, et al Cone visual pigments of monotremes: Filling the phylogenetic gap. Visual Neurosci. 2008;25(3):257–64. 10.1017/S0952523808080255 18598396

[58] Laver CRJ, Taylor JS. RT-qPCR reveals opsin gene upregulation associated with age and sex in guppies (*Poecilia reticulata*)—a species with color-based sexual selection and 11 visual-opsin genes. BMC Evol Biol. 2011;11:81 10.1186/1471-2148-11-81 21447186 PMC3078887

[59] Yokoyama S, Starmer WT, Liu Y, Tada T, Britt L. Extraordinarily low evolutionary rates of short wavelength-sensitive opsin pseudogenes. Gene. 2014;534(1):93–9. 10.1016/j.gene.2013.09.114 24125953 PMC3852691

[60] Kasagi S, Mizusawa K, Takahashi A. Green‐shifting of SWS2A opsin sensitivity and loss of function of RH2‐A opsin in flounders, genus *Verasper*. Ecol Evol. 2018;8(2):1399–410. 10.1002/ece3.3745 29375806 PMC5773313

[61] Leech DM, Boeing WJ, Cooke SL, Williamson CE, Torres L. UV‐enhanced fish predation and the differential migration of zooplankton to UV radiation and fish. Limnol Oceanogr. 2009;54(4):1152–61. 10.4319/lo.2009.54.4.1152

[62] Loew ER, McFarland WN, Mills EL, Hunter D. A chromatic action spectrum for planktonic predation by juvenile yellow perch, *Perca flavescens*. Can J Zool. 1993;71(2):384–6. 10.1139/z93-053

[63] Flamarique IN. Diminished foraging performance of a mutant zebrafish with reduced population of ultraviolet cones. Proc R Soc B. 2016;283(1826):20160058 10.1098/rspb.2016.0058 26936243 PMC4810871

[64] Flamarique IN. Opsin switch reveals function of the ultraviolet cone in fish foraging. Proc R Soc B. 2013;280(1752):20122490 10.1098/rspb.2012.2490 23222448 PMC3574309

[65] Britt LL, Loew ER, McFarland WN. Visual pigments in the early life stages of Pacific northwest marine fishes. J Exp Biol. 2001;204:2581–7. 11511674 10.1242/jeb.204.14.2581

[66] McFarland WN, Loew ER. Ultraviolet visual pigments in marine fishes of the family pomacentridae. Vision Res. 1994;34(11):1393–6. 10.1016/0042-6989(94)90138-4 8023448

[67] Shand J, Hart NS, Thomas N, Partridge JC. Developmental changes in the cone visual pigments of black bream *Acanthopagrus butcheri*. J Exp Biol. 2002;205:3661–7. 12409492 10.1242/jeb.205.23.3661

[68] Cheng CL, Flamarique IN. Chromatic organization of cone photoreceptors in the retina of rainbow trout: single cones irreversibly switch from UV (SWS1) to blue (SWS2) light sensitive opsin during natural development. J Exp Biol. 2007;210:4123–35. 10.1242/jeb.009217 18025012

[69] Shand J, Partridge JC, Archer SN, Potts GW, Lythgoe JN. Spectral absorbance changes in the violet/blue sensitive cones of the juvenile pollack, *Pollachius pollachius*. J Comp Physiol A. 1988;163:699–703. 10.1007/BF00603854

[70] Matsumoto T, Ishibashi Y. Sequence analysis and expression patterns of opsin genes in the longtooth grouper *Epinephelus bruneus*. Fish Sci. 2016;82:17–27. 10.1007/s12562-015-0936-x

[71] García‐Berthou E. Food of introduced mosquitofish: ontogenetic diet shift and prey selection. J Fish Biol. 1999;55(1):135–47. 10.1111/j.1095-8649.1999.tb00663.x

[72] Tsurui-Sato K, Fujimoto S, Deki O, Suzuki T, Tatsuta H, Tsuji K. Reproductive interference in live-bearing fish: the male guppy is a potential biological agent for eradicating invasive mosquitofish. Sci Rep. 2019;9:5439 10.1038/s41598-019-41858-y 30931976 PMC6443680

[73] Abrahams MV, Bassett DK, Montgomery JC. Sensory biology as a risk factor for invasion success and native fish decline. Trans Am Fish Soc. 2017;146(6):1238–44. 10.1080/00028487.2017.1353545

