# Supplementary figures and images for: Phylogenetic analysis and ontogenetic changes in the cone opsins of the western mosquitofish (Gambusia affinis)
Source: PLoS One. 2020 Oct 13;15(10):e0240313. doi: 10.1371/journal.pone.0240313 (PMC7553354; doi:10.1371/journal.pone.0240313)

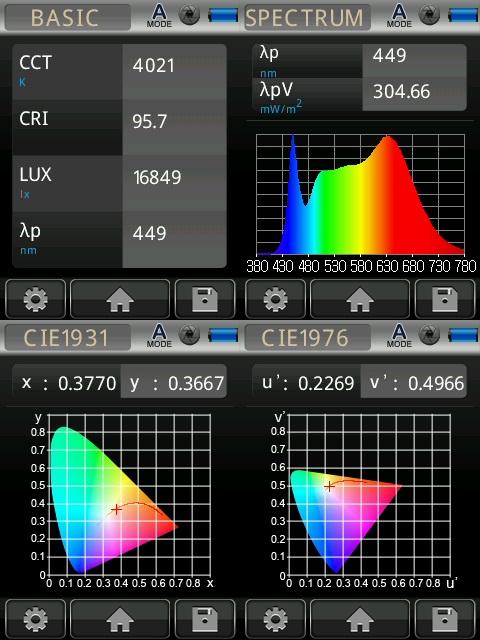

Supplement: S1 Fig — (JPG) [file pone.0240313.s001.jpg]

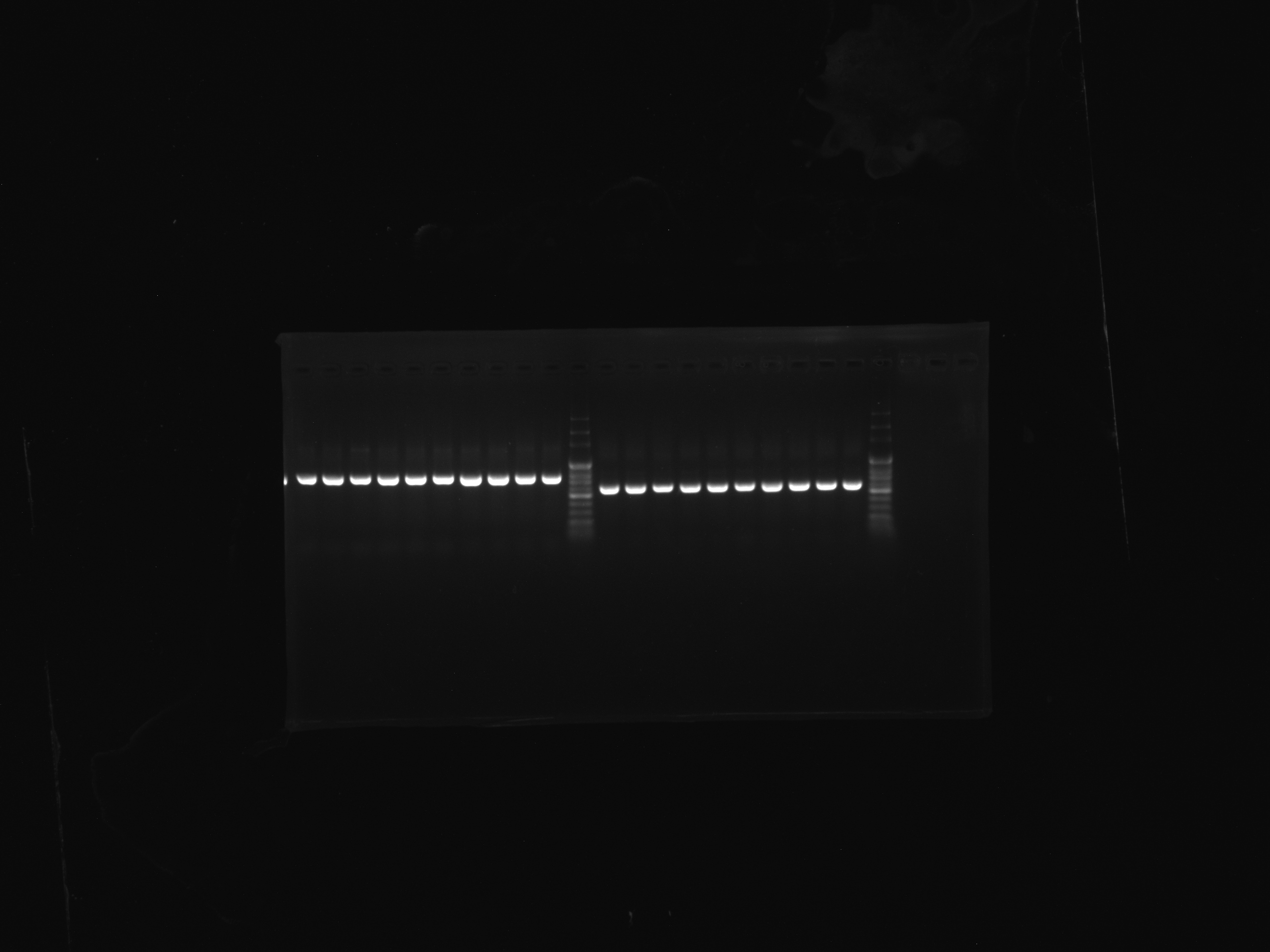

Supplement: S1 Original image — (JPG) [file pone.0240313.s005.jpg]

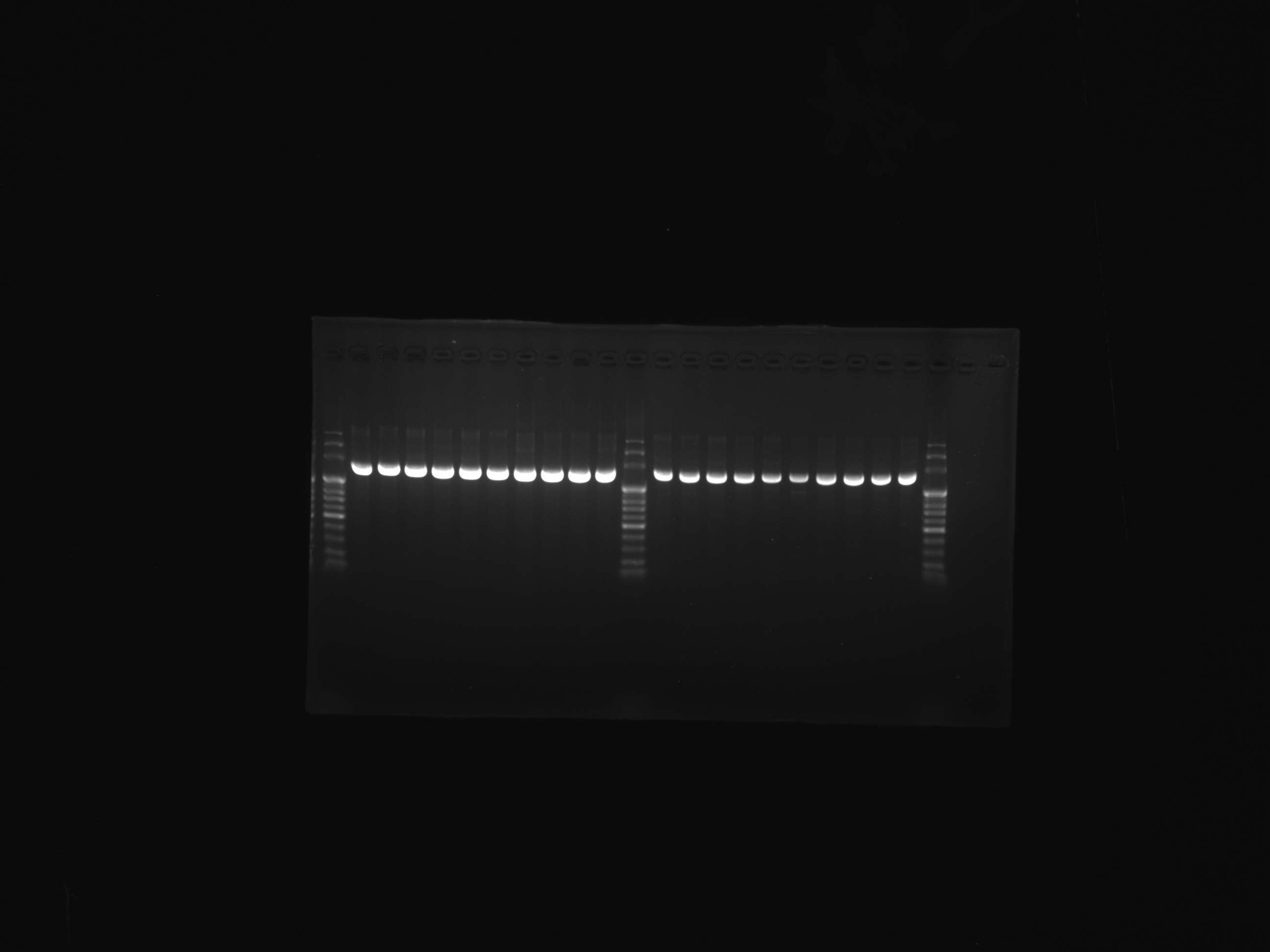

Supplement: S2 Original image — (JPG) [file pone.0240313.s006.jpg]
