# Supplementary material for: Phylogenetic analysis and ontogenetic changes in the cone opsins of the western mosquitofish (Gambusia affinis)
Source: PLoS One. 2020 Oct 13;15(10):e0240313. doi: 10.1371/journal.pone.0240313 (PMC7553354; doi:10.1371/journal.pone.0240313)
Supplement: S2 Fig — (PDF) [file pone.0240313.s002.pdf]

```
                                    10        20        30        40        50
                           ....|....|....|....|....|....|....|....|....|....|
Published G. affinis scaffold  ATGGCAGAAGATTGGGGAAAACAGGCGCTTGCTCCGTGGAAGAACAATGA
Amplified DNA/cDNA LWS-R       --------------------------GCTTGCTCCGTGGAAGAACAATGA
KX768568 guppy LWS-R gene      ATGGCAGAAGATTGGGGAAAGCAGGCGTTTGCTCCCTGGAAGAACAATGA
KX768568 guppy LWS-R cDNA      ATGGCAGAAGATTGGGGAAAGCAGGCGTTTGCTCCCTGGAAGAACAATGA

                                    60        70        80        90       100
                           ....|....|....|....|....|....|....|....|....|....|
Published G. affinis scaffold  AGAAACTACAAGGGGCTCTGCTTTCACATACACAAACAGCAATCACACAA
Amplified DNA/cDNA LWS-R       AGAAACTACAAGGGGCTCTGCTTTCACATACACAAACAGCAATCACACAA
KX768568 guppy LWS-R gene      AGAAACTACAAGGGGCTCTGCTTTCACATACACAAACAGCAATCATACAA
KX768568 guppy LWS-R cDNA      AGAAACTACAAGGGGCTCTGCTTTCACATACACAAACAGCAATCATACAA

                                   110       120       130       140       150
                           ....|....|....|....|....|....|....|....|....|....|
Published G. affinis scaffold  AAAGTATGCTTTTAATTTTGAACCAATCTGGGTTAAAGTTAGTGTTATCA
Amplified DNA/cDNA LWS-R       AAAGTATGCTTTTAATTTTGAACCAATCTGGGTTAAAGTTAGTGTTATCA
KX768568 guppy LWS-R gene      GAGGTATGCTTTTAATTTTGAACCAATTTGGGTTAAAGTTAGTGTTATCA
KX768568 guppy LWS-R cDNA      GA------------------------------------------------

                                   160       170       180       190       200
                           ....|....|....|....|....|....|....|....|....|....|
Published G. affinis scaffold  GAGATTAAATTAATATGTTAAATATCTGTGGATGAGTTTGTACAGTTTTA
Amplified DNA/cDNA LWS-R       GAGATTAAATTAATATGTTAAATATCTGTGGATGAGTTTGTACAGTTTTA
KX768568 guppy LWS-R gene      GAGATTAAATTAATATTTTAAACATCTGTGGAAGAGTTTGTACAGTTTTA
KX768568 guppy LWS-R cDNA      --------------------------------------------------

                                   210       220       230       240       250
                           ....|....|....|....|....|....|....|....|....|....|
Published G. affinis scaffold  CAATCTACAGTGTAAGCAGAATGAC--ATACTGTAGTTTGTGCAGTGATA
Amplified DNA/cDNA LWS-R       CAATCTACAGTGTAAGCAGAATGAC--ATACTGTAGTTTGTGCAGTGATA
KX768568 guppy LWS-R gene      CAATTTGCAGTGTAAGCAGAATGACATATACTGTAGTTTGTGCAGTGATA
KX768568 guppy LWS-R cDNA      --------------------------------------------------

                                   260       270       280       290       300
                           ....|....|....|....|....|....|....|....|....|....|
Published G. affinis scaffold  TTTAGACAAATGCTTGCTGGCACAAAGTAAACTTTAGTGAAGTGTTTGAA
Amplified DNA/cDNA LWS-R       TTTAGACAAATGCTTGCTGGCACAAAGTAAACTTTAGTGAAGTGTTTGAA
KX768568 guppy LWS-R gene      TTTA---AAGTGTCTGCTGGCAC-AAGTAAACTTTAGTGAAGTGTTTGAC
KX768568 guppy LWS-R cDNA      --------------------------------------------------

                                   310       320       330       340       350
                           ....|....|....|....|....|....|....|....|....|....|
Published G. affinis scaffold  ATTTGATTTATTAGTTCACTTCGATTTGAAGACAAACTAATTAATCTGCA
Amplified DNA/cDNA LWS-R       ATTTGATTTATTAGTTCACTTCGATTTGAAGACAAACTAATTAATCTGCA
KX768568 guppy LWS-R gene      ATTTTATTCATTAGCTGACTTTTATTTGAAGACAAACTAATTAATCTGCA
KX768568 guppy LWS-R cDNA      --------------------------------------------------

                                   360       370       380       390       400
                           ....|....|....|....|....|....|....|....|....|....|
Published G. affinis scaffold  TTTACACAATATTTTCCTTGCCTGAATGCCAGTAAGTAGTATGACAGTTG
Amplified DNA/cDNA LWS-R       TTTACACAATATTTTCCTTGCCTGAATGCCAGTAAGTAGTATGACAGTTG
KX768568 guppy LWS-R gene      TTTACACAATATTTTCCTTGC----ATGCCAGTAAGTAATCTGATTGTTG
KX768568 guppy LWS-R cDNA      --------------------------------------------------

                                   410       420       430       440       450
                           ....|....|....|....|....|....|....|....|....|....|
Published G. affinis scaffold  AAGA--CTAAGATGTGAACT--TGCTTCCAGATCCTTTTGAGGGATCAAA
Amplified DNA/cDNA LWS-R       AAGA--CTAAGATGTGAACT--TGCTTCCAGATCCTTTTGAGGGATCAAA
KX768568 guppy LWS-R gene      AAGACTCTAAGATGTGAACTTGTGCTTCCAGATCCTTTTGAGGGACCAAA
KX768568 guppy LWS-R cDNA      ------------------------------GATCCTTTTGAGGGACCAAA

                                   460       470       480       490       500
                           ....|....|....|....|....|....|....|....|....|....|
Published G. affinis scaffold  CTACCACATCACTCCTCAATGGGTTTACAATATAACAACAGTCTGGATGT
Amplified DNA/cDNA LWS-R       CTACCACATCACTCCTCAATGGGTTTACAATATAACAACAGTCTGGATGT
KX768568 guppy LWS-R gene      CTACCATATCGCTCCTCGATGGGTTTACAACATCACAACAGTCTGGATGT
KX768568 guppy LWS-R cDNA      CTACCATATCGCTCCTCGATGGGTTTACAACATCACAACAGTCTGGATGT

                                   510       520       530       540       550
                           ....|....|....|....|....|....|....|....|....|....|
Published G. affinis scaffold  GTTTTGTGGTCGTCTTATCACTCTTCACAAATCGCCTGGTCTTGGCAGCC
Amplified DNA/cDNA LWS-R       GTTTTGTGGTCGTCTTATCACTCTTCACAAATGGCCTGGTCTTGGCAGCC
KX768568 guppy LWS-R gene      GTTTTGTGGTCGTCTTAGCAGTCTTCACAAATGGTCTGGTCTTGGTAGCC
```

```
KX768568 guppy LWS-R cDNA     GTTTTGTGGTCGTCTTAGCAGTCTTCACAAATGGTCTGGTCTTGGTAGCC

                                       560       570       580       590       600
                              ....|....|....|....|....|....|....|....|....|....|
Published G. affinis scaffold ACAGCAAAGTTCAAGAAACTCCGTCATCCCCTGAACTGGATCTTAGTCAG
Amplified DNA/cDNA LWS-R      ACAGCAAAGTTCAAGAAACTCCGTCATCCCCTGAACTGGATCTTAGTCAG
KX768568 guppy LWS-R gene     ACAGCAAGGTTCAAGAAACTCCGTCATCCCCTGAACTGGATCTTAGTCAA
KX768568 guppy LWS-R cDNA     ACAGCAAGGTTCAAGAAACTCCGTCATCCCCTGAACTGGATCTTAGTCAA

                                       610       620       630       640       650
                              ....|....|....|....|....|....|....|....|....|....|
Published G. affinis scaffold CCTTACCATTGCAGATCTTGGAGAGACGGTTTTTGCCAGCACCATAAGCG
Amplified DNA/cDNA LWS-R      CCTTACCATTGCAGATCTTGGAGAGACGGTTTTTGCCAGCACCATAAGCG
KX768568 guppy LWS-R gene     CCTTGCCATTGCCGACCTCGGAGAGACGGTCTTTGCCAGCACCATCAGCG
KX768568 guppy LWS-R cDNA     CCTTGCCATTGCCGACCTCGGAGAGACGGTCTTTGCCAGCACCATCAGCG

                                       660       670       680       690       700
                              ....|....|....|....|....|....|....|....|....|....|
Published G. affinis scaffold TGTGCAACCAGTTTTTTGGATACTTTATTTTGGGACATCCAATGTGCGTC
Amplified DNA/cDNA LWS-R      TGTGCAACCAGTTTTTTGGATACTTTATTTTGGGACATCCAATGTGCGTC
KX768568 guppy LWS-R gene     TGTGCAACCAGTCTTTTGGATATTTTATTTTGGGACATCCAATGTGCGTC
KX768568 guppy LWS-R cDNA     TGTGCAACCAGTCTTTTGGATATTTTATTTTGGGACATCCAATGTGCGTC

                                       710       720       730       740       750
                              ....|....|....|....|....|....|....|....|....|....|
Published G. affinis scaffold TTTGAAGGCTACGTTGTCTCGAATTGTGGTATTGCTGCTCTTTGGTCCCT
Amplified DNA/cDNA LWS-R      TTTGAAGGCTACGTTGTCTCGAATTGTGGTATTGCTGCTCTTTGGTCCCT
KX768568 guppy LWS-R gene     TTTGAAGGCTACGTTGTCTCGACTTGTGGTATTGCTGCTCTTTGGTCCCT
KX768568 guppy LWS-R cDNA     TTTGAAGGCTACGTTGTCTCGACTTGTGGTATTGCTGCTCTTTGGTCCCT

                                       760       770       780       790       800
                              ....|....|....|....|....|....|....|....|....|....|
Published G. affinis scaffold GACTGTCATCTCTTTGGAGAGATGGATTGTTGTATGCAAACCTTTTGGAA
Amplified DNA/cDNA LWS-R      GACTGTCATCTCTTTGGAGAGATGGATTGTTGTATGCAAACCTTTTGGAA
KX768568 guppy LWS-R gene     GACTGTCATCTCTTGGGAGAGATGGATTGTTGTGTGTAAACCTTTTGGAA
KX768568 guppy LWS-R cDNA     GACTGTCATCTCTTGGGAGAGATGGATTGTTGTGTGTAAACCTTTTGGAA

                                       810       820       830       840       850
                              ....|....|....|....|....|....|....|....|....|....|
Published G. affinis scaffold ATACCAAGTTTGATGCTAAATGGGCCACAGCTGGGATCATGTTCTCCTGG
Amplified DNA/cDNA LWS-R      ATACCAAGTTTGATGCTAAATGGGCCACAGCTGGGATCATGTTCTCCTGG
KX768568 guppy LWS-R gene     ATACAAAGTTTGATGCCAAATGGGCGGCAGCTGGGATCATGTTCTCCTGG
KX768568 guppy LWS-R cDNA     ATACAAAGTTTGATGCCAAATGGGCGGCAGCTGGGATCATGTTCTCCTGG

                                       860       870       880       890       900
                              ....|....|....|....|....|....|....|....|....|....|
Published G. affinis scaffold GTCTGGTCGGCAGTGTGGTGTGCTCCTCCTGTCTTTGGATGGAGCAGGTA
Amplified DNA/cDNA LWS-R      GTCTGGTCGGCAGTGTGGTGTGCTCCTCCTGTCTTTGGATGGAGCAGGTA
KX768568 guppy LWS-R gene     GTCTGGTCGGCAGTGTGGTGTGCTCCTCCCGTCTTTGGATGGAGCAGGTA
KX768568 guppy LWS-R cDNA     GTCTGGTCGGCAGTGTGGTGTGCTCCTCCCGTCTTTGGATGGAGCAGGTA

                                       910       920       930       940       950
                              ....|....|....|....|....|....|....|....|....|....|
Published G. affinis scaffold CTGGCCTCATGGACTGAAAACTTTCTGTGGACCCAATATGTTCAGTGGAA
Amplified DNA/cDNA LWS-R      CTGGCCTCATGGACTGAAAACTTTCTGTGGACCCAATATGTTCAGTGGAA
KX768568 guppy LWS-R gene     CTGGCCCCATGGGTTGAAAACGTCCTGTGGACCCGATGTGTTCAGTGGAA
KX768568 guppy LWS-R cDNA     CTGGCCCCATGGGTTGAAAACGTCCTGTGGACCCGATGTGTTCAGTGGAA

                                       960       970       980       990      1000
                              ....|....|....|....|....|....|....|....|....|....|
Published G. affinis scaffold GTGAGGGCCCTGGTGTCAAATCCTACATGATTGTCCTCATGATTACATGC
Amplified DNA/cDNA LWS-R      GTGAGGGCCCTGGTGTCAAATCCTACATGATTGTCCTCATGATTACATGC
KX768568 guppy LWS-R gene     GTGAGGACCCCGGTGTCAAGTCCTACATGATTGTCCTCATGATTACATGC
KX768568 guppy LWS-R cDNA     GTGAGGACCCCGGTGTCAAGTCCTACATGATTGTCCTCATGATTACATGC

                                      1010      1020      1030      1040      1050
                              ....|....|....|....|....|....|....|....|....|....|
Published G. affinis scaffold TGCATCATTCCTCTGGCTGTCGTCATCTTGTGCTACCTGGCAGCGTGGTT
Amplified DNA/cDNA LWS-R      TGCATCATTCCTCTGGCTGTCGTCATCTTGTGCTACCTGGCAGCGTGGTT
KX768568 guppy LWS-R gene     TGCATCACTCCTCTGGCTGTCATCATCTTGTGCTACCTGGCAGTGTGGTT
KX768568 guppy LWS-R cDNA     TGCATCACTCCTCTGGCTGTCATCATCTTGTGCTACCTGGCAGTGTGGTT

                                      1060      1070      1080      1090      1100
                              ....|....|....|....|....|....|....|....|....|....|
Published G. affinis scaffold GGCCATTTGTGACATTGCTATGCAGCAGAAGGAATGCAAGTCGACCCAGA
```

```
Amplified DNA/cDNA LWS-R      GGCCATTTGTGACATTGCTATGCAGCAGAAGGAATGCAAGTCGACCCAGA
KX768568 guppy LWS-R gene     GGCCATCCGTGACATTGCTATGCAGCAGAAGGAATGCGAGTCGACCCAGA
KX768568 guppy LWS-R cDNA     GGCCATCCGTGACATTGCTATGCAGCAGAAGGAATGCGAGTCGACCCAGA

                                       1110      1120      1130      1140      1150
                              ....|....|....|....|....|....|....|....|....|....|
Published G. affinis scaffold ACGCCCAGAAGGAAGTATCTAGGATGGTTGTTGTCATGATCTTGGCTTAT
Amplified DNA/cDNA LWS-R      ACGCCCAGAAGGAAGTATCTAGGATGGTTGTTGTCATGATCTTGGCTTAT
KX768568 guppy LWS-R gene     ACGCCCAGAAGGAAGTATCTAGGATGGTTGTTGTCATGATCTTGGCTTAT
KX768568 guppy LWS-R cDNA     ACGCCCAGAAGGAAGTATCTAGGATGGTTGTTGTCATGATCTTGGCTTAT

                                       1160      1170      1180      1190      1200
                              ....|....|....|....|....|....|....|....|....|....|
Published G. affinis scaffold TGTTCATGTTGGGGA-----------------------------------
Amplified DNA/cDNA LWS-R      TGTTCATGTTGGGGA-----------------------------------
KX768568 guppy LWS-R gene     TGTGTATGTTGGGGACCTTACACCTTTTTTGCTTGCTTTGCCGCAGCCAA
KX768568 guppy LWS-R cDNA     TGTGTATGTTGGGGACCTTACACCTTTTTTGCTTGCTTTGCCGCAGCCAA

                                       1210      1220      1230      1240      1250
                              ....|....|....|....|....|....|....|....|....|....|
Published G. affinis scaffold -----------CCTTCCACCCTTTAGCTGCTGCCATGCCTGTATACTTTG
Amplified DNA/cDNA LWS-R      -----------CCTTCCACCCTTTAGCTGCTGCCATGCCTGTATACTTTG
KX768568 guppy LWS-R gene     CCCCGGATATGCCTTCCACCCGTTAGCCGCTGCCATGCCTGCATACTTTG
KX768568 guppy LWS-R cDNA     CCCCGGATATGCCTTCCACCCGTTAGCCGCTGCCATGCCTGCATACTTTG

                                       1260      1270      1280      1290      1300
                              ....|....|....|....|....|....|....|....|....|....|
Published G. affinis scaffold CCAAGAGTGCCACCATCTAACACCTGGTCATCTATGTCTTCATGAACACA
Amplified DNA/cDNA LWS-R      CCAAGAGTGCCACCATCTAACACCTGGTCATCTATGTCTTCATGAACACA
KX768568 guppy LWS-R gene     CCAAAAGTGCCACCATCTACAACCCGGTCATCTATGTCTTCATGAACCGA
KX768568 guppy LWS-R cDNA     CCAAAAGTGCCACCATCTACAACCCGGTCATCTATGTCTTCATGAACCGA

                                       1310      1320      1330      1340      1350
                              ....|....|....|....|....|....|....|....|....|....|
Published G. affinis scaffold CAGTTCCACACATTCATCATGCAGCTCTTTGGGAAAGAGGTGGATGAC-G
Amplified DNA/cDNA LWS-R      CAGTTCCACACATTCATCATGCAGCTCTTTGGGAAAGAGGTGGATGAC-G
KX768568 guppy LWS-R gene     CAGTTCCGCACATGCATCATGCGGCTTTTTGGGAAAGAGGTGGATGACGG
KX768568 guppy LWS-R cDNA     CAGTTCCGCACATGCATCATGCGGCTTTTTGGGAAAGAGGTGGATGACGG

                                       1360      1370      1380      1390      1400
                              ....|....|....|....|....|....|....|....|....|....|
Published G. affinis scaffold CTCTGAAGTGTCCACATCAAAGACAGAGGAGTACTTTGTTTCTCATCTGT
Amplified DNA/cDNA LWS-R      CTCTGAAGTGTCCACATCAAAGACAGAGGAGTA-----------------
KX768568 guppy LWS-R gene     CTCTGAAGTGTCCACATCAAAGACAGAGGTCTCCTCTGTGGCTCCT----
KX768568 guppy LWS-R cDNA     CTCTGAAGTGTCCACATCAAAGACAGAGGTCTCCTCTGTGGCTCCT----

                                       1410      1420      1430      1440      1450
                              ....|....|....|....|....|....|....|....|....|....|
Published G. affinis scaffold TCTTGAATTTATTCTAGATCTGTGCTTTCTAAGAATTACTTTGCCTGCTA
Amplified DNA/cDNA LWS-R      --------------------------------------------------
KX768568 guppy LWS-R gene     --------------------------------GAATAAACCTTCATATCC
KX768568 guppy LWS-R cDNA     --------------------------------GAATAA------------

                                       1460      1470      1480      1490      1500
                              ....|....|....|....|....|....|....|....|....|....|
Published G. affinis scaffold TGTGTTGCCAGACAGGTTCTATCTGAGTGATTTATTTAAAGCATTGGTAT
Amplified DNA/cDNA LWS-R      --------------------------------------------------
KX768568 guppy LWS-R gene     TCTGTTG-------------------------------------TTTTTT
KX768568 guppy LWS-R cDNA     --------------------------------------------------

                                       1510      1520      1530      1540      1550
                              ....|....|....|....|....|....|....|....|....|....|
Published G. affinis scaffold TACAGTAATCTGACCTGGGTGTGTTTGGATTTCAATATGGTCTTATTTGT
Amplified DNA/cDNA LWS-R      --------------------------------------------------
KX768568 guppy LWS-R gene     TTTAATAATTTGACCTGTATGTA---------AAATATAATTTGTTTTAT
KX768568 guppy LWS-R cDNA     --------------------------------------------------

                                       1560      1570      1580      1590      1600
                              ....|....|....|....|....|....|....|....|....|....|
Published G. affinis scaffold CTGACATCCATTCAAATGTAACAATACAGAAGAAATCTGTAAGGTGGAAA
Amplified DNA/cDNA LWS-R      --------------------------------------------------
KX768568 guppy LWS-R gene     TTTAAAAGCTTAAAAGTGAAGGTTTTGTGTGTCAATGCATGAAATTAACT
KX768568 guppy LWS-R cDNA     --------------------------------------------------

                                       1610
```

```
                                ....|....|....|.
Published G. affinis scaffold   ATAACAGCACGAAATA
Amplified DNA/cDNA LWS-R        ----------------
KX768568 guppy LWS-R gene       TTAATTATTTTAA---
KX768568 guppy LWS-R cDNA       ----------------
```
