# Supplementary material for: Phylogenetic analysis and ontogenetic changes in the cone opsins of the western mosquitofish (Gambusia affinis)
Source: PLoS One. 2020 Oct 13;15(10):e0240313. doi: 10.1371/journal.pone.0240313 (PMC7553354; doi:10.1371/journal.pone.0240313)
Supplement: S1 Appendix — (DOCX) [file pone.0240313.s003.docx]

>Danio_rerio_RH21

ATG------------------------------------------------AACGGGACAGAA---GGGAGCAACTTCTACATCCCCATGTCAAACAGGACTGGGCTTGTGCGA------AGCCCTTATGAT------TACACCCAGTACTATTTGGCAGAACCATGGAAATTCAAGGCTCTGGCTTTCTACATGTTTCTACTGATCATTTTTGGATTTCCCATCAATGTCCTGACTTTGGTGGTTACAGCCCAGCACAAGAAACTCCGACAGCCGCTCAACTACATTTTGGTCAACTTAGCTTTTGCTGGCACAATTATGGTCATCTTTGGATTTACGGTTTCTTTCTACTGCTCACTTGTGGGCTACATGGCTCTTGGTCCATTAGGCTGTGTAATGGAGGGATTCTTCGCAACACTTGGAGGTCAGGTTGCTCTTTGGTCTTTGGTGGTTCTTGCCATTGAGAGGTACATCGTGGTTTGCAAGCCAATGGGAAGCTTCAAATTCTCCGCA---------AACCATGCGATGGCTGGCATTGCTTTCACATGGTTCATGGCCTGTAGCTGTGCAGTCCCCCCACTTTTTGGCTGGTCCAGATATCTTCCGGAAGGGATGCAAACCTCTTGCGGACCAGATTACTACACACTGAATCCTGAATATAACAATGAGTCTTACGTCATGTACATGTTCAGCTGCCACTTTTGCATACCCGTCACCACAATTTTCTTTACCTATGGCAGTCTGGTCTGCACAGTCAAGGCTGCTGCAGCTCAACAGCAGGAATCTGAGTCCACTCAGAAGGCTGAGAGGGAAGTGACACGCATGGTCATCCTGATGGTTTTGGGCTTCTTGTTTGCTTGGGTTCCCTATGCCAGCTTTGCTGCCTGGATCTTCTTTAACAGGGGAGCTGCTTTCTCCGCACAAGCCATGGCTGTTCCAGCCTTCTTCTCTAAGACTTCAGCTGTTTTTAACCCCATCATCTATGTGCTCCTAAACAAACAGTTCCGTAGCTGCATGCTTAACACC---CTTTTCTGT---------------------------------------------------------------GGCAAGAGTCCTCTTGGTGACGATGAGTCTTCC------------TCAGTGTCAACA------AGCAAA---ACAGAG---------------------------------------------------GTGTCCTCTGTGTCTCCTGCATAG------------------------------------------------------------------------------------

>Danio_rerio_RH22

ATG------------------------------------------------AACGGCACCGAA---GGAAACAACTTTTACATTCCTATGTCCAACAGGACTGGGCTAGTGAGG------AGTCCTTACGAA------TACACACAGTATTACCTGGCAGATCCATGGCAGTTTAAGGCACTGGCTTTTTACATGTTTTTTCTGATTTGCTTCGGGCTGCCGATCAACGTGCTCACGCTGTTGGTCACAGCTCAGCACAAAAAGCTGAGACAGCCTTTAAATTATATTTTGGTGAACTTGGCTTTCGCTGGAACAATTATGGCCTTTTTTGGATTCACGGTTACGTTCTATTGCTCCATTAATGGTTATATGGCTCTAGGTCCTACAGGTTGTGCCATTGAAGGCTTCTTTGCTACACTTGGAGGTCAGGTTGCTCTTTGGTCTCTTGTGGTTCTTGCCATTGAGAGGTACATAGTGGTTTGCAAGCCAATGGGAAGCTTCAAATTCTCCTCA---------AACCATGCGATGGCCGGCATTGCCTTCACATGGGTCATGGCCTCTAGCTGTGCAGTCCCCCCACTTTTTGGCTGGTCCCGATACATCCCAGAGGGAATGCAGACATCCTGCGGACCAGATTATTACACCCTGAACCCAGAATTCAACAATGAATCATATGTTTTGTACATGTTCAGCTGTCATTTCTGCGTTCCTGTTACAACCATCTTCTTCACTTATGGAAGCCTCGTTTGCACAGTCAAAGCTGCTGCAGCTCAGCAGCAGGAATCTGAGTCCACTCAGAAGGCTGAGAGGGAAGTAACACGCATGGTCATCCTGATGGTTTTGGGCTTCTTGGTGGCTTGGGTTCCCTATGCCAGCTTTGCGGCCTGGATCTTTTTTAACAGGGGAGCTGCTTTCTCCGCACAAGCCATGGCTATCCCAGCTTTTTTCTCAAAAGCCTCTGCCTTGTTTAACCCTATTATCTATGTGCTCCTAAACAAACAGTTCCGTAGCTGCATGCTCAACACC---CTTTTCTGT---------------------------------------------------------------GGCAAGAGTCCTCTTGGAGATGATGAGTCCTCC------------TCAGTTTCAACA------AGCAAA---ACAGAG---------------------------------------------------GTGTCCTCTGTGTCTCCAGCGTAG------------------------------------------------------------------------------------

>Danio_rerio_RH24

ATG------------------------------------------------AACGGCACTGAA---GGAAACAACTTCTACATCCCCCTGTCCAACAGGACAGGGCTAGCAAGG------AGTCCGTACGAA------TATCCTCAGTATTATCTTGCGGAGCCATGGCAATTTAAACTGCTCGCCGTCTACATGTTCTTCCTCATCTGCTTAGGTTTCCCCATCAATGGCCTTACATTGTTGGTAACAGCTCAACACAAAAAGCTGAGGCAACCTCTCAACTTCATTCTGGTCAATCTGGCTGTGGCTGGCACCATCATGGTTTGTTTTGGATTCACGGTCACTTTCTACACAGCCATTAATGGATACTTTGTTCTGGGACCAACTGGCTGTGCGATTGAGGGATTCATGGCCACACTGGGAGGCGAAGTTGCCCTTTGGTCACTTGTAGTGCTGGCCGTTGAGAGATACATTGTGGTTTGCAAGCCAATGGGGAGTTTCAAATTTTCTGCC---------AGTCACGCTTTCGCAGGATGCGCATTCACATGGGTAATGGCCATGGCTTGTGCAGCTCCCCCTCTGGTTGGTTGGCCCAGGTATATTCCGGAGGGAATGCAGTGTTCATGTGGACCAGACTACTACACCCTGAACCCAGAGTACAACAACGAATCATATGTCCTCTACATGTTCATCTGTCATTTTATACTTCCAGTTACTATAATCTTCTTCACCTATGGGCGACTTGTTTGCACAGTCAAAGCGGCTGCAGCTCAACAGCAGGAATCAGAATCCACTCAGAAGGCTGAGAGAGAAGTGACCAGAATGGTCATCCTGATGGTTCTGGGCTTCCTGATTGCTTGGACTCCTTATGCTACTGTTGCTGCCTGGATCTTCTTTAATAAGGGAGCTGCTTTCAGTGCTCAGTTCATGGCTGTTCCTGCCTTTTTCTCAAAGACCTCTGCATTGTATAACCCTGTCATCTATGTGCTGCTAAATAAACAGTTTCGTAATTGCATGCTGACCACT---CTATTCTGT---------------------------------------------------------------GGAAAGAATCCTCTTGGCGATGATGAATCCTCT------------ACTGTGTCCACC------AGCAAG---ACGGAG---------------------------------------------------GTGTCTTCTGTATCTCCAGCATAG------------------------------------------------------------------------------------

>Danio_rerio_RH23

ATG------------------------------------------------AACGGCACTGAA---GGAAACAACTTCTACATCCCCATGTCCAACAGGACAGGGCTTGTGAGG------AGTCCGTACGAA------TATCCTCAGTATTATCTTGCTGAGCCATGGCAGTTTAAACTGCTCGCCGTCTACATGTTCTTCCTCATGTGCTTTGGGTTTCCCATCAATGGCCTTACATTGGTGGTAACAGCTCAACACAAAAAGCTGAGGCAACCTCTCAACTTCATTCTGGTCAATCTGGCTGTGGCTGGCACCATCATGGTTTGTTTTGGATTCACGGTCACTTTCTACACAGCCATTAATGGATACTTTGTTCTGGGACCAACTGGCTGTGCGATTGAGGGATTCATGGCCACACTGGGAGGGCAAATTTCCCTTTGGTCACTCGTGGTGCTGGCCATTGAGAGATACATTGTGGTTTGCAAGCCAATGGGGAGTTTCAAATTTTCATCC---------AACCATGCTTTCGCTGGGATTGGATTTACATGGATAATGGCCTTGTCTTGCGCAGCTCCTCCTCTGGTTGGCTGGTCAAGGTATATTCCAGAGGGAATGCAGTGTTCATGTGGACCAGACTACTACACCTTGAACCCTGACTACAATAATGAATCATATGTCCTCTACATGTTCTGCTGCCACTTTATATTTCCAGTCACCACAATCTTTTTCACCTATGGACGACTTGTTTGCACAGTCAAAGCGGCTGCAGCTCAACAGCAGGAATCTGAATCCACTCAGAAGGCTGAGAGAGAAGTGACCAGAATGGTCATCCTGATGGTTCTGGGCTTCCTGGTAGCTTGGACTCCTTATGCCAGTGTTGCTGCTTGGATCTTCTTCAATAGAGGAGCTGCTTTCAGTGCTCAGTTCATGGCTGTTCCTGCCTTTTTCTCAAAGAGCTCTTCCATATTCAACCCTATCATTTATGTGCTTCTAAACAAACAGTTTCGTAATTGCATGCTGACCACT---TTATTCTGT---------------------------------------------------------------GGAAAGAATCCTCTCGGTGATGATGAATCCTCT------------ACTGTGTCCACC------AGCAAG---ACGGAG---------------------------------------------------GTGTCTTCTGTATCTCCAGCATAG------------------------------------------------------------------------------------

>OUTGROUP_Danio_rerio_tmt3

ATG------ATTGTGTCCAACTTGAGTGTGCTCAGTTGCAGGAGGAACAGCGCGCTGTGTCTC---GGTGCA---------GTGGAGGGACATTTGGAGGCGTCC---------------TCCTCCTATCGCACCCTCAGTCCGACTGGACACATC---------------------CTCGTGGCGGTGAGCTTGGGATTCATCGGGACTTTCGGATTCCTCAACAACCTCCTGGTCCTCGTGCTCTTCGGCCGATACAAGGTGCTGCGCTCCCCCATCAACTTTCTGCTGGTGAACATCTGCTTGAGCGATCTCCTGGTTTGCGTTCTGGGGACGCCGTTCAGCTTCGCGGCGAGCACGCAGGGGCGATGGCTCATCGGGGACACTGGATGCGTGTGGTACGGCTTTGCCAACTCGCTGTTAGGCATTGTATCGCTCATCTCTCTGGCTGTGCTGTCATACGAACGCTAC---------TGTACAATGATGGGCTCCACAGAGGCTGATGCGACCAACTACAAGAAGGTGATCGGTGGGGTGCTGATGTCCTGGATCTACTCTTTAATTTGGACCCTGCCTCCTCTGTTTGGGTGGAGCCGCTATGGCCCCGAAGGCCCCGGGACAACCTGCTCTGTAGACTGGACGACCAAGACCGCC------AATAACATCTCCTACATCATTTGCCTCTTTATTTTTTGCCTGATCGTCCCCTTCCTGGTCATCATATTCTGCTATGGGAAGCTGCTGCATGCCATTAAACAGGTCAGC------AGTGTGAACACGTCTGTGAGCCGCAAGCGTGAGCACCGTGTCCTACTGATGGTCATCACTATGGTGGTTTTCTATCTGCTGTGCTGGCTCCCCTACGGCATCATGGCCCTGCTGGCCACTTTTGGAGCTCCAGGACTCGTGACGGCGGAAGCCAGCATAGTGCCCTCCATCTTAGCCAAGTCCAGCACAGTCATCAATCCTGTCATCTACATCTTTATGAATAAACAGTTCTACAGGTGTTTCAGAGCTCTT---CTC---------------------------------------------------------------------AACTGCGACAAA---CCACAACGAGGTTCTAGT------------CTGAAAAGCTCT------TCAAAG---ACCAAACCTTTTCGTCCCGGACGCCGCACGGACAACTTCACTTTTATG---------GTTGCTTCAGTGGGACCCAATCAGACAAACCCAGTGGAGGACGGCCCACCATCAGCAGATAACACCAAACCTGCAGTGCTCTCCCTCGTGGCCCATTATAACGGATGA

>Danio_rerio_valopsin

ATG---------------------------------------------------------GAG---GCGTCCTCCGCGGCCGTGAACGCGGTTTCTCCCGCCGAA---------------GACCCGTTCTCC------GCGCCGCTGTCTTCCATCGCGCCC---TGGAACTACAGTGTTCTGGCGGCGCTGATGTTCGTGGTGACCGCGCTGTCTCTGTCCGAGAACTTCACCGTAATGCTGGTCACCTTCAGGTTCCAGCAGCTCCGGCAGCCGCTCAACTACATCATCGTCAACCTGTCTCTGGCCGACTTCCTGGTGTCTCTGACCGGCGGCAGCATCAGCTTCCTCACCAACTATCACGGATATTTCTTCCTGGGGAAATGGGCTTGCGTGCTCGAGGGCTTCGCCGTCACCTTTTTCGGTATTGTGGCTCTGTGGTCTCTCGCCGTTCTGGCGTTCGAGCGGTTTTTCGTGATCTGCCGACCACTAGGGAACATTCGTCTGCGGGGC---------AAACATGCAGCTCTGGGTCTGGTGTTTGTCTGGAGTTTCTCCTTCATCTGGACTGTTCCTCCTGTTCTAGGCTGGAGCAGCTATACTGTCAGCAGGATCGGTACCACCTGTGAACCCAACTGGTATTCAGGAAAC------TTCCACGACCACACCTTCATCATCACGTTATTCAGTACCTGCTTCATCTTCCCTCTGGGAGTGATCATCGTCTGCTACTGCAAACTCATCCGCAAACTCAGAAAGGTCTCCAACACCCATGGCAGGCTGGGTAACGCCAGGAAACCGGAGCGTCAGGTGACCCGTATGGTGGTGGTGATGATCGTGGCATTTATGGTGGCCTGGACGCCGTACGCCGCTTTCTCCATCATCATCACTGCACATCCCAGCATGCACGTGGACCCACGTCTGGCAGCCATTCCTGCATTTGTGGCCAAAACAGCGGCAGTGTACAACCCCATCATCTATGTGTTCATGAACAAACAGTTCAGAAAGTGTCTGGTTCAGCTC---TTGAGTTGCAGTAAAGTGACTGTGGTGGAAGGAAATAATAATCAGACGACCGAACGTGCCGGCATGACCAGCGGGAGCAAC---------ACTGGAGAGATGTCTGCCATCGCTGCTCGCGTCTCCGTC------CCGAAA---ACAGAGGAA---AATCCTGGAGACCGCAGC---ACTTTCAGCCACATCCCCATACCTGAGAACAAAGTGTGTCCCATGTGA------------------------------------------------------------------------------------

>AB223053_Olatipes_RH2_A

ATG---------------------------------------------GAGAACGGCACAGAG---GGCAAGAACTTCTACATCCCCATGAACAATAGGACCGGGCTTGTGAGG------AGTCCTTATGAA------TATCCGCAGTATTATTTGGCAGATCCATGGCAGTTCAAGTTATTGGGCATCTACATGTTTTTCCTGATCCTCACTGGCTTCCCCATCAACGCTCTAACACTCGTGGTCACAGCTCAGAACAAGAAGCTCCGCCAACCTCTCAACTTCATCCTGGTGAACTTGGCTGTGGCTGGACTCATCATGGTCTGCTTTGGATTCACGGTTTGCATTTATTCTTGTATGGTGGGCTATTTCTCTCTGGGACCCCTGGGCTGTACAATCGAGGGATTCATGGCAACACTCGGAGGTCAAGTGTCCCTGTGGTCTCTTGTGGTCTTAGCTATTGAGAGATACATTGTGGTCTGTAAACCCATGGGAAGTTTCAAATTCACTGCA---------ACCCACTCTGCAGCCGGCTGCGCGTTCACCTGGATAATGGCCAGCTCCTGTGCAGTCCCTCCTTTGGTTGGCTGGTCAAGGTATATTCCAGAGGGCATTCAGGTTTCCTGCGGACCTGACTATTACACTTTGGCCCCAGGCTTTAACAACGAATCCTTTGTCATGTACATGTTCACCTGCCACTTCTGTGTGCCTGTCTTCACCATCTTCTTCACCTATGGCAGCCTTGTGATGACAGTAAAGGCTGCTGCAGCCCAGCAGCAAGACTCAGCTTCTACTCAGAAAGCAGAGAAGGAAGTGACTCGGATGTGCTTCCTGATGGTGCTGGGGTTCCTCCTGGCTTGGGTCCCTTATGCTTCCTACGCCGCCTGGATCTTCTTCAACAGAGGAGCCGCCTTCTCAGCCATGTCCATGGCCATCCCATCCTTCTTTTCAAAGAGCTCAGCCCTGTTCAATCCTATCATCTATATTTTGTTGAACAAACAGTTCCGTAACTGCATGCTGGCTACT---ATT---------------------------------------------------------------------GGAATGGGCGGC---ATGGTTGAAGATGAGACT------------TCAGTGTCCACC------AGCAAG---ACAGAA---------------------------------------------------GTCTCTACTGCTGCTTGA------------------------------------------------------------------------------------------

>AB223054_Olatipes_RH2_B

ATG------------------------------------GGTTGGGAGCCTAATGGCACTGAA---GGACAAAACTTCTACATCCCAATGTCCAACCGGACGGGGGTTGTTAGA------AGCCCCTTTGAA------TACCCTCAGTACTACATGGTCGACCCAATTATGTACAAGATTCTGGCTTTCTACATGTTCTTCCTGATCTGCACTGGAACTCCCATCAACGGCTTGACATTGTACGTAACAGCCACCAACAAGAAGCTTCAGCAACCTCTCAACTTCATCCTGGTGAACCTGGCGGTGGCCGGACTCATCATGTGCGCCTTTGGCTTCACCATCACCATCACATCAAGCTTCTATGGCTATTTTGTTCTTGGACCCACCTTCTGTGCCATTGAGGGATTTATGGCCACACTTGGAGGTGAAGTTGCTCTCTGGTCCCTGGTGGTCCTGGCTGTTGAGAGATATATTGTTGTCTGCAAACCCATGGGGAGCTTCAAATTCTCAGGA---------ACTCACGCCGGAGCTGGAGTCCTTCTGACCTGGGTAATGGCTCTGGCTTGTGCTGCTCCTCCACTTTGCGGCTGGTCCAGGTACATCCCTGAAGGCATGCAATGCTCCTGTGGACCTGACTACTACACTCTGGCTCCTGGCTTCAACAATGAGTCATACGTCATATATATGTTTGTTGTGCACTTCTTCGTCCCAGTCTTCCTCATTTTCTTCACTTATGGAAGTCTGGTGTTGACTGTCAAAGCTGCAGCAGCGCAGCAGCAGGACTCAGCTTCTACCCAGAAGGCTGAGAAGGAGGTAACACGTATGTGCCTGTTGATGGTCTTTGGCTTCCTGGTAGCTTGGGTTCCGTACGCCAGCTTTGCTGGCTGGATTTTCCTGAACAAAGGAGCTTCCTTCACTGCCCTGACTGCCTCCATCCCTGCTTTCTTTGCTAAGAGCTCAGCATTGTACAATGCTGTTATCTACGTGCTGCTGAACAAACAGTTCCGTAACTGCATGCTGGCTGCC---ATT---------------------------------------------------------------------GGAATGGGAGGC---TTGGTGGAGGATGAGACC------------TCAGTGTCAACA------AGCAAG---ACAGAA---------------------------------------------------GTCTCAACTGCAGCTTAA------------------------------------------------------------------------------------------

>Danio_rerio_SWS2

ATG------------------------------------AAGCAACAACAGCAAACGCCAGAACTGTTCGAAGACTTCCACATGCCCATCACTTTAGACGTCAGCAACATCTCAGCTTACAGCCCTTTCCTG------GTCCCACAGGACCACCTGGGACACAGTGGCGTATTCATGGGCATGTCCGCTTTTATGCTCTTCCTTTTTATCGCAGGAACTGCCATCAACGTTCTTACCATAGTTTGCACAATTCAATACAAGAAACTCAGATCTCACCTGAACTATATTCTTGTGAACCTTGCCATTTCCAACTTGTGGGTGTCCGTTTTCGGTTCCTCGGTAGCGTTCTACGCCTTTTACAAAAAGTACTTTGTCTTTGGGCCGATAGGATGCAAAATCGAGGGCTTCACTTCAACAATTGGAGGAATGGTGAGTTTGTGGTCTCTTGCTGTGGTGGCGCTGGAAAGGTGGCTGGTCATTTGCAAACCCCTCGGGAACTTTACCTTCAAGACC---------CCTCATGCCATTGCCGGCTGCATACTTCCTTGGTGTATGGCATTGGCTGCTGGACTCCCTCCACTCTTAGGCTGGAGCCGGTATATACCAGAGGGCTTGCAGTGCTCTTGTGGACCTGACTGGTATACGACTAACAACAAATTCAACAATGAATCCTACGTCATGTTCCTCTTCTGCTTTTGCTTTGCGGTTCCTTTCAGCACCATTGTATTCTGTTATGGTCAGCTGCTCATCACTCTCAAATTAGCAGCCAAAGCTCAAGCTGATTCAGCCTCAACCCAGAAGGCTGAGAGGGAGGTGACAAAGATGGTGGTGGTAATGGTGTTCGGCTTCTTGATATGCTGGGGACCATATGCTATCTTTGCAATCTGGGTGGTTTCCAACCGCGGTGCACCTTTTGACCTGAGACTGGCAACCATTCCCTCCTGCCTTTGTAAAGCCTCTACAGTGTACAATCCCGTCATATATGTCTTAATGAACAAACAGTTCCGCTCCTGTATGATGAAGATGGTCTTC---------------------------------------------------------------------AACAAGAAT---------ATTGAGGAAGATGAG------------GCTTCTTCTTCA------TCTCAGGTCACCCAG---------------------------------------------------GTCTCCTCTGTTGCGCCAGAGAAATAA---------------------------------------------------------------------------------

>HQ260684_Preticulata_SWS2A

---------------------------------------------------------------------------------------------------------------------------------------------------------------------------------------------------------------------GGCACTTCCATCAACACCCTCACCATCGTGTGCACCATGCGGTACAAGAAGCTTCGCTCCCACCTCAACTACATCCTGGTGAACTTGGCCTTGGCGAACCTTCTGGTGTCTGTGGTCGGCTCCTTCACCACCTGCTTCTCCTTCACATTCAGATATTTCTTCTTTGGGCCGCTAGCATGCAAGATCGAAGGGTTTGTCGCAACACTAGGGGGTATGGTAAGCCTTTGGTCTTTGGCAGTGGTAGCTTTTGAAAGATGGCTGGTCATCTGCAAACCTCTTGGCAATGTTGCTTTCAAGCCT---------GAACATGCGACGGCTCGCTGCGCAATCACCTGGATTTTCGCGTTGACAGCCTCAGTTCCACCCTTGCTGGGATGGAGCAGGTACATCCCAGAAGGCCTCCAGTGCTCCTGCGGTCCAGACTGGTACACAACCAACAACAAATACAACAACGAGTCCTACGTCATGTTCCTTTTC------TGCTTC------------------------------------------------------------------------------------------------------------------------------------------------------------------------------------------------------------------------------------------------------------------------------------------------------------------------------------------------------------------------------------------------------------------------------------------------------------------------------------------------------------------------------------------------------------------------------------------------------------------------------------------------------------

>AB223057_Olatipes_SWS2_B

ATG------------------------------------AGGGGAAATCGTGTTGTGGAGTTT---CCAGATGACTTTTGGATCCCCATCCCTCTGGATACCAATAATGTCACTGCGCTAAGCCCGTTTCTG------GTCCCCCAGGATCACTTGGGAAGCCCCACCATCTTTTATTCTATGTCAGCATTGATGTTTGTCTTGTTTGTGGCTGGCACTGCCATCAACCTCCTCACTATTGCGTGCACACTTCAGTACAAGAAGCTCCGGTCTCATCTGAACTACATCCTGGTCAACATGGCGGTGGCAAACCTCATTGTTGCTTCTACGGGTTCCTCCACCTGCTTCGTCTGCTTTGCCTTCAAATACATGGTTCTGGGTCCACTGGGCTGCAAGATTGAAGGCTTTACTGCAGCCCTTGGAGGCATGGTGAGCCTCTGGTCTCTTGCTGTAATTGCATTTGAACGGTGGCTGGTTATCTGCAAGCCGCTTGGAAACTTTGTCTTCAAGTCT---------GAGCATGCTTTGCTGTGCTGTGCGTTGACTTGGGTTTGTGGATTGTGTGCTTCAGTTCCACCTCTGGTGGGATGGAGTAGGTACATCCCAGAGGGCATGCAGTGTTCGTGTGGACCAGACTGGTACACAACCGGCAACAAGTTTAACAACGAGTCTTTTGTGATGTTCCTCTTTTGCTTCTGCTTTGCCGTCCCTTTCTCCATCATTGTCTTCTGCTATTCTCAGCTGCTTTTCACTCTGAAAATGGCAGCAAAGGCCCAGGCTGACTCCGCCTCCACTCAGAAGGCAGAGAAGGAGGTTACCAGGATGGTGGTCGTCATGGTTGTTGCTTTCCTGGTTTGCTATGTGCCCTACGCTTCCTTTGCCCTCTGGGTTATCAACAATCGCGGGCAGACATTTGACCTGAGACTTGCGACCATACCCTCCTGTGTGTCGAAGGCCTCCACAGTCTACAACCCCTTCATTTATGTCCTTCTCAACAAGCAGTTCCGCTTGTGCATGAAGAAGATG---CTG---------------------------------------------------------------------GGGATGAGT---------GCAGACGAAGATGAA------------GAGTCCTCAACCAGTCAATCAACC---ACTGAA---------------------------------------------------GTCTCAAAAGTCGGCCCTTCCTAA------------------------------------------------------------------------------------

>KX768623_Hformosa_SWS2B

ATG------------------------------AAGATGAGGACAAGTCGTCAAGAGGAAGTT---CCAGAGGACTTCTGGATCCCCATCCCCCTGGAAACCGACAACATCACAACCCTCAGCCCGTACCTC------GTCCCCCAGGACCATTTAGGGAGCCTGGGGCTTTTTTATTCAATGTCAGCATTAATGTTCGTCTTGTTTGCAGCCGGCACGGCCATCAATATCCTCACAATCGCATGTACTATTCAATACAAGAAGCTCCGCTCCCATCTGAACTACATCCTGGTCAACATGGCTATTGCGAACCTCATCGTCTCGTCCGTGGGCTCTTTTACCTGCTTCTACTGTTTTGCCTTCCGATACATGGCTCTCGGTCCTCTTGGCTGCAAGATTGAAGGATTTACGGCATCTCTTGGTGGCATGGTCAGCCTTTGGTCTCTGGCAGTAATTGCGTTTGAAAGATGGCTGGTTATCTGCAAGCCACTCGGGAACTTTGCCTTCAAGTCG---------GAGCATGCTTTGTTCTTCTGTGCACTTACCTGGTTCTTTGCTTTGTGCGCTGCAGTTCCTCCACTAGTGGGATGGAGTAGGTATATCCCTGAGGGGATGCAGTGTTCATGTGGACCAGACTGGTACACAACGGGCACCAAGTACAACACAGAATCCTTTGTGCTGTTCCTCTTCTGCTTCTGCTTTTCCGTCCCTTTCACTTGCATCGTCTTCTGCTACTCGCAGCTGCTCTTCACACTGAAATCAGCGGCAAAGGCCCAGGCAGAGTCCGCCTCCACCCAGAAGGCAGAGAAAGAGGTGACCAGGATGGTGGTCGTCATGGTCCTGGGCTTCCTGGTGTGCTACATGCCATACGCCTCCTTTGCGCTTTGGGTCGTGAATCATCGTGGACAGACATTTGACCTGAGACTTGCTACCATACCATCCTGTGTCTCAAAAGCCTCCACGGTCTACAATCCTGTGATCTACGTTCTCCTCAATAAGCAGTTCCGCTCTTGCATGAGGAAGATG---CTG---------------------------------------------------------------------GGAATGAGT---------GCAGGCGACGAGGAG------------GAGGCATCTGCAAGTCAGTCGGTC---ACCGAA---------------------------------------------------GTCTCAAAAGTTGGACCCTCTTAA------------------------------------------------------------------------------------

>MN817662_Gaffinis_LWS_1

------------------------------------------CGGCACGAAGATACAACAAGA---GGCTCCGCATTCACATACACAAACAGCAATCAAACCAAA---------------GATCCTTTTGAA------GGACCAAACTACCACATCGCTCCTCGATGGGTTTACAACATCTCCACACTCTGGATGTGTATGGTGGTCGTTTTATCAGTCTTCACCAACGGCCTCGTCTTGGTGGCCACAGCAAAATTCAAGAAACTTCGTCATCCTCTCAACTGGATCTTGGTCAACCTTGCCATTGCTGATCTTGGAGAGACAGTCTTTGCCAGCACCATCAGTGTGTGCAACCAGTACTTTGGATATTTCATTCTGGGACACCCAATGTGTGTCTTTGAAGGCTNTACTGTCTCAACTTGTGGTATTGCTGCTCTTTGGTCCCTGACNATCATCTCTTGGGAGAGATGGATAGTTGTGTGCAAACCCTTTGGAAATGTCAAGTTCGATGCC---------AAGTGGGCCACAGGTGGAATAGTTTTCTCCTGGGTCTGGTCTGCAGYGTGGTGTGCTCCTCCCATCTTTGGATGGAGCAGGTATTGGCCTCATGGACTGAAAACGTCCTGCGGGCCTGATGTGTTCAGTGGAAGCGAAGACCCTGGAGTCCAGTCCTACATGATTGTCCTCATGATTACATGCTGCATCATTCCTCTGGCTATCATCATCTTGTGCTACCTGGCTGTGTGGTTGGCCATCCGTGCTGTTGCTATGCAGCAGAAGGAATCTGAGTCGACCCAGAAGGCTGAGAGAGAAGTGTCCAGGATGGTTGTAGTCATGATCATAGCTTACTGTGTCTGCTGGGGACCCTACACCTTTTTCGCCTGCTTTGCCGCAGCCAACCCCGGATACGCCTTCCATCCTTTGGCTGCTGCCATGCCTGCATACTTTGCCAAAAGCGCCACC---------------------------------------------------------------------------------------------------------------------------------------------------------------------------------------------------------------------------------------------------------------------------------------------------------------------------------------------------------------------------------------

>Danio_rerio_SWS1

ATG------------------------------------------------------------------GACGCGTGGGCCGTTCAATTCGGAAATGCTTCCAAAGTC------------AGCCCCTTCGAG------GGCGAACAATACCACATTGCCCCCAAATGGGCGTTCTACCTGCAGGCAGCTTTCATGGGCTTTGTATTTATCGTGGGCACACCTATGAACGGAATCGTCCTCTTCGTCACGATGAAGTACAAGAAACTCAGGCAGCCTCTCAACTACATCTTGGTAAACATCTCCCTAGCAGGCTTCATTTTCGACACGTTCTCTGTAAGTCAAGTATTCGTCTGCGCTGCTAGAGGTTATTACTTCCTTGGGTATACGTTGTGTGCGATGGAAGCGGCAATGGGATCGATTGCAGGTCTTGTGACGGGATGGTCCTTGGCTGTTCTGGCTTTCGAGAGATATGTGGTCATCTGCAAACCCTTCGGAAGCTTCAAATTCGGACAA---------GGCCAGGCTGTTGGAGCTGTGGTGTTCACCTGGATCATTGGTACCGCCTGTGCTACTCCTCCCTTCTTTGGATGGAGCAGATACATTCCCGAGGGTCTTGGCACTGCCTGCGGACCTGACTGGTACACAAAAAGCGAGGAGTACAACTCAGAGAGCTACACTTACTTCCTTCTCGTCACCTGCTTCATGATGCCAATGACCATCATCATTTTCTCCTACTCACAGCTCTTGGGAGCCCTGCGCGCCGTTGCAGCCCAGCAGGCCGAGTCAGAGTCCACCCAGAAGGCAGAGAGAGAAGTGTCCAGGATGGTTGTTGTGATGGTTGGCTCTTTCGTGCTTTGCTATGCCCCTTATGCCGTCACCGCCATGTATTTTGCCAATTCCGATGAGCCAAACAAGGATTACCGCCTAGTCGCCATCCCTGCTTTCTTCTCAAAGAGCTCCTGCGTGTATAATCCCCTAATCTACGCCTTCATGAACAAACAGTTCAACGCCTGCATCATGGAGACTGTATTT---------------------------------------------------------------------GGCAAGAAG---------ATTGATGAGTCCTCA------------GAGGTTTCC---------AGCAAG---ACCGAA---------------------------------------------------ACCTCATCTGTGTCTGCATAA---------------------------------------------------------------------------------------

>KX768583_Mbifurca_LWS_3

ATGGCAGAGGAATGGGGAAAACAGGTGTTTGCTGCC---AGGCGGCATGAAGATACAACAAGA---GGCTCTGCATTCACATACACAAACAGCAATCATACAAAA---------------GATCCCTTTGAA------GGACCAAACTACCACATCGCTCCTCGATGGGTTTACAACCTCTCCACACTTTGGATGTGTATCGTGGTCGTTTTATCAGTCTTCACCAATGGCCTCGTTTTGGTGGCCACAGCAAAGTTCAAGAAACTTCGTCATCCTCTCAACTGGATCTTGGTCAACCTTGCCATCGCTGATCTTGGAGAGACAGTCTTTGCCAGTACCATCAGTGTGTGCAACCAGTTCTTTGGATATTTCATTCTGGGACACCCAATGTGTGTCTTTGAAGGCTTTACTGTCTCAACTTGTGGTATTGCTGCTCTATGGTCCCTGACCATCATCTCTTGGGAGAGATGGGTAGTTGTTTGCAAACCTTTTGGAAATGTCAAGTTTGATGAA---------AAGTGGGCCACGGCTGGAATAGTTTTCTCCTGGGTCTGGTCTGCAGCGTGGTGCGCTCCTCCCATCTTTGGATGGAGCAGGTTTTGGCCTCATGGACTGAAAACGTCCTGCGGACCTGATGTGTTCAGTGGAAGCGACGACCCAGGGGTCCTGTCCTACATGATTGTCCTCATGATTACTTGTTGCTTTATTCCTCTGGCTATCATCATCTTGTGCTACCTGGCTGTGTGGTTGGCCATCCGTGCTGTTGCTATGCAGCAGAAGGAATCCGAGTCGACCCAGAAGGCTGAGAGAGAAGTGTCCAGGATGGTTGTAGTCATGATCGTAGCTTACTGTGTCTGCTGGGGACCCTACACCTTCTTCGCCTGCTTTGCCGCAGCCAACCCCGGATACGCCTTCCATCCTTTGGCCGCTGCCATGCCTGCATACTTTGCCAAAAGTGCCACCATCTACAACCCTATTATCTATGTCTTCATGAACCGACAGTTCCGCACATGCATCATGCAGCTC---TTT---------------------------------------------------------------------GGCAAACAG---------GTGGATGATGGTTCT------------GAAGTGTCCACA------TCAAAG---ACAGAG---------------------------------------------------GTCTCCTCTGTGGCTCCTGCATAA------------------------------------------------------------------------------------

>KX768605_Mbifurca_LWS_1

ATGGCAGAGGAATGGGGAAAACAGGTGTTTGCTGCC---AGGCGGCACGAAGATACTACAAGA---GGCGCCGCATTCACATACACAAACAGCAATCATACAAAA---------------GATCCCTTTGAA------GGACCAAACTACCACATCGCTCCTCGATGGGTTTACAACCTCTCCACACTTTGGATGTGTATCGTGGTCGTTTTATCAGTCTTCACCAACGGCCTCGTTTTGGTGGCCACAGCAAAGTTCAAGAAACTTCGTCATCCTCTCAACTGGATCTTGGTCAACCTTGCCATCGCCGATCTTGGAGAGACAGTCTTTGCCAGTACCATCAGTGTGTGCAACCAGTTCTTTGGATATTTCATTCTGGGACACCCAATGTGTGTCTTTGAAGGGTATACAGTCTCAACTTGTGGTATTGCTGCTCTGTGGTCCCTGACCATCATTTCTTGGGAGAGATGGGTAGTTGTGTGCAAACCATTTGGAAATGTCAAGTTTGATGAA---------AAGTGGGCCACGGCTGGAATAGTTTTCTCCTGGGTCTGGGCTGCAGCGTGGTGCGCTCCTCCCATCTTTGGATGGAGCAGGTATTGGCCTCATGGACTGAAAACGTCCTGCGGACCTGATGTGTTCAGTGGAAGCGATGACCCAGGGGTCCTGTCCTACATGATTGTCCTCATGATTACATGCTGCATCATTCCTCTGGCTATCATCATCTTGTGCTACCTGGCTGTGTGGTTGGCCATCCGTGCTGTTGCTATGCAGCAGAAGGAATCCGAGTCGACCCAGAAGGCTGAGAGAGAAGTGTCCAGGATGGTTGTAGTCATGATCATAGCTTACTGTGTCTGCTGGGGACCCTACACCTTCTTCGCCTGCTTTGCCGCAGCCAACCCCGGATACGCCTTCCATCCTTTGGCCGCTGCCATGCCTGCATACTTTGCCAAAAGTGCCACCATCTACAACCCTGTTATCTATGTCTTCATGAACCGACAGTTCCGCACATGCATCATGCAGCTC---TTT---------------------------------------------------------------------GGCAAACAG---------GTGGATGATGGTTCT------------GAAGTGTCCACA------TCAAAG---ACAGAG---------------------------------------------------GTCTCCTCTGTGGCTCCTGCATAA------------------------------------------------------------------------------------

>MN817663_Gaffinis_LWS_3

------------------------------------------CGGCACGAAGATACAACAAGA---GGCTCCGCATTCACATACACAAACAGCAATCATACCAAA---------------GATCCTTTTGAA------GGACCAAACTACCACATCGCTCCTCGATGGGTTTACAACATCTCCACACTCTGGATGTGTATCGTGGTCGTTTTATCAGTCTTCACCAACGGCCTCGTCTTGGTGGCCACAGCAAAATTCAAGAAACTTCGTCATCCTCTCAACTGGATCTTGGTCAACCTTGCCATTGCTGATCTTGGAGAGACAGTCTTTGCCAGCACCATCAGTGTGTGCAACCAGTACTTTGGATATTTCATTCTGGGACACCCAATGTGTGTCTTTGAAGGCTATACTGTCTCAACTTGTGGTATTGCTGCTCTTTGGTCCCTGACTATCATCTCTTGGGAGAGATGGATAGTTGTGTGCAAACCCTTTGGAAATGTCAAGTTCGATGCC---------AAGTGGGCCACAGGTGGAATAGTTTTCTCCTGGGTCTGGTCTGCAGCGTGGTGTGCTCCTCCCATCTTTGGATGGAGCAGGTATTGGCCTCATGGACTGAAAACGTCCTGCGGGCCTGATGTGTTCAGTGGAAGCGAAGACCCTGGAGTCCAGTCCTACATGGTTGTCCTCATGATTACATGCTGCATCATTCCTCTGGCTATCATCATCTTGTGCTACCTGGCTGTGTGGTTGGCCATCCGTGCTGTTGCTATGCAGCAGAAGGAATCTGAGTCGACCCAGAAGGCTGAGAGAGAAGTGTCCAGGATGGTTGTAGTCATGATCATAGCTTACTGTGTCTGCTGGGGACCCTACACCTTTTTCGCCTGCTTTGCCGCAGCCAACCCCGGATACGCCTTCCATCCTTTGGCTGCTGCCATGCCTGCATACTTTGCCAAAAGCGCCACCATCTACAACCCTGTTATCTATGTCTTCATGAACCGA---------------------------------------------------------------------------------------------------------------------------------------------------------------------------------------------------------------------------------------------------------------------------------------------------------------------------------------------------

>KX768600_Pvelifera_LWS_1

ATGGCAGAGGAATGGGGAAAACAGGTGTTTGCTGCC---AGGCGGCACGAAGATACAACAAGA---GGCGCCGCATTCACATACACAAACAGCAATCATACAAAA---------------GATCCCTTTGAA------GGACCAAACTACCACATCGCTCCTCGATGGGTTTACAATATCTCCACACTCTGGATGTGTATTGTGGTCGTTTTATCAGTCTTCACCAATGGCCTCGTCTTGGTGGCCACAGCAAAGTTCAAGAAACTTCGTCATCCTCTCAACTGGATCTTGGTCAACCTTGCTATTGCTGATCTTGGAGAGACAGTCTTTGCCAGCACCATCAGTGTGTGCAACCAGTTCTTTGGATATTTCATTCTGGGACATCCAATGTGTGTCTTTGAAGGCTTTACTGTCTCAACTTGTGGTATTACTGCTCTGTGGTCCCTGACCATCATCTCTTGGGAGAGATGGATAGTTGTGTGCAAACCTTTTGGAAATGTCAAGTTCGATGCC---------AAGTGGGCCACAGGTGGAATAGTTTTCTCCTGGGTCTGGTCTGCAGCGTGGTGCGCTCCTCCCATCTTTGGATGGAGCAGGTATTGGCCTCATGGACTGAAAACGTCTTGCGGACCTGATGTGTTCAGTGGAAGCGATGACCCAGGGGTCCTGTCCTACATGATTGTCCTCATGATTACATGCTGCATCATTCCTCTGGCTATCATCATCTTGTGCTACCTGGCTGTGTGGTTGGCCATCCGTGCTGTTGCTATGCAGCAGAAGGAATCTGAGTCGACCCAGAAGGCTGAGAGAGAAGTGTCCAGGATGGTTGTAGTCATGATCGTAGCTTACTGTGTCTGCTGGGGACCCTACACTTTCTTCGCCTGCTTTGCCGCAGCCAACCCCGGATACGCCTTCCATCCTTTGGCCGCTGCCATGCCTGCATACTTTGCCAAAAGCGCCACCATCTACAACCCTGTTATCTATGTCTTCATGAACCGACAGTTCCGCACATGTATCATGCAGCTC---TTT---------------------------------------------------------------------GGCAAACAG---------GTGGATGATGGTTCT------------GAAGTGTCCACA------TCAAAG---ACAGAG---------------------------------------------------GTCTCCTCTGTGGCTCCTGCATAA------------------------------------------------------------------------------------

>KX768578_Pvelifera_LWS_3

ATGGCAGAGGAATGGGGAAAACAGGTGTTTGCTGCC---AGGCGGCACGAAGATACAACAAGA---GGCGCCGCATTCACATACACAAACAGCAATCATACAAAA---------------GATCCCTTTGAA------GGACCAAACTACCACATCGCTCCTCGATGGGTTTACAATATCTCCACACTCTGGATGTGTATTGTGGTCGTTTTATCAGTCTTCACCAATGGCCTCGTCTTGGTGGCCACAGCAAAGTTCAAGAAACTTCGTCATCCTCTCAACTGGATCTTGGTCAACCTTGCTATTGCTGATCTTGGAGAGACAGTCTTTGCCAGCACCATCAGTGTGTGCAACCAGTTCTTTGGATATTTCATTCTGGGACATCCAATGTGTGTCTTTGAAGGCTTTACTGTCTCAACTTGTGGTATTACTGCTCTGTGGTCCCTGACCATCATCTCTTGGGAGAGATGGATAGTTGTGTGCAAACCTTTTGGAAATGTCAAGTTCGATGCC---------AAGTGGGCCACAGGTGGAATAGTTTTCTCCTGGGTCTGGTCTGCAGCATGGTGCGCTCCTCCCATCTTTGGATGGAGCAGGTATTGGCCTCATGGACTGAAAACGTCTTGCGGACCTGATGTGTTCAGTGGAAGCGATGACCCAGGGGTCCTGTCCTACATGATTGTCCTCATGATTACATGCTGCATCATTCCTCTGGCTATCATCATCTTGTGCTACCTGGCTGTGTGGTTGGCCATCCGTGCTGTTGCTATGCAGCAGAAGGAATCTGAGTCGACCCAGAAGGCTGAGAGAGAAGTGTCCAGGATGGTTGTAGTCATGATCGTAGCTTACTGTGTCTGCTGGGGACCCTACACTTTCTTCGCCTGCTTTGCCGCAGCCAACCCCGGATACGCCTTCCATCCTTTGGCCGCTGCCATGCCTGCATACTTTGCCAAAAGCGCCACCATCTACAACCCTGTTATCTATGTCTTCATGAACCGACAGTTCCGCACATGCATCATGCAGCTC---TTT---------------------------------------------------------------------GGCAAACAG---------GTGGATGATGGTTCT------------GAAGTGTCCACA------TCAAAG---ACAGAG---------------------------------------------------GTGTCATCTGTGGCTCCTGCATAA------------------------------------------------------------------------------------

>KX768584_Pminor_LWS_3

ATGGTAGAGGAATGGGGAAAACAGGTGTTTGCAGCC---AGGAGGCATGAAGATACAACAAGA---GGCTCTGCATTCACATACACAAACAGCAATCATACCAAA---------------GATCCCTTTGAA------GGACCAAACTACCACATCGCTCCTCGATGGGTTTACAACATCTCCACACTCTGGATGTGTATCGTGGTCGTTTTATCAGTCTTCACCAACGGCCTCGTCTTGGTGGCCACAGCAAAGTTCAAGAAACTTCGTCATCCTCTCAACTGGATCTTGGTCAACCTTGCCGTCGCTGATCTTGGAGAGACAGTCTTTGCCAGCACCATCAGTGTGTGCAACCAGTTCTTTGGATATTTCATTCTGGGACACCCAATGTGTGTCTTTGAAGGCTTTACTGTCTCAACTTGTGGTATTGCTGCTCTGTGGTCCCTGACCATCATCTCTTGGGAGAGATGGGTAGTTGTGTGCAAACCTTTTGGAAATGTCAAGTTTGATGCC---------AAGTGGGCCACAGCTGGAATAGTTTTCTCCTGGGTCTGGTCTGCAGCGTGGTGTGCTCCTCCCGTCTTTGGATGGAGCAGGTACTGGCCTCATGGACTGAAAACGTCCTGTGGACCTGATGTGTTCAGTGGAAGTGATGACCCAGGGGTCCTGTCCTACATGATTGTCCTTATGATTACATGCTGCATCATTCCTCTAGCTATCATCATCTTGTGCTACCTGGCTGTGTGGTTGGCCATCCGTGCTGTTGCTATGCAACAGAAGGAATCTGAGTCGACCCAGAAGGCTGAGAGAGAAGTGTCCAGGATGGTTGTAGTCATGATCTTAGCTTACTGTGTCTGCTGGGGACCCTACACCTTTTTTGCCTGCTTTGCCGCAGCCAACCCCGGATACGCCTTCCATCCTTTGGCCGCTGCCATGCCTGCATACTTTGCCAAAAGCGCCACCATCTACAACCCTGTTATCTATGTCTTCATGAACCGACAGTTCCGCACATGCATCATGCAGCTC---TTT---------------------------------------------------------------------GGCAAACAG---------GGGGATGATGGTTCT------------GAAGTGTCCACA------TCAAAG---ACAGAG---------------------------------------------------GTCTCCTCTGTGGCTCCTGCATAA------------------------------------------------------------------------------------

>KX768606_Pminor_LWS_1

ATGGCAGAGGAATGGGGAAGACAGGTGTTTGCAGCC---AGGAGGCATGAAGATACAACAAGA---GGCTCTGCATTCACATACACAAACAGCAATCATACCAAA---------------GATCCCTTTGAA------GGACCAAACTACCACATCGCTCCTCGATGGGTTTACAACATCTCCACACTCTGGATGTGTATCGTGGTCGTTTTATCAGTCTTCACCAACGGCCTCGTCTTGGTGGCCACAGCAAAGTTCAAGAAACTTCGTCATCCTCTCAACTGGATCTTGGTCAACCTTGCCGTCGCTGATCTTGGAGAGACAGTCTTTGCCAGCACCATCAGTGTGTGCAACCAGTTCTTTGGATATTTCATTCTGGGACACCCAATGTGTGTCTTTGAAGGCTTTACTGTCTCAACTTGTGGTATTGCTGCTCTGTGGTCCCTGACCATCATCTCTTGGGAGAGATGGGTAGTTGTGTGCAAACCTTTTGGAAATGTCAAGTTTGATGCC---------AAGTGGGCCACAGCTGGAATAGTTTTCTCCTGGGTCTGGTCTGCAGCGTGGTGCGCTCCTCCCATCTTTGGATGGAGCAGGTACTGGCCTCATGGACTGAAAACGTCCTGTGGACCTGATGTGTTCAGTGGAAGTGATGACCCAGGGGTCCTGTCCTACATGATTGTCCTTATGATTACATGCTGCATCATTCCTCTAGCTATCATCATCTTGTGCTACCTGGCTGTGTGGTTGGCCATCCGTGCTGTTGCTATGCAACAGAAGGAATCTGAGTCGACCCAGAAGGCTGAGAGAGAAGTGTCCAGGATGGTTGTAGTCATGATCTTAGCTTACTGTGTCTGCTGGGGACCCTACACCTTTTTTGCCTGCTTTGCCGCAGCCAACCCCGGATACGCCCTTCATCCTTTGGCCGCTGCCATGCCTGCATACTTTGCCCAAAGCGCCCCCATCTACAACCCTGTTATCTATGTTTTCATGAACCGACAGTTCCGCACATGCATCATGCAGCTC---TTT---------------------------------------------------------------------GGCAAACAG---------GTGGATGATGGTTCT------------GAAGTGTCCACA------TCAAAG---ACAGAG---------------------------------------------------GTCTCCTCTGTGGCTCCTGCATAA------------------------------------------------------------------------------------

>HQ260679_Preticulata_LWS_1

------------------------------------------------------------------------------------------------------------------------------------------------------------------------GTTTACAACGTCTCCACACTCTGGATGTGTATCGTGGTCGTTTTATCAGTCTTCACCAACGGCCTCGTCTTGGTGGCCACAGCAAAGTTCAAGAAACTTCGTCATCCTCTCAACTGGATCTTGGTCAACCTTGCCATTGCTGATCTTGGAGAGACAGTCTTTGCCAGCACCATCAGTGTGTGCAACCAGTTCTTTGGATATTTCATTCTGGGACACCCAATGTGTGTCTTTGAAGGCTATGTTGTCTCAACTTGTGGTATTGCTGCTCTGTGGTCCCTGACTATTATCTCTTGGGAGAGATGGATAGTTGTGTGCAAACCTTTTGGAAATGTCAAGTTTGATGCC---------AAGTGGGCCACAGCTGGAATAGTTTTCTCCTGGGTCTGGGCTGCAGTGTGGTGCGCTCCTCCCATCTTTGGATGGAGCAGGTATTGGCCTCATGGACTGAAAACGTCCTGCGGACCTGATGTGTTCAGTGGAAGCGATGACCCAGGGGTCCTGTCCTACATGATTGTCCTCATGATTACATGCTGCATCATTCCTCTGGCTATCATCATCTTGTGCTACCTGGCTGTGTGGTTGGCCATCCGTGCTGTTGCTATGCAGCAGAAGGAATCTGAGTCGACCCAGAAGGCTGAGAGAGAAGTGTCCAGGATGGTTGTAGTCATGATCATAGCTTACTGTGTCTGCTGGGGACCCTACACCTTCTTCGCCTGCTTTGCCGCAGCCAACCCTGGATACGCCTTCCATCCTTTGGCCGCTGCCATGCCTGCATACTTTGCCAAAAGCGCCACCATCTACAACCCTGTTATCTATGTCTTCATGAACCGACAGTTCCGCACATGCATCATGCAGCTC---TTT---------------------------------------------------------------------GGCAAACAG---------GTGGATGATGGTTCT------------GAAGTGTCCACA------TCAAAG---ACAGAG---------------------------------------------------GTCTCCTCTGTGGCTCCTGCATAA------------------------------------------------------------------------------------

>KX768610_Hformosa_LWS_1

ATGGCAGAGGAATGGGGAAAACAGGTGTTTGCTGCC---AGGCGGCACGAAGATACAACAAGA---GGCGCCGCATTCACATATACAAACAGCAATCATACAAAA---------------GATCCTTTTGAA------GGACCAAACTACCACATCGCTCCTCGATGGGTTTACAACGTCTCCACACTCTGGATGTGTATCGTGGTTGTTTTATCAGTCTTCACCAATGGCCTCGTCTTGGTGGCCACAGCAAAGTTCAAGAAACTTCGTCATCCTCTCAACTGGATCTTGGTCAATCTTGCCATTGCTGATCTTGGAGAGACAGTCTTTGCCAGCACCATCAGTGTGTGCAACCAGTTCTTTGGATATTTCATCCTGGGACACCCAATGTGTGTCTTTGAAGGCTATGTTGTCTCAACTTGTGGTATTGCTGCTCTTTGGTCCCTGACTATCATCTCTTGGGAGAGATGGATAGTTGTGTGCAAACCCTTTGGAAATGTCAAGTTCGATGCC---------AAGTGGGCCACAGCTGGAATAGTTTTCTCCTGGGTCTGGTCTGCAGTGTGGTGTGCTCCTCCCATCTTTGGATGGAGCAGGTATTGGCCTCATGGACTGAAAACATCCTGTGGGCCTGATGTGTTCAGTGGAAGTGATGACCCAGGGGTCCTGTCCTACATGATTGTCCTTATGGTTACATGCTGCATCATTCCTCTGGCTATCATCATTCTGTGCTACCTGGCTGTGTGGTTGGCCATCCGTGCTGTTGCTATGCAGCAGAAGGAATCTGAGTCGACCCAGAAGGCTGAGAGAGAAGTGTCCAGGATGGTTGTCGTCATGATCATAGCTTACTGTGTCTGCTGGGGACCCTACACCTTTTTCGCCTGCTTTGCTGCAGCCAATCCCGGATACGCCTTCCATCCTTTGGCTGCTGCCATGCCTGCATACTTTGCCAAAAGCGCCACCATCTACAACCCTGTTATCTATGTCTTCATGAACCGACAGTTCCGCACATGCATCATGCAGCTC---TTT---------------------------------------------------------------------GGCAAACAG---------GTGGATGATGGTTCT------------GAAGTGTCCACA------TCCAAG---ACAGAA---------------------------------------------------GTCTCCTCTGTGGCTCCTGCATAA------------------------------------------------------------------------------------

>KX768588_Hformosa_LWS_3

ATGGCAGAGGAATGGGGTAAACAGGTGTTTGCTGCC---AGGCGGCACGAAGATACAACAAGA---GGCGCCGCATTCACATATACAAACAGCAATCATACAAAA---------------GATCCTTTTGAA------GGACCAAACTACCACATCGCTCCTCGATGGGTTTACAATGTCTCCACACTCTGGATGTGTATCGTGGTTGTTTTATCAGTCTTCACCAACGGCCTCGTCTTGGTGGCCACAGCAAAGTTCAAGAAACTTCGTCATCCTCTCAACTGGATCTTGGTCAATCTTGCCATTGCTGATCTTGGAGAGACAGTCTTTGCCAGCACCATCAGTGTGTGCAACCAGTTCTTTGGATATTTCATCCTGGGACACCCAATGTGTGTCTTTGAAGGCTATGTTGTCTCAACTTGTGGTATTGCTGCTCTTTGGTCCCTGACTATCATCTCTTGGGAGAGATGGATAGTTGTGTGCAAACCCTTTGGAAATGTCAAGTTCGATGCC---------AAGTGGGCCACAGCTGGAATAGTTTTCTCCTGGGTCTGGTCTGCAGTGTGGTGTGCTCCTCCCATCTTTGGATGGAGCAGGTATTGGCCTCATGGACTGAAAACATCCTGTGGGCCTGATGTGTTCAGTGGAAGTGATGACCCAGGGGTCCTGTCCTACATGATTGTCCTTATGGTTACATGCTGCATCATTCCTCTGGCTATCATCATTCTGTGCTACCTGGCTGTGTGGTTGGCCATCCGTGCTGTTGCTATGCAGCAGAAGGAATCTGAGTCGACCCAGAAGGCTGAGAGAGAAGTGTCCAGGATGGTTGTCGTCATGATCATAGCTTACTGTGTCTGCTGGGGACCCTACACCTTTTTCGCCTGCTTTGCTGCAGCCAATCCCGGATACGCCTTCCATCCTTTGGCTGCTGCCATGCCTGCATACTTTGCCAAAAGCGCCACCATCTACAACCCTGTTATCTATGTCTTCATGAACCGACAGTTCCGCACATGCATCATGCAGCTC---TTT---------------------------------------------------------------------GGCAAACAG---------GTGGATGATGGTTCT------------GAAATGTCCACA------TCCAAG---ACAGAA---------------------------------------------------GTCTCCTCTGTGGCTCCTGCATAA------------------------------------------------------------------------------------

>KX768609_Pcaymanensis_LWS_1

ATGGCAGAGGAATGGGGAAAACAGGTGTTTGCTGCC---AGGCGGCATGAAGATACAACAAGA---GGCGCCGCATTCACATACACTAACAGCAATCATACAAAA---------------GATCCCTTTGAA------GGACCAAACTACCACATTGCTCCTCGATGGGTTTACAACGTCTCCACACTCTGGATGTTTATCGTGGTTGTTTTATCAGTCTTCACCAACGGCCTCGTCTTGGTGGCCACAGCAAAGTTCAAGAAACTTCGTCATCCTCTCAACTGGATCTTAGTCAACCTTGCCATTGCTGATCTTGGAGAGACAGTCTTTGCCAGCACTATCAGTGTGTGCAACCAGTTCTTTGGATATTTCATTCTGGGACACCCAATGTGTGTCTTTGAAGGCTATGTTGTCTCAACTTGTGGTATTGCTGCTCTGTGGTCCCTGACCATCATCTCTTGGGAGAGATGGATAGTTGTGTGCAAACCTTTTGGAAATGTCAAGTTTGATGCC---------AAGTGGGCCACAGGTGGAATAGTTTTCTCCTGGGTCTGGTCTGCAGTGTGGTGCGCTCCTCCCATCTTTGGATGGAGCAGGTACTGGCCTCATGGACTGAAAACGTCTTGCGGACCTGATGTGTTCAGTGGAAGCGATGACCCAGGGGTCCTGTCCTACATGATTGTGCTCATGATTACATGCTGCATCATTCCTCTGGCTATCATCATCTTGTGCTACCTGGCTGTGTGGTTGGCCATCCGTGCTGTTGCTATGCAGCAGAAGGAATCTGAGTCGACCCAGAAGGCTGAGAGAGAAGTGTCCAGGATGGTTGTAGTCATGATCATAGCTTACTGTGTCTGCTGGGGACCCTACACCTTCTTCGCCTGTTTTGCCGCAGCCAACCCCGGATACGCCTTCCATCCTTTGGCCGCTGCCATGCCTGCATACTTTGCCAAAAGCGCCACCATCTACAACCCTGTTATCTATGTCTTCATGAACCGACAGTTCCGCACATGCATCATGCAGCTC---TTT---------------------------------------------------------------------GGCAAACAG---------GTGGATGATGGTTCT------------GAAGTGTCCACA------TCAAAG---ACAGAG---------------------------------------------------GTCTCCTCTGTGGCTCCTGCATAA------------------------------------------------------------------------------------

>KX768587_Pcaymanensis_LWS_3

ATGGCAGAGGAATGGGGAAAACAGGTGTTTGCTGCC---AGGCGGCATGAAGATACAACAAGA---GGCGCCGCATTCACATACACTAACAGCAATCATACAAAA---------------GATCCCTTTGAA------GGACCAAACTACCACATTGCTCCTCGATGGGTTTACAACGTCTCCACACTCTGGATGTTTATCGTGGTTGTTTTATCAGTCTTCACCAACGGCCTCGTCTTGGTGGCCACAGCAAAGTTCAAGAAACTTCGTCATCCTCTCAACTGGATCTTAGTCAACCTTGCCATTGCTGATCTTGGAGAGACAGTCTTTGCCAGCACTATCAGTGTGTGCAACCAGTTCTTTGGATATTTCATTCTGGGACACCCAATGTGTGTCTTTGAAGGCTATGTTGTCTCAACTTGTGGTATTGCTGCTCTGTGGTCCCTGACCATCATCTCTTGGGAGAGATGGATAGTTGTGTGCAAACCTTTTGGAAATGTCAAGTTTGATGCC---------AAGTGGGCCACAGGTGGAATAGTTTTCTCCTGGGTCTGGTCTGCAGTGTGGTGCGCTCCTCCCATCTTTGGATGGAGCAGGTACTGGCCTCATGGACTGAAAACGTCTTGCGGACCTGATGTGTTCAGTGGAAGCGATGACCCAGGGGTCCTGTCCTACATGATTGTGCTCATGATTACATGCTGCATCATTCCTCTGGCTATCATCATCTTGTGCTACCTGGCTGTGTGGTTGGCCATCCGTGCTGTTGCTATGCAGCAGAAGGAATCTGAGTCGACCCAGAAGGCTGAGAGAGAAGTGTCCAGGATGGTTGTAGTCATGATCATAGCTTACTGTGTCTGCTGGGGACCCTACACCTTCTTCGCCTGTTTTGCCGCAGCCAACCCCGGATACGCCTTCCATCCTTTGGCCGCTGCCATGCCTGCATACTTTGCCAAAAGCGCCACCATCTACAACCCTGTTATCTATGTCTTCATGAACCGACAGTTCCGCACATGCATCATGCAGCTC---TTT---------------------------------------------------------------------GGCAAACAG---------GTGGATGATGGTTCT------------GAAGTGTCCACA------TCAAAG---ACAGAG---------------------------------------------------GTCTCCTCTGTGGCTCCTGCATAA------------------------------------------------------------------------------------

>KX768608_Lnigrofasciata_LWS_1

ATGGCAGAGGAATGGGGAAAACAGGTGTTTGCTGCC---AGGCGGCATGAAGATACAACAAGA---GGCGCCGCATTCACATACACAAACAGCAATCATACAAAA---------------GATCCCTTTGAA------GGACCAAACTACCACATTGCTCCTCGATGGGTTTACAACGTCTCCACACTCTGGATGTGTATCGTGGTCGTTTTATCAGTCTTCACCAACGGCCTCGTCTTGGTGGCCACAGCAAAGTTCAAGAAACTTCGTCATCCTCTCAACTGGATCTTAGTCAACCTTGCCATTGCTGATCTTGGAGAGACAGTCTTTGCCAGCACTATCAGTGTGTGCAACCAGTTCTTTGGATATTTCATTCTGGGACACCCAATGTGTGTCTTTGAAGGCTATGTTGTCTCAACTTGTGGTATTGCTGCTCTGTGGTCCCTGACCATCATCTCTTGGGAGAGATGGATAGTTGTGTGCAAACCTTTTGGAAATGTCAAGTTTGATGCC---------AAGTGGGCCACAGGTGGAATAGTTTTCTCCTGGGTCTGGTCTGCAGTGTGGTGCGCTCCTCCCATCTTTGGATGGAGCAGGTACTGGCCTCATGGACTGAAAACGTCTTGCGGACCTGATGTGTTCAGTGGAAGCGATGACCCAGGGGTCCTATCCTACATGATTGTGCTCATGAGTACATGCTGCATCATTCCTCTGGCTATCATCATCTTGTGCTACCTGGCTGTGTGGTTGGCCATCCGTGCTGTTGCTATGCAGCAGAAAGAATCTGAGTCGACCCAGAAGGCTGAGAGAGAAGTGTCCAGGATGGTTGTAGTCATGATCATAGCTTACTGTGTCTGCTGGGGACCCTACACCTTCTTCGCCTGCTTTGCCGCAGCCAACCCCGGATACGCCTTCCATCCTTTGGCCGCTGCCATGCCTGCATACTTTGCCAAAAGCGCCACCATCTACAACCCTGTTATCTATGTCTTCATGAACCGACAGTTCCGCACATGCATCATGCAGCTC---TTT---------------------------------------------------------------------GGCAAACAG---------GTGGATGATGGTTCT------------GAAGTGTCCACA------TCAAAG---ACAGAG---------------------------------------------------GTCTCCTCTGTGGCTCCTGCATAA------------------------------------------------------------------------------------

>HQ260681_Preticulata_LWS_3

---------------------------------------------------------------------------------------------------------------------------------------------------------------------TGGGTTTACAACGTCTCCACACTCTGGATGTGTATCGTGGCCGTTTTATCAGTCTTCACCAACGGCCTCGTCTTGGTGGCCACAGCAAAGTTCAAGAAACTTCGTCATCCTCTCAACTGGATCTTGGTCAACCTTGCCATTGCTGATCTTGGAGAGACTGTCTTTGCCAGTACCATCAGTGTGTGCAACCAGTTCTTTGGATATTTCATTCTGGGACACCCAATGTGTGTCTTTGAAGGCTTTGTTGTCTCAACTTGTGGTATTGCTGCTCTATGGTCCCTGACTATCATCTCTTGGGAGAGATGGATAGTTGTGTGCAAACCTTTTGGAAATGTCAAGTTCGATGCC---------AAGTGGGCCACAGGTGGAATAGTTTTCTCCTGGGTCTGGTCTGCAGCGTGGTGCGCACCTCCCATCTTTGGATGGAGCAGGTTTTGGCCCCATGGACTGAAAACATCCTGCGGACCTGATGTGTTCAGTGGAAGCGATGACCCAGGGGTCCTGTCCTACATGATTGTCCTCATGATTACATGCTGCATCATTCCTCTGGCTATCATCATCTTGTGCTACCTGGCTGTGTGGTTGGCCATCCATGCTGTTGCTATGCAGCAGAAGGAATCTGAGTCGACCCAGAAGGCTGAGAGAGAAGTGTCCAGGATGGTTGTAGTCATGATCATAGCTTACTGTGTCTGCTGGGGACCCTACACCTTCTTCGCCTGCTTTGCCGCAGCCAACCCCGGATACGCCTTCCATCCTTTGGCCGCTGCCATGCCTGCATACTTTGCCAAAAGCGCCACCATCTACAACCCTGTTATCTATGTCTTCATGAACCGACAGTTCCGCACATGCATCATGCAGCTC---TTT---------------------------------------------------------------------GGCAAACAG---------GTGGATGATGGTTCT------------GAAGTGTCCACA------TCAAAG---ACAGAG---------------------------------------------------GTCTCCTCTGTGGCTCCTGCATAA------------------------------------------------------------------------------------

>KX768586_Lnigrofasciata_LWS_3

ATGGCAGAGGAATGGGGAAAACAGGTGTTTGCTGCC---AGGCGGCATGAAGATACAACAAGA---GGCGCCGCATTCACATACACAAACAGCAATCATACAAAA---------------GATCCCTTTGAA------GGACCAAACTACCACATTGCTCCTCGATGGGTTTACAACGTCTCCACACTCTGGATGTGTATCGTGGTCGTTTTATCAGTCTTCACCAACGGCCTCGTCTTGGTGGCCACAGCAAAGTTCAAGAAACTTCGTCATCCTCTCAACTGGATCTTAGTCAACCTTGCCATTGCTGATCTTGGAGAGACAGTCTTTGCCAGCACTATCAGTGTGTGCAACCAGTTCTTTGGATATTTCATTCTGGGACACCCAATGTGTGTCTTTGAAGGCTATGTTGTCTCAACTTGTGGTATTGCTGCTCTGTGGTCCCTGACCATCATCTCTTGGGAGAGATGGATAGTTGTGTGCAAACCTTTTGGAAATGTCAAGTTTGATGCC---------AAGTGGGCCACAGGTGGAATAGTTTTCTCCTGGGTCTGGTCTGCAGTGTGGTGCGCTCCTCCCATCTTTGGATGGAGCAGGTACTGGCCTCATGGACTGAAAACGTCTTGCGGACCTGATGTGTTCAGTGGAAATGAAGACCCAGGAGTCCTGTCCTACATGATTGTGCTCATGAGTACATGCTGCATCATTCCTCTGGCTATCATCATCTTGTGCTACCTGGCTGTGTGGTTGGCCATCCGTGCTGTTGCTATGCAGCAGAAGGAATCTGAGTCGACCCAGAAGGCTGAGAGAGAAGTGTCCAGGATGGTTGTAGTCATGATCATAGCTTACTGTGTCTGCTGGGGACCCTACACCTTCTTCGCCTGCTTTGCCGCAGCCAACCCCGGATACGCCTTCCATCCTTTGGCCGCTGCCATGCCTGCATACTTTGCCAAAAGCGCCACCATCTACAACCCTGTTATCTATGTCTTCATGAACCGACAGTTCCGCAGATGCATCATGCAGCTC---TTT---------------------------------------------------------------------GGCAAACAG---------GTGGAGGATGGTTCT------------GAAGTGTCCACA------TCAAAG---ACAGAG---------------------------------------------------GTCTCCTCTGTGGCGCCTGCATAA------------------------------------------------------------------------------------

>KX768619_Pminor_SWS2B

ATG------------------------------AAGATGAGGACAAGTCGTCAAGAGGAAACG---CCAGATGACTTCTGGATCCCCATACCCCTGGAAACCGACAACATCACAGCCCTGAGCCCGTACCTA------GTCCCCCAGGACCATTTAGGGAGCCTGTGGCTTTTTTATTCAATGTCAGCGTTAATGTTCTTCTTGTTTGTGGCCGGCACGGCAATCAATGTCCTCACAATCGCATGTACTATTCAATACAAGAAGCTCCGCTCCCATCTGAACTACATCCTGGTCAACATGGCTGTGGCGAACCTCATCGTCTCGTCCGCGGGCTCCTTTACCTGCTTCTACTGTTTTGCCTTCCGATACATGGCTCTTGGTCCTCTCGGCTGCAAGATCGAAGGATTTACGGCGGCTCTTGGTGGCATGGTGAGCCTTTGGTCTCTTGCGGTGATTGCATTTGAAAGATGGCTGGTTATCTGCAAGCCACTCGGGAACTTTGCCTTCAAGTCA---------GAGCATGCTTTGTTCTTCTGTGGACTTACCTGGGTATGTGCTTTGTGCGCTGCAGTTCCTCCGCTAGTGGGATGGAGTCGGTATATCCCTGAGGGAATGCAGTGTTCATGTGGCCCAGACTGGTACACAACGGGCAACAAGTACAACACCGAATCCTTCGTGCTGTTCCTCTTCTGCTTCTGCTTTTCCGTCCCTTTCACTTGCATTGTCTTCTGCTACTCGCAGCTGCTCTTCACACTGAAATCAGCGGCAAAGGCACAGGCAGAGTCTGCCTCCACCCAGAAGGCAGAGAAAGAGGTGACCAGGATGGTGGTTGTCATGGTGCTGGGCTTCCTGGTGTGCTACGTGCCATACGCCTCCTTTGCGCTTTGGGTCGTGAACCATCGTGGACAGACGTTTGACCTGAGACTTGCTACCATACCGTCCTGTGTCTCAAAAGCCTCCACGGTCTACAATCCTGTCATCTATGTTCTCCTCAATAAGCAGTTCCGCTCTTGCATGAGGAAGATG---CTG---------------------------------------------------------------------GGGATGAGTGGA---GGCGACGAGGAGGAGTCA------------TCTGCAAGCCAG------TCGGTC---ACCGAA---------------------------------------------------GTCTCAAAAGTTGGACCCTCTTAA------------------------------------------------------------------------------------

>KX768618_Mbifurca_SWS2B

ATG------------------------------AAGATGAGGACAAGTCGTCAAGAGGAAACT---CCAGATGACTTCTGGATTCCCATACCCCTGGAAACCGACAACATCACAGCCCTGAGCCCGTACCTA------GTCCCCCAGGACCATTTAGGGAGCCTGGGGCTTTTTTATTCAATGTCAGCGTTAATGTTTGTCTTGTTTGTGGCCGGCACAGCCATCAATGTCCTCACAATCGCATGTACTATTCAATACAAGAAGCTCCGCTCCCATCTGAACTACATCCTGGTCAACATGGCTGTGGCGAACCTCATCGTCTCGTCCGTGGGCTCTTTTACCTGCTTCTACTGTTTTGCCTTCCGATACATGGCTCTTGGTCCTCTCGGCTGCAAGATCGAAGGATTTACGGCGTCTCTTGGTGGCATGGTGAGCCTTTGGTCTCTTGCAGTGATTGCATTTGAAAGATGGCTGGTTATCTGCAAGCCACTTGGGAACTTTGCCTTCAAGTCA---------GAGCATGCTTTGTTCTTCTGTGCACTTACCTGGTTCTTTGCTTTGTGCGCTGCAGTTCCTCCACTAGTGGGATGGAGTAGGTATATCCCTGAGGGAATGCAGTGTTCATGTGGACCAGACTGGTACACAACGGGCAACAAGTACAACACTGAATCCTTCGTGCTGTTCCTCTTCTGCTTCTGTTTTTCCGTCCCTTTCTCTTGCATCGTCTTCTGCTACTCGCAGCTGCTCTTCACACTGAAATCAGCAGCAAAGGCCCAGGCAGAGTCGGCCTCCACCCAGAAGGCAGAGAAAGAGGTGACCAGGATGGTGGTTGTCATGGTGCTGGGCTTCCTGGTGTGCTACGTGCCATACGCCTCCTTTGCGCTTTGGGTCGTGAACCATCGTGGACAGACATTTGACCTAAGACTTGCTACCATACCGTCCTGTGTCTCAAAAGCCTCCACGGTCTACAATCCTGTCATCTACGTTCTCCTCAATAAGCAGTTCCGCTCTTGCATGAGGAAGATG---CTG---------------------------------------------------------------------GGGATGAGTGGA---GGCGACGAGGAGGAGTCA------------TCTGCAAGTCAG------TCGGTC---ACCGAA---------------------------------------------------GTCTCAAAAGTTGGACCCTCTTAA------------------------------------------------------------------------------------

>HQ391991_Preticulata_SWS2B

------------------------------------------------------------------------------------------------------------------------------------------------------------GGGAGCCTGGGGCTTTTTTATTCAATGTCAGCGTTAATGTTCTTCTTATTTGTGGCTGGCACGGCCATCAATGTCCTCACAATCGCATGTACTATTCAATACAAGAAGCTTCGCTCCCATCTGAACTACATCCTGGTCAACATGGCTGTGGCAAACCTCATCGTCTCGTCCGTGGGCTCTTTTACCTGCTTCTACTGTTTTGCCTTCCGATACATGGCTCTTGGTCCTCTCGGATGCAAGATCGAAGGATTTACGGCGTCTCTAGGTGGCATGGTGAGCCTTTGGTCTCTTGCGGTGATTGCATTTGAAAGATGGCTGGTTATCTGCAAGCCACTCGGGAACTTTGCCTTCAAGTCA---------GAGCATGCTTTGTTCTTCTGTGCACTTACCTGGTTCTTTGCTTTGTGCGCTGCAGTTCCTCCACTAGTGGGATGGAGTAGATATATCCCTGAGGGAATGCAGTGTTCATGTGGACCAGATTGGTACACAACGGGCAACAAGTACAACACCGAATCCTTTGTGCTGTTCCTCTTCTGCTTCTGCTTTTCCGTCCCTTTCACTTGCATTGTCTTCTGCTACTCGCAGCTGCTATTCACACTGAAATCAGCAGCAAAGGCCCAGGCAGAGTCTGCCTCCACCCAGAAGGCAGAGAAAGAGGTGACCAGGATGGTGGTCGTCATGGTGCTGGGCTTCCTGGTGTGCTACATGCCATACGCCTCCTTTGCGCTTTGGGTCGTGAATCATCGTGGACAGACGTTTGATCTAAGACTTGCTACCATACCGTCCTGTGTCTCAAAAGCCTCC------------------------------------------------------------------------------------------------------------------------------------------------------------------------------------------------------------------------------------------------------------------------------------------------------------------------------------------------------------------------------------------

>MN817661_Gaffinis_SWS2B

ATG------------------------------AAGATGAGGACAAGTCGTCAAGAGGAAACT---CCAGATGACTTCTGGATCCCAATCCCCCTGGAAACCGACAACATCACAGCCCTCAGCCCGTACCTA------GTCCCCCAGGACCATTTAGGGAGCCTGGGGCTTTTTTATTCAATGTCAGCATTAATGTTCTTCTTGTTTGTGGCCGGCACAGCCATCAATGTCCTCACAATCGCATGTACTATTCAATACAAGAAGCTCCGCTCCCATCTGAACTACATCCTGGTCAACATGGCTGTGGCGAACCTCATCGTCTCGTCCGTGGGCTCTTTTACCTGCTTCTACTGTTTTGCCTTCCGATACATGGCTCTTGGTCCTCTCGGCTGCAAGATCGAAGGATTTACGGCGACTATTGGTGGCATGGTCAGCCTTTGGTCTCTTGCGGTGATTGCATTTGAAAGATGGCTGGTTATCTGCAAGCCACTCGGGAACTTTGCCTTCAAGTCA---------GAGCATGCTTTGTTCTTCTGTGCACTTACCTGGTTCTTTGCTTTGTGCGCTGCAGTTCCTCCACTAGTGGGATGGAGTAGGTATATCCCTGAGGGAATGCAGTGTTCATGTGGACCAGACTGGTACACGACGGGCAACAAGTACAACAACGAATCATTTGTGCTGTTCCTCTTCTGCTTCTGCTTTTCCGTCCCTTTCACTTGCATCGTCTTCTGCTACTCGCAGCTGCTCTTCACACTGAAATCAGCGGCAAAGGCCCAGGCAGAGTCTGCCTCCACCCAGAAGGCAGAGAAAGAGGTGACCAGGATGGTGGTCGTCATGGTGCTGGGCTTCCTGGTGTGCTACCTGCCATACGCCTCCTTTGCGCTTTGGGTCATAAACCATCGTGGACAGACGTTTGACCTGAGACTTGCTACCATACCGTCCTGTGTCTCAAAAGCCTCCACGGTCTACAATCCTGTCATCTACGTTCTCCTCAATAAGCAGTTCCGCTCTTGCATGAGGAAGACG---CTG---------------------------------------------------------------------GGGATGAGTGGA---GGCGACGAGGAGGAGTCG------------TCTGCAACTCAG------TCGGTC---ACCGAA---------------------------------------------------GTCTCAAAAGTTGGACCCTCTTAA------------------------------------------------------------------------------------

>KX768611_Pvelifera_SWS2B

ATG------------------------------AAGATGAGGACAAGTCGTCAAGAGGAAATG---CCAGATGACTTCTGGATCCCCATCCCCCTGGAAACCGACAACATCACAGCCCTGAGCCCGTACCTA------GTCCCCCAGGACCATTTAGGGAGCCTGGGGCTTTTTTATTCAATGTCAGCATTAATGTTCTTCTTGTTTGTGGCTGGCACGGCCATCAATATCCTCACAATCGCATGTACTATTCAATACAAGAAGCTCCGCTCCCATCTGAACTACATCCTGGTCAACATGGCTGTGGCGAACCTCATCGTCTCGTCCGTGGGCTCTTTTACCTGCTTCTACTGTTTTGCCTTCCGATACATGGCTCTTGGTCCTCTCGGCTGCAAGATCGAAGGATTTACGGCGTCTCTTGGTGGCATGGTGAGCCTTTGGTCTCTTGCGGTGATTGCATTTGAAAGATGGCTGGTTATCTGCAAGCCACTCGGGAACTTTGCCTTCAAGTCG---------GAGCATGCTTTGCTCTTCTGTGCACTTACCTGGTTCTGTGCTTTGTGCGCTGCAGTTCCTCCATTAGTGGGATGGAGTCGGTATATTCCTGAGGGAATGCAGTGTTCATGTGGACCAGACTGGTACACAACGGGCAATAAGTACAACACCGAAACCTTCGTGCTGTTCCTCTTCTGCTTCTGCTTTTCCGTCCCTTTCACTTGCATTGTCTTCTGCTACTCGCAGCTGCTCTTCACACTGAAATCAGCGGCAAAGGCCCAGGCAGAGTCTGCCTCCACCCAGAAGGCAGAGAAAGAGGTGACCAGGATGGTGGTCGTCATGGTGCTGGGCTTCCTGGTGTGCTACATGCCATACGCCTCCTTTGCGCTTTGGGTCGTGAACCATCGTGGACAGACGTTTGACCTGAGACTTGCTACCATACCGTCCTGTGTCTCAAAAGCCTCCACGGTCTACAATCCTGTCATCTACGTTCTCCTCAATAAGCAGTTCCGCTCTTGCATGAGGAAGATG---CTG---------------------------------------------------------------------GGGATGAGTGGA---GGCGACGAGGAGGAGTCA------------TCTGCAAGCCAG------TCGGTC---ACCGAA---------------------------------------------------GTCTCAAAAGTTGGACCCTCTTAA------------------------------------------------------------------------------------

>KX768621_Lnigrofasciata_SWS2B

ATG------------------------------AAGATGAGGACAAGTCGTCAAGAGGAAATG---CCAGATGACTTCTGGATCCCCATCCCCCTGGAAACCGACAACATCACAGCCCTGAGCCCGTACCTA------GTCCCCCAGGACCATTTAGGGAGCCTGGGGCTTTTTTATTCAATGTCAGCGTTAATGTTCTTCTTGTTTGTGGCCGGCACGGCCATCAATATCCTTACAATCGCATGTACTATTCAATACAAGAAGCTCCGCTCCCATCTGAACTACATCCTGGTCAACATGGCTGTGGCGAACCTCATCGTCTCGTCCGTGGGCTCTTTTACCTGCTTCTACTGTTTTGCCTTCCGATACATGGCTCTTGGTCCTCTCGGCTGCAAGATTGAAGGATTTACGGCGTCTCTTGGTGGCATGGTGAGCCTTTGGTCTCTTGCGGTGATTGCATTTGAAAGATGGCTGGTTATCTGCAAGCCACTGGGGAACTTTGCCTTCAAGTCA---------GAGCATGCTTTGTTCTTCTGTGCACTTACCTGGTTCTGTGCTTTGTGCGCTGCAGTTCCTCCATTAGTGGGATGGAGTCGGTATATCCCTGAGGGAATGCAGTGTTCATGTGGACCAGACTGGTACACAACGGGCAACAAGTACAACACCGAATCCTTTGTGCTGTTCCTCTTCTGCTTCTGCTTTTCCGTCCCTTTCACTTGCATCGTCTTCTGTTACTCGCAGCTGCTCTTCACACTGAAATCAGCGGCAAAGGCCCAGGCAGAGTCTGCCTCCACCCAGAAGGCAGAGAAAGAGGTGACCAGGATGGTGGTCGTCATGGTGCTGGGCTTCTTGGTGTGCTACATGCCATACGCCTCCTTTGCGCTTTGGGTCGTGAACCATCGTGGACAGACGTTTGACCTGAGACTTGCTACCATACCATCCTGTGTCTCAAAAGCCTCCACGGTCTACAATCCTGTCATCTACGTTCTCCTCAATAAGCAGTTCCGCTCTTGCATGAGGAAGATG---CTG---------------------------------------------------------------------GGGATGAGTGGA---GGCGACGAGGAGGAGTCA------------TCTGCAAGCCAG------TCGGTC---ACCGAA---------------------------------------------------GTCTCAAAAGTTGGACCCTCTTAA------------------------------------------------------------------------------------

>KX768622_Pcaymanensis_SWS2B

ATG------------------------------AAGATGAGGACAAGTCGTCAAGAGGAAATG---CCAGATGACTTCTGGATCCCCATCCCCCTGGAAACCGACAACATCACAGCCCTGAGCCCGTACCTA------GTCCCCCAGGACCATTTAGGGAGCCTGGGGCTTTTTTATTCAATGTCAGCGTTAATGTTCTTCTTGTTTGTGGCCGGCACGGCCATTAATATCCTTACAATCGCATGTACTATTCAATACAAGAAGCTCCGCTCCCATCTGAACTACATCCTGGTCAACATGGCTGTGGCGAACCTCATCGTCTCGTCCGTGGGCTCTTTTACCTGCTTCTACTGTTTTGCCTTCCGATACATGGCTCTTGGTCCTCTCGGCTGCAAGATCGAAGGATTTACGGCGTCTCTTGGTGGCATGGTGAGCCTTTGGTCTCTTGCGGTGATTGCATTTGAAAGATGGCTGGTTATCTGCAAGCCACTGGGGAACTTTGCCTTCAAGTCA---------GAGCATGCTTTGTTCTTCTGTGCACTTACCTGGTTCTGTGCTTTGTGCGCTGCAGTTCCTCCATTAGTGGGATGGAGTCGGTATATCCCTGAGGGAATGCAGTGTTCATGTGGACCAGACTGGTACACAACGGGCAACAAGTACAACACCGAATCCTTTGTGCTGTTCCTCTTCTGCTTCTGCTTTTCCGTCCCTTTCACTTGCATCGTCTTCTGCTACTCGCAGCTGCTCTTCACACTGAAATCAGCGGCAAAGGCCCAGGCAGAGTCTGCCTCCACCCAGAAGGCAGAGAAAGAGGTGACCAGGATGGTGGTCGTCATGGTGCTGGGCTTCTTGGTGTGCTACATGCCATACGCCTCCTTTGCGCTTTGGGTCGTGAACCATCGTGGACAGACGTTTGACCTGAGACTTGCTACCATACCATCCTGTGTCTCAAAAGCCTCCACGGTCTACAATCCTGTCATCTACGTTCTCCTCAATAAGCAGTTCCGCTCTTGCATGAGGAAGATG---CTG---------------------------------------------------------------------GGGATGAGTGGA---GGCGACGAGGAGGAGTCA------------TCTGCAAGCCAG------TCGGTC---ACCGAA---------------------------------------------------GTCTCAAAAGTTGGACCCTCTTAA------------------------------------------------------------------------------------

>MN817659_Gaffinis_RH2_1

---------------------------------------------------------------------AAAAACTTCTACATCCCGATGTCCAACAAAACTGGCATCGTCAGA------AGTCCCTTTGAA------TACCCGCAATACTACATGGTGGACCCAATGATCTACAAGCTCCTAGCCTTCTACATGTTCTTCCTGATCTGCACTGGAACTCCTATCAATGGCCTGACGCTGTTTGTAACTGCTCAGAACAAAAAGCTGCGCCAGCCTCTCAACTACATCCTGGTGAACTTGGCTGTGGCCGGACTCATCATGTGCGCCTTTGGTTTCACCATCACCATCACATCTGCTCTTAACGGTTACTTCATTCTTGGACCCACCTTCTGTGCCATTGAGGGATTCATGGCAACACTTGGAGGTGAAGTTGCTCTCTGGTCTCTGGTTGTTCTGGCTATTGAGAGATATATTGTTGTGTGCAAACCTATGGGAAGCTTCAAATTCACTGGA---------ACTCATGCGGGTGCTGGAGTTCTTTCCACATGGATCATGGCTCTTGCTTGTGCAGCACCTCCACTTTTCGGATGGTCAAGGTACCTTCCAGAAGGCATGCAGTGCTCCTGTGGACCTGACTACTACACCCTGGCTCCAGGATTCAACAATGAATCTTATGTCATCTACATGTTTGTTGTTCATTTCTTCATTCCTGTTTTCCTGATTTTTTTCACCTATGGAAGTCTTGTCATGACAGTCAAAGCTGCCGCAGCTCAGCAGCAGGACTCGGCTTCTACACAGAAAGCTGAGAAAGAAGTAACACGCATGTGTGTCTTGATGGTGTTGGGCTTCTTGGTAGCTTGGACGCCATATGCAACCTTCGCTGGCTGGATCTTTCTGAATAAAGGAGCAGCTTTCACGGCTCTGACTGCAGCCCTGCCTGCTTTCTTTGCAAAGAGCTCAGCCCTTTACAATCCTGTTATCTATGTCCTGTTTAACAAACAGTTCCGTAACTGCATGCTCAGCACT---ATT---------------------------------------------------------------------GGAATGGGCGGC---ATGGTGGAGGACGAAACC------------TCAGTTTCAACG------AGCAAG---ACA------------------------------------------------------------------------------------------------------------------------------------------------------------------

>GU454732_Xhellerii_RH2_1

------------------------------------------------------------------------------------------------------------------------------------------------------------------CCAATGATCTACAAGCTCCTAGCCTTTTACATGTTCTTCCTGATCTGCACTGGAACTCCCATCAATGGCCTGACGCTGTTTGTAACTGCTCAGAACAAAAAGCTGCGCCAGCCTCTCAACTACATCCTGGTGAACTTGGCTGTGGCCGGACTCATCATGTGCGCCTTCGGTTTCACCATCACCATCACATCTGCTCTTAACGGTTACTTCATTCTTGGACCCACCTTCTGTGCTATTGAGGGATTCATGGCAACACTCGGAGGTGAAGTTGCTCTCTGGTCTCTGGTTGTTCTGGCTATTGAGAGATATATTGTAGTGTGCAAACCTATGGGCAGCTTCAAATTCACTGGA---------ACTCATGCTGCTGCTGGAGTTATTTCCACATGGATCATGGCTCTTGCTTGTGCAGCACCTCCACTTTTCGGATGGTCAAGGTACATTCCAGAAGGCATGCAATGCTCCTGTGGACCTGACTACTACACCCTGGCTCCAGGATTCAACAATGAATCTTATGTCATCTATATGTTTATTGTACATTTCTTCATTCCTGTTTTCCTGATTTTCTTCACCTATGGAAGCCTTGTCATGACAGTCAAAGCTGCCGCAGCTCAGCAGCAGGACTCGGCTTCTACACAGAAAGCTGAGAAAGAAGTAACGCGTATGTGTGTCTTGATGGTGTTGGGCTTCTTGGTAGCTTGGACGCCATATGCATCCTTCGCTGGCTGGATCTTTATGAATAAAGGAGCAGCTTTCACGGCTCTGACTGCAGCCCTGCCTGCTTTCTTTGCAAAGAGCTCAGCC---------------------------------------------------------------------------------------------------------------------------------------------------------------------------------------------------------------------------------------------------------------------------------------------------------------------------------------------------------------------------------------

>KX768660_Pminor_RH2_1

ATGGCTTGGGATGGCGGA------------------------TACGAGCCCAACGGCACAGAG---GGAAAAAACTTCTACATCCCGATGTCCAACAAAACCGGCATCGTCAGA------AGTCCCTTCGAA------TACCCGCAATACTACATGGTGGACCCAATGATCTACAAGCTCCTAGCCTTCTACATGTTCTTCCTGATCTGCACTGGGACTCCCATCAATGGCCTGACGCTGTTTGTAACAGCTCAGAACAAGAAGCTGCGCCAGCCTCTCAACTACATCCTGGTAAACTTGGCAGTGGCCGGACTCATCATGTGCGCCTTCGGTTTCACCATCACCATCACATCTGCTCTTAATGGCTACTTCATTCTTGGACCCACCTTCTGTGCTATTGAGGGATTCATGGCGACACTCGGAGGTGAAGTTGCTCTCTGGTCTCTGGTTGTTCTGGCTATTGAGAGATATATTGTTGTGTGCAAACCTATGGGCAGCTTCAAATTCACTGGA---------ACTCATGCTGCTGCTGGAGTTTCTACCACTTGGATCATGGCTCTTGCTTGTGCAGCACCTCCACTTTTCGGATGGTCAAGGTACCTTCCAGAAGGCATGCAGTGCTCCTGTGGACCTGACTACTACACCCTGGCTCCAGGATTCAACAATGAATCTTATGTCATCTACATGTTTGTTGTTCATTTCTTCATCCCTGTTTTCCTGATTTTCTTCACCTATGGAAGCCTCGTCATGACAGTCAAAGCTGCTGCAGCTCAGCAGCAGGACTCGGCTTCCACGCAGAAAGCCGAGAAGGAAGTAACACGTATGTGTGTCTTGATGGTGATGGGCTTCCTGGTAGCTTGGGTGCCATACGCAACCTTCGCTGGCTGGATCTTTTTGAACAAAGGAGCGGCTTTCACGGCTCTGACTGCAGCCCTGCCTGCTTTCTTTGCAAAGAGCTCAGCCCTTTACAACCCTGTTATCTATGTCCTGATGAACAAACAGTTCCGTAACTGCATGCTCACCACT---ATT---------------------------------------------------------------------GGAATGGGCGGC---ATGGTGGAGGACGAGACT------------TCAGTTTCAACG------AGCAAG---ACAGAA---------------------------------------------------GTCTCGACTGCTGCTTAA------------------------------------------------------------------------------------------

>HQ391990_Preticulata_RH2_1

---------------------------------------------------------------------------------------------------------------------------------------------------------ATGGTGGACCCAATGATCTACAAGCTCCTAGCCTTCTACATGTTCTTTCTGATCTGCACTGGAACTCCCATCAATGGCCTGACGCTGTTTGTAACGGCTCAGAACAAAAAGCTGCGCCAGCCTCTCAACTACATCCTGGTGAACTTGGCAGTGGCCGGACTCATCATGTGCGCCTTCGGTTTCACCATCACCATCACATCTGCTCTTAACGGTTACTTCATTCTTGGACCCACCTTCTGTGCTATTGAGGGATTCATGGCAACACTCGGAGGTGAAGTTGCTCTCTGGTCTCTGGTTGTTCTGGCTATTGAGAGATATATTGTTGTGTGCAAACCTATGGGCAGCTTCAAATTCACTGGA---------ACTCATGCTGCTGCCGGAGTTACTTCCACATGGATCATGGCTCTTGCTTGTGCAGCACCTCCACTTTTCGGATGGTCAAGGTACCTTCCAGAAGGCATGCAGTGCTCCTGTGGACCTGACTACTACACCCTGGCTCCAGGATTCAACAATGAATCTTATGTCATCTACATGTTTGTTGTTCATTTCTTCATTCCTGTTTTCCTGATTTTCTTCACCTATGGAAGCCTCGTCATGACAGTCAAAGCTGCCGCAGCTCAGCAGCAGGACTCGGCTTCCACACAGAAAGCCGAGAAGGAAGTAACACGAATGTGTGTCTTGATGGTGTTGGGCTTCCTGGTAGCTTGGACGCCATACGCAACCTTCGCTGGCTGGATCTTTATGAACAAAGGAGCGGCTTTCACGGCTCTGACTGCAGCCCTGCCTGCTTTCTTTGCGAAGAGCTCAGCTCTTTACAATCCTGTTATC---------------------------------------------------------------------------------------------------------------------------------------------------------------------------------------------------------------------------------------------------------------------------------------------------------------------------------------------------------------------

>KX768663_Pcaymanensis_RH2_1

ATGGCTTGGGATGGCGGA------------------------TACGAGCCCAACGGCACGGAG---GGAAAAAACTTCTACATCCCAATGTCCAACAAAACCGGCATCGTCAGA------AGTCCCTTCGAA------TACCCGCAGTACTACATGGTGGACCCAATGATCTACAAGCTCCTAGCCTTCTACATGTTCTTCCTGATCTGCACCGGGACTCCCATCAATGGCCTGACGCTGTTTGTAACAGCTCAGAACAAGAAGCTGCGCCAGCCTCTCAACTACATCCTGGTGAACTTGGCAGTGGCCGGACTCATCATGTGCGCCTTCGGTTTCACCATCACCATCACATCTGCTCTTAACGGTTACTTCATTCTTGGACCCACCTTCTGTGCTATTGAGGGATTCATGGCAACACTTGGAGGTGAAGTTGCTCTCTGGTCTCTGGTTGTTCTGGCTATTGAGAGATATATTGTTGTGTGCAAACCTATGGGCAGCTTCAAATTCACTGGT---------ACTCATGCTGCTGCTGGAGTTACTTCCACATGGATCATGGCTCTTGCTTGTGCAGCACCTCCACTTTTCGGATGGTCAAGGTACCTTCCAGAAGGCATGCAGTGCTCCTGTGGACCTGACTACTACACCCTGGCTCCAGGATTCAACAATGAATCTTATGTCATCTACATGTTTGTTGTTCATTTCTTCATTCCTGTTTTCCTGATTTTCTTCACCTATGGAAGCCTCGTCATGACAGTCAAAGCTGCCGCAGCTCAGCAGCAGGACTCAGCTTCCACACAGAAAGCCGAGAAGGAAGTAACACGTATGTGTGTCTTGATGGTGTTGGGCTTCCTGGTAGCTTGGACGCCATACGCAACCTTCGCTGGCTGGATCTTTTTGAACAAAGGAGCGGCTTTCACGGCTCTGACTGCAGCCCTGCCTGCTTTCTTTGCAAAGAGCTCAGCCCTTTACAATCCTGTTATCTATGTCCTGATGAACAAACAGTTCCGTAACTGCATGCTCACCACT---ATT---------------------------------------------------------------------GGAATGGGCGGC---ATGGTGGAGGACGAGACC------------TCAGTTTCAACG------AGCAAG---ACAGAA---------------------------------------------------GTCTCGACTGCTGCTTAA------------------------------------------------------------------------------------------

>KX768664_Hformosa_RH2_1

ATGGCTTGGGATGGCGGA------------------------TATGAGCCCAATGGCACGGAG---GGAAAAAACTTCTACATCCCAATGTCCAACAAAACCGGCATCGTCAGA------AGTCCCTTCGAA------TACCCGCAATACTACATGGTGGACCCAATGATCTACAAGCTCCTCGCCTTCTACATGTTCTTCCTGATCTGCACTGGAACTCCCATCAATGGCCTGACACTGTTTGTAACTGCTCAGAACAAAAAGCTGCGCCAGCCTCTCAACTACATCCTGGTGAACTTGGCTGTGGCCGGACTCATCATGTGCGCCTTCGGTTTCACCATCACCATCACATCTGCTCTTAACGGTTACTTCATTCTTGGACCCACCTTCTGTGCTATTGAGGGATTCATGGCAACACTTGGAGGTGAAGTTGCTCTCTGGTCTCTGGTTGTTCTGGCTATTGAGAGATACATTGTTGTGTGCAAACCTATGGGAAGCTTCAAATTCACTGGA---------ACTCATGCTGCTGCTGGAGTTACTTCCACATGGATCATGGCTCTTGCTTGTGCAGCACCTCCACTTTTCGGATGGTCAAGGTACCTTCCAGAAGGCATGCAGTGCTCCTGTGGACCTGACTACTACACCCTGGCTCCAGGATTCAACAATGAATCTTATGTCATCTACATGTTTGTTGTTCATTTCTTCATTCCTGTTTTCCTGATTTTCTTCACCTATGGAAGCCTCGTCATGACAGTCAAAGCTGCCGCAGCTCAGCAGCAGGACTCGGCTTCGACACAGAAAGCTGAGAAAGAAGTAACACGTATGTGTGTCTTGATGGTGTTGGGCTTCTTGGTAGCTTGGACGCCATATGCAACCTTCGCTGGCTGGATCTTTATGAACAAAGGAGCAGCTTTCACGGCTCTGACTGCAGCCCTGCCTGCTTTCTTTGCAAAGAGCTCAGCCCTTTACAATCCTGTTATCTATGTCCTGATGAACAAACAGTTCCGTAACTGCATGCTCTCCACT---ATT---------------------------------------------------------------------GGAATGGGCGGC---ATGGTGGAGGACGAAACC------------TCAGTTTCAACG------AGCAAG---ACAGAA---------------------------------------------------GTCTCGACTGCTGCTTAA------------------------------------------------------------------------------------------

>KX768662_Lnigrofasciata_RH2_1

ATGGCTTGGGATGGCGGA------------------------TACGAGCCCAATGGCACGGAG---GGAAAAAACTTCTACATCCCAATGTCCAACAAAACCGGCATCGTCAGA------AGTCCCTTCGAA------TACCCGCAGTACTACATGGTGGACCCAATGATCTACAAGCTCTTAGCCTTCTACATGTTCTTCCTGATCTGCACCGGGACTCCCATCAATGGCCTGACGCTGTTTGTAACAGCTCAGAACAAGAAGCTGCGCCAGCCTCTCAACTACATCCTGGTGAACTTGGCAGTGGCCGGACTCATCATGTGCGCCTTCGGTTTCACCATCACTATCACATCTGCTCTTAACGGTTACTTCATTCTTGGACCCACCTTCTGTGCTATTGAGGGATTCATGGCAACACTTGGAGGTGAAGTTGCTCTCTGGTCTCTGGTTGTTCTGGCTATTGAGAGATATATTGTTGTGTGCAAACCTATGGGCAGCTTCAAATTCACTGGT---------ACTCATGCTGCTGCTGGAGTTACTTCCACATGGATCATGGCTCTTGCTTGTGCAGCACCTCCACTTTTCGGATGGTCAAGGTACCTTCCAGAAGGCATGCAGTGCTCCTGTGGACCTGACTACTACACCCTGGCTCCAGGATTCAACAATGAATCTTATGTCATCTACATGTTTGTTGTTCATTTCTTCATTCCTGTTTTCCTGATTTTCTTCACCTATGGAAGCCTCGTCATGACAGTCAAAGCTGCCGCAGCTCAGCAGCAGGACTCAGCTTCCACACAGAAAGCCGAGAAGGAAGTAACACGTATGTGTGTCTTGATGGTGTTGGGCTTCCTGGTAGCTTGGACGCCATACGCAACCTTCGCTGGCTGGATTTTTTTGAACAAAGGAGCGGCTTTCACGGCTCTGACTGCAGCCCTGCCTGCTTTCTTTGCAAAGAGCTCAGCCCTTTACAATCCTGTTATCTATGTCCTGATGAACAAACAGTTCCGTAACTGCATGCTCAGCACT---ATT---------------------------------------------------------------------GGAATGGGCGGC---ATGGTGGAGGACGAGACC------------TCAGTTTCAACG------AGCAAG---ACAGAA---------------------------------------------------GTCTCGACTGCTGCTTAA------------------------------------------------------------------------------------------

>KX768654_Pvelifera_RH2_1

ATGGCTTGGGATGGTGGA------------------------TACGAGCCCAATGGCACGGAG---GGAAAAAACTTATACATCCCGATGTCCAACAAAACCGGCATTGTCAGA------AGTCCCTTCGAA------TACCCGCAATACTACATGGTGGACCCAATGATCTACAAGCTCCTAGCCTTCTACATGTTCTTCCTGATCTGCACTGGAACTCCCATCAATGGCCTGACGCTGTTTGTAACAGCTCAGAACAAAAAGCTGCGCCAGCCTCTCAACTACATCCTGGTGAACTTGGCAGTGGCCGGACTCATCATGTGCGCCTTCGGTTTCACCATCACCATCACATCTGCTCTTAATGGTTACTTCATTCTTGGACCCACCTTCTGTGCTATTGAGGGATTCATGGCAACACTCGGAGGTGAAGTTGCTCTCTGGTCTCTGGTTGTTCTGGCTATTGAGAGATATATTGTTGTGTGCAAACCTATGGGCAGCTTCAAATTCACTGGA---------ACTCATGCTGCTGCTGGAGTTACTTCCACATGGATCATGGCTCTTGCTTGTGCAGCACCTCCACTTTTCGGATGGTCAAGGTACCTTCCAGAAGGCATGCAGTGCTCCTGTGGACCTGATTACTACACCCTGGCTCCAGGATTCAACAATGAATCTTATGTCATCTACATGTTTGTCGTTCATTTCTTCATTCCTGTTTTCCTGATTTTCTTCACCTATGGAAGCCTCGTCATGACAGTCAAAGCTGCCGCAGCTCAGCAGCAGGACTCGGCTTCCACACAGAAAGCCGAGAAGGAAGTAACACGTATGTGTGTCTTGATGGTGCTGGGCTTCCTGGTAGCTTGGACTCCATACGCAACCTTCGCTGGCTGGATCTTTTTGAACAAAGGAGCGGCATTCACGGCTCTGACTGCAGCCCTGCCTGCTTTCTTTGCAAAGAGCTCAGCCCTTTACAATCCTGTTATCTATGTCCTGATGAACAAACAGTTCCGTAACTGCATGCTCAGCACT---ATT---------------------------------------------------------------------GGAATGGGCGGC---ATGGTGGAGGATGAGACC------------TCAGTTTCAACG------AGCAAG---ACAGAA---------------------------------------------------GTCTCGACTGCTGCTTAA------------------------------------------------------------------------------------------

>KX768659_Mbifurca_RH2_1

ATGGCTTGGGATGGCGGA------------------------TACGAGCCCAATGGCACGGAG---GGAAAAAACTTCTACATCCCGATGTCCAACAAAACCGGCATCGTCAGA------AGTCCCTTCGAA------TACCCGCAATACTACATGGTGGACCCAATGATCTACAAGCTCCTAGCCTTCTACATGTTCTTCCTGATCTGCACTGGAACTCCCATTAATGGCCTGACGCTGCTTGTAACAGCTCAGAACAAAAAGCTGCGCCAGCCTCTCAACTACATCCTGGTGAACTTGGCAGTGGCCGGACTCATCATGTGCGCCTTCGGTTTCACCATCACCATCACATCTGCTCTTAATGGTTACTTCATTCTTGGACCCACCTTCTGCGCTATTGAGGGATTCATGGCAACACTCGGAGGTGAAGTTGCTCTCTGGTCTCTGGTTGTTCTGGCTATTGAGAGATATATTGTTGTGTGCAAACCTATGGGCAGCTTCAAATTCACTGGA---------ACTCATGCTGCTGCTGGAGTTACTTCCACATGGATCATGGCTCTTGCTTGTGCAGCACCTCCACTTTTCGGATGGTCAAGGTACCTTCCAGAAGGCATGCAGTGCTCCTGTGGACCTGATTACTACACCTTGGCTCCAGGATTCAACAATGAATCTTATGTCATCTACATGTTTGTCGTTCATTTCTTCATTCCTGTTTTCCTGATTTTCTTTACCTGTGGAAGCCTCGTCATGACAGTCAAAGCTGCCGCAGCTCAGCAGCAGGACTCGGCTTCCACACAGAAAGCCGAGAAGGAAGTAACACGTATGTGTGTCTTGATGGTGCTGGGCTTCCTGGTAGCTTGGACTCCATACGCAACCTTCGCTGGCTGGATCTTTTTGAACAAAGGAGCGGCATTCACGGCTCTGACTGCAGCCCTGCCTGCTTTCTTTGCAAAGAGCTCAGCCCTTTACAATCCTGTTATCTATGTCCTGATGAACAAACAGTTCCGTAACTGCATGCTCAGCACT---ATT---------------------------------------------------------------------GGAATGGGCGGC---ATGGTGGAGGATGAGACC------------TCAGTTTCAACG------AGCAAG---ACAGAA---------------------------------------------------GTCTCGACTGCTGCTTAA------------------------------------------------------------------------------------------

>AB223055_Olatipes_RH2_C

ATGGGCTGGGATGGAGGA---------------------------GAGCAAAATGGAACCGAG---GGAAAGAACTTCTACATCCCAATGTCCAACCGGACGGGGGTTGTTAGA------AGCCCCTATGAA------TACCCTCAGTACTACATGGTCGACCCAATTATGTACAAGATTCTGGCTTTCTACATGTTCTTCCTGATCTGCACTGGAACTCCCATCAACGGCTTGACATTGTACGTAACAGCCACCAACAAGAAGCTTCAGCAACCTCTCAACTTCATCCTGGTGAACCTGGCGGTGGCCGGACTCATCATGTGCGCCTTTGGCTTCACCATTACCCTCAATTCAAGCTTCTATGGCTATTTTGTTCTTGGACCCACCTTCCGTGCCGTTGAGGGATTTATGGCCACACTTGGAGGTCAAATTGCTCTCTGGTCCCTGGTGGTCCTGGCTGTTGAGAGATATATTGTTGTCTGCAAACCCATGGGGAGCTTCAAATTCACAGGA---------ACTCACGCCGCAGCTGGAGTCCTTTTCACCTGGGTAATGGCTCTGGCTTGTGCTGCTCCTCCACTGCTTGGTTGGTCCAGGTACATCCCTGAAGGCATGCAATGCTCCTGTGGACCTGACTACTACACTCTGGCTCCTGGCTTCAACAATGAGTCATACGTCATGTATATGTTCTGCGTGCACTTCTGCATCCCAGTCTTCCTCATTTTCTTCACTTATGGAAGTCTGGTGTTGACTGTCAAAGCTGCAGCAGCGCAGCAGCAGGACTCAGCTTCTACCCAGAAGGCTGAGAAGGAGGTAACACGTATGTGCCTGTTGATGGTCTTTGGCTTCCTGGTAGCTTGGGTTCCGTACGCCAGCTTTGCTGCTTGGATTTTCCTGAACAAAGGAGCTTCCTTCACTGCCCTGACTGCCTCCATCCCTGCTTTCTTTGCTAAGAGCTCAGCATTGTACAATGCTGTTATCTACGTGCTGCTGAACAAACAGTTCCGTAACTGCATGCTGGCTGCC---ATT---------------------------------------------------------------------GGAATGGGAGGC---TTGGTGGAGGATGAGACC------------TCAGTGTCAACA------AGCAAG---ACAGAA---------------------------------------------------GTCTCAACTGCAGCTTAA------------------------------------------------------------------------------------------

>HQ260683_Preticulata_RH2_2

---------------------------------------------------------------------------------------------------------------------------------------------------------------GATCCATGGCAATTCAAATTATTAGCTGCGTACATGTTCTTCCTGATCATCACCGGCTTCCCAATCAACGGTCTGACACTGGTGGTCACAGCTCAGAACAAGAAGCTCCGGCAACCTCTCAACTATATCCTGGTGAACTTGGCGGTAGCAGGATTGATCATGGTCATCTTTGGATTCACGACCACCATTTACTCTTCTATGGTGGGCTATTTTGCTATGGGACCCTTAGGCTGTGATATGGAAGGTTTCTTTGCTACGATTGGAGGTCAAGTATCACTATGGTCTCTTGTGGTCTTAGCTATTGAGAGATACATAGTGGTCTGTAAACCAATGGGTAGTTTTAAATTTACTGCT---------ACCCATTCTGCAATTGGCTGTGGATTTACCTGGGTCATGGCTCTCTCCTGTGCGGGTCCTCCTTTGGTGGGCTGGTCAAGATATATTCCTGAGGGTCTTCAGGTATCTTGTGGACCTGACTATTACACCTTGGCCCCAGGTTTCAACAACGAATCCTACGTGATGTACTTGTTTTCCTGCCACTTCTGTTTCCCTGTCTTCACAATCTTCTTCACATATGGGAGTCTTGTGATGACAGTCAAAGCTGCCGCAGCCCAGCAGCAGGACTCAGCTTCCACTCAGAAAGCTGAGAGGGAAGTGACGCGTATGTGCGTCCTGATGGTTGTCGGCTTCCTTCTTGCCTGGGTCCCCTACGCTTCCTTTGCTGCGTGGATTTTCTTT---------------------------------------------------------------------------------------------------------------------------------------------------------------------------------------------------------------------------------------------------------------------------------------------------------------------------------------------------------------------------------------------------------------------------------------------------------

>GU454733_Xhellerii_RH2_2

------------------------------------------------------------------------------------------------------------------------------------------------------------------------------------------------------------------------------------------------------------------------------------------------------------------------------------------------------------------------------------------------------------ATGGAAGGTTTCTTTGCTACAATTGGAGGTCAAGTATCACTATGGTCTCTTGTGGTCTTAGCTATTGAGAGATACATAGTAGTCTGTAAACCAATGGGTAGTTTTAAATTTACTGCC---------ACCCATTCTGCAATTGGCTGTGGATTTACCTGGGTCATGGCTCTCTCCTGTGCGACTCCTCCTTTGGTGGGCTGGTCAAGGTATATTCCTGAGGGTCTTCAGGTATCCTGTGGACCTGACTATTACACGTTGGCCCCAGGTTTCAACAATGAATCATACGTGATGTACTTGTTCTCCTGCCACTTCTGTTTCCCTGTCTTCACAATCTTCTTCACATATGGGAGTCTTGTGATGACAGTCAAAGCTGCTGCAGCCCAGCAGCAGGACTCAGCTTCCACTCAGAAAGCTGAGAAGGAAGTGACACGTATGTGCGTCCTGATGGTTGTCGGCTTCCTTCTTGCTTGGGTCCCCTATGCTTCCTTCGCTGCGTGGATTTTCTTTAACAGA---------------------------------------------------------------------------------------------------------------------------------------------------------------------------------------------------------------------------------------------------------------------------------------------------------------------------------------------------------------------------------------------------------------------------------------------------

>MN817660_Gaffinis_SWS2A

---------------------------------------------AACCGAGTCGTAGACTTT---CCAGAAGACTTCTGGATCCCAGTCCCGTTGGACACAGACAACATCTCATCCCTCAGCCCTTTCTCG------GTTCCTCAGGACCACTTGGGGAATTCGGGAATGTTCTACGCCATGGCCGGATTCACGTTCTTTCTTTTTGTCGTCGGCACTTCCATCAACATCCTCACCATCGCGTGCACCCTGCGGTACAAGAAGCTTCGCTCCCATCTCAACTACATCCTGGTGAACTTGGCCGTGGCGAACCTTCTGGTGTCTGTGGTCGGCTCCTTCACCGCCTGCTGCTCCTTCACATTCAGATATTTCATCTTTGGGCCGCTAGCGTGCAAGATCGAAGGGTTTGTCGCAACGCTAGGGGGTATGGTAAGTCTTTGGTCTTTGGCAGTGGTAGCTTTTGAAAGATGGCTGGTCATCTGCAAACCTCTTGGCAATGTTGCTTTCAAGCCT---------GAACATGCGATGGCTTGCTGCGTGTTCACCTGGATCTTCGCTTTGACAGCCTCAGTTCCGCCCTTGCTGGGATGGAGCAGGCACATCCCAGAAGGCCTCCAGTGCTCCTGCGGTCCAGACTGGTACACAACCAACAACAAATACAACAACGAGTCCTACGTCATGTTCCTTTTCTGCTTCTGCTTTGCCGTACCATTCACCACCATTATTTTTTGTTATTCTCAGCTACTCGTAACACTTAAAATGGTAGCAAAGGCCCAAGCTGAGTCGGCCTCCACCCAGAAGGCTGAAAAGGAGGTGACCAGGATGGTGGTCGTCATGGTGCTGGGCTTTTTGGTATGCTGGATGCCCTACACCTCCTTCGCTCTTTGGGTCGTCAACAACCGTGGGCAAACGTTCGACCTGAGATTAGCAACCCTACCATCCTGCCTGTCGAAGGCCTCCACCGTCTACAACCCCGTCATCTATGTATTCCTCAATAAACAGTTTCGAACATGCATGTTATCCATG---CTG---------------------------------------------------------------------GGGATGGGG------------GATGGAGAGGAG------------GAAGTCTCTACAACTCAGTCAGTG---ACTGAA---------------------------------------------------GTCTCCAAAGTTGGCCCGGCTTAG------------------------------------------------------------------------------------

>KX768636_Hformosa_SWS2A

ATG------------------------------------AGGTTCAACTCAGTCGTAGAGTTT---CCAGAAGACTTCTGGATCCCAGTCCCGTTGGACACAGACAACATCTCATTCCTCAGCCCTTTCTCG------GTTCCTCAGGACCACTTGGGGAATTCGGGAATGTTCTACGCCATGGCCGGATTCACATTCTTTCTTTTTGTCGTCGGCTCTTCCATCAACACCCTCACCATCGCGTGCACCGTGCGGTACAAGAAGCTTCGCTCCCATCTCAACTACATCCTGGTGAACTTGGCCGTGGCAAACCTTCTGGTGTCTGTGGTCGGCTCCTTTACGGCCTGCTGCTCCTTCACGTTCAGATATTTCATCTTTGGGCCGTTAGCGTGCAAGATCGAAGGGTTTGTTGCAACGCTAGGGGGTATGGTAAGCCTTTGGTCCTTGGCAGTGGTAGCTTTTGAAAGATGGCTGGTCATCTGCAAACCTCTTGGCAATGTTGCTTTCAAACCT---------GAACATGCAATGTCTTGCTGCATGTTCACCTGGATCTTCGCATTGATAGCCTCAGTTCCACCCTTGCTGGGATGGAGCAGGTACATCCCAGAAGGCCTCCAGTGCTCGTGCGGTCCAGACTGGTACACAACCAACAACAAATACAACAACGAGTCCTACGTCATGTTTCTTTTCTGCTTCTGCTTTGCCGTTCCGTTCACCACCATCATTTTTTGTTATTCTCAGCTACTCATAACACTTAAAATGGTAGCAAAGGCCCAAGCTGAGTCGGCCTCCACACAGAAGGCTGAAAAGGAGGTGACCAGGATGGTGGTCGTCATGGTGCTGGGCTTTTTGGTATGCTGGATGCCCTACGCCTCCTTTGCTCTTTGGGTTGTCAACAACCGTGGGCAAACGTTCGACCTGAGATTAGCAACCCTACCATCCTGCCTGTCGAAGGCCTCCGCCGTCTACAATCCCGTCATCTATGTATTCCTCAATAAGCAGTTTCGAACATGCATGTTATCCATG---CTG---------------------------------------------------------------------GGGATGGGT------------GATGGAGAGGAG------------GAATTCTCTACAACTCAGTCAGTG---ACTGAA---------------------------------------------------GTCTCCAAAGTTGGCCCGGCTTAG------------------------------------------------------------------------------------

>KX768632_Pminor_SWS2A

ATG------------------------------------AGGTCCAACCGAGTATTAGAGTTT---CCAGAAGACTTCTGGATCCCAATCCCGTTGGACACAGACAACATCTCATCCCTCAGCCCTTTCTTG------GTCCCTCAGGACCACTTGGGGAATTCGGGAACATTCTACGCCATGGCCGGATTCACGTTCTTTCTTTTTGTCGTTGGCACTTCCATCAACACCCTCACCATCATGTGCACCATGCGGTACAAGAAGCTTCGCTCCCACCTCAACTACATCCTGGTGAACTTGGCCGTGGCGAACCTTCTGGTGTCTGTGGTCGGCTCCTTCACCATTTGCTTCTCCTTCACATTCAGATATTTCATCTTTGGGCCGCTAGCGTGCAAGATCGAAGGGTTTGTCGCAACGCTAGGGGGTATGGTAAGCCTTTGGTCTTTGGCAGTGGTAGCTTTTGAAAGATGGCTGGTCATCTGCAAACCTCTAGGCAATGTTGCTTTCAAGCCT---------GAACATGCCACGGCTTGCTGCGTGATCACCTGGATCTTTGCGTTGACAGCCTCAGTTCCACCCTTGCTGGGATGGAGCAGGTACATCCCAGAAGGCCTACAGTGCTCCTGTGGTCCAGACTGGTACACAACCAACAACAAATACAACAACGAGTCCTACGTCATGTTTCTCTTCTGCTTCTGCTTCGCCGCTCCATTCACCACCATCATTTTTTGTTATTCTCAGCTACTTGTAACACTTAAAGTGGTAGCAAAGGCCCAAGCTGAGTCTGCCTCCACCCAGAAGGCTGAGAAGGAGGTGACCAGGATGGTGGTCGTCATGGTGCTGGGCTTCTTGGTGTGTTGGATGCCCTATGCCTCCTTTGCTCTTTGGGTCGTCAACAACCGTGGGCAAAAGTTCGACCTCAGATTAGCAACCCTGCCATCCTGCCTGTCAAAGGCCTCCACCGTCTACAATCCCGTCATCTACGTATTCCTCAATAAGCAGTTTCGAACATGCATGCTAAACATG---CTG---------------------------------------------------------------------GGGATGGGC------------GATGGAGAGGAG------------GAAGTCTCTACAACTCAGTCAGTG---ACTGAA---------------------------------------------------GTCTCCAAAGTTGGTCCGGCTTAG------------------------------------------------------------------------------------

>KX768624_Pvelifera_SWS2A

ATG------------------------------------AGGTCCAACCGAGTTGTAGAGTTT---CCAGAAGACTTCTGGATCCCAATCCCGTTGGACACAGACAACATCTCATCCCTCAGCCCTTTCTTG------GTCCCTCAAGATCACTTGGGGAATTCGGGAACGTTCTACGCCATGGCTGGATTCACGTTCTTTCTTTTTGTCGTTGGCACTTCCATCAACACCCTCACCATCGTATGCACAATGCGGTACAAGAAGCTTCGCTCCCACCTCAACTACATCCTGGTGAACTTGGCCGTGGCAAACCTTCTGGTGTCTGTGGTCGGCTCCTTCACCACCTGCTTCTCTTTCACATTCAGATATTTCATCTTTGGGCCACTAGCGTGCAAGATCGAAGGGTTTGTCGCAACACTAGGGGGTATGGTAAGCCTTTGGTCTTTGGCAGTGGTAGCGTTTGAAAGATGGCTGGTCATCTGCAAACCTCTTGGCAATGTTGCTTTCAAGCCT---------GAACATGCAACGGCTTGCTGCGTGATCACCTGGATCTTCGCATTGACAGCCTCAGTTCCACCCTTGCTGGGATGGAGCAGGTATATCCCAGAAGGCCTCCAGTGCTCCTGTGGTCCAGACTGGTACACAACCAACAACAAATACAACAACGAGTCCTACGTCATGTTTCTCTTCTGTTTCTGCTTCGCCGCTCCATTCACCACCATCATTTTTTGTTATTCTCAGCTACTCGTAACACTTAAAATGGTAGCAAAGGCCCAAGCTGAGTCGGCCTCCACCCAGAAGGCTGAGAAGGAGGTGACCAGGATGGTGGTCGTCATGGTGCTGGGCTTCTTGGTGTGCTGGATGCCCTACGCCTCCTTCGCTCTTTGGGTCGTCAACAACCGTGGGCAAAAGTTCGACCTGAGATTAGCAACCCTGCCATCCTGCCTGTCGAAGGCCTCCACCGTCTACAATCCCGTCATCTACGTATTCCTCAATAAGCAGTTTCGAACATGCATGCTAAACATG---CTG---------------------------------------------------------------------GGGATGGGG------------GATGGAGAGGAG------------GAAGTCTCTACAACTCAGTCAGTG---ACTGAA---------------------------------------------------GTCTCCAAAGTTGGTCCAGCTTAG------------------------------------------------------------------------------------

>KX768635_Pcaymanensis_SWS2A

ATG------------------------------------AGGTCCAACCAAGTCGTAGAGTTT---CCAGAAGACTTCTGGATCCCAATCCCATTGGACACAGACAACATCTCATCCCTCAGCCCTTTCTTG------GTCCCTCAAGACCACTTGGGGAATTCGGGAACATTCTACGCCATGGCCGGATTCACGTTCTTTCTTTTTGTCGTTGGCACTTCCATCAACACCCTCACCATTGTGTGCACCATGCGGTACAAGAAGCTTCGCTCCCACCTCAACTACATCCTGGTGAACTTGGCCGTGGCAAACCTTCTAGTGTCTGTGGTCGGCTCCTTCACCACCTGCTTCTCCTTCACATTCAGATATTTCATCTTTGGGCCGCTAGCATGCAAAATCGAAGGGTTTGTCGCAACGCTAGGGGGTATGGTAAGCCTTTGGTCTTTGGCAGTGGTAGCTTTTGAAAGATGGCTGGTCATCTGCAAACCTCTTGGCAATGTTGCTTTCAAGCCT---------GAACATGCGTCGGCTTGCTGCGCGATCACCTGGATCTTCGCGTTGACAGCCTCAGTTCCACCCTTGTTGGGATGGAGCAGGTACATCCCAGAAGGCCTACAGTGCTCCTGTGGTCCAGACTGGTACACAACCAACAACAAATACAACAACGAATCCTACGTCATGTTTCTCTTCTGCTTCTGCTTTGCCGCTCCATTCACCACCATCATTTTTTGTTATTCTCAGCTACTCATAACACTTAAAATGGTAGCAAAGGCCCAAGCTGAGTCGGCCTCCACCAAAAAGGCTGAGAAGGAGGTGACCAGGATGGTGGTCGTCATGGTGCTGGGCTTCTTGGTGTGCTGGATGCCCTACGCCTCCTTCGCTCTTTGGGTCGTCAACAACCGTGGGCAAAAGTTCGACCTGAGATTAGCAACCCTGCCATCCTGCCTGTCGAAGGCCTCCACCGTCTACAATCCCGTCATCTACGTATTCCTCAATAAGCAGTTTCGAACATGCATGCTAAACATG---CTG---------------------------------------------------------------------GGGATGGGG------------GATGGAGAGGAG------------GAAGTCTCTACAACTCAGTCAGTG---ACTGAA---------------------------------------------------GTCTCCAAAGTTGGTCCAGCTTAG------------------------------------------------------------------------------------

>KX768634_Lnigrofasciata_SWS2A

ATG------------------------------------AGGTCCAACCGAGTCGTAGAGTTT---CCAGAAGACTTCTGGATCCCAATCCCATTGGACACAGACAACATCTCATCCTTCAGCCCTTTCTTG------GTCCCTCAGGACCACTTGGGGAATTCGGGAACATTCTACGCCATGGCCGGATTCACGTTCTTTCTTTTTGTCGTTGGCACTTCCATCAACACCCTCACCATTGTGTGCACCATGCGGTACAAGAAGCTTCGCTCCCACCTCAACTACATCCTGGTGAACTTGGCCGTGGCAAACCTTCTAGTGTCTGTGGTCGGCTCCTTCACCACCTGCTTCTCCTTCACATTCAGATATTTCATCTTTGGGCCGCTAGCATGCAAAATCGAAGGGTTTGTCGCAACGCTAGGGGGTATGGTAAGCCTTTGGTCTTTGGCAGTGGTAGCTTTTGAAAGATGGCTGGTCATCTGCAAACCTCTTGGCAATGTTGCTTTCAAGCCT---------GAACATGCGACGGCTTGCTGCGCGATCACCTGGATCTTCGCGTTGACAGCCTCAGTCCCACCCTTGTTGGGATGGAGCAGGTACATCCCAGAAGGCCTACAGTGCTCCTGTGGTCCAGACTGGTACACAACCAACAACAAATACAACAACGAGTCCTATGTCATGTTTCTCTTCTGCTTCTGCTTCGCCGCTCCATTCACCACCATCATTTTTTGTTATTCTCAGCTACTCATAACACTTAAAATGGTAGCAAAGGCCCAAGCTGAGTCGGCCTCCACCAAAAAGGCTGAGAAGGAGGTGACCAGGATGGTGGTCGTCATGGTGCTGGGCTTCTTGGTGTGCTGGATGCCCTACGCCTCCTTCGCTCTTTGGGTCGTCAACAACCGTGGGCAAAAGTTCGACCTGAGATTAGCAACCTTGCCATCCTGCCTGTCGAAGGCCTCCACCGTCTACAATCCCGTCATCTACGTATTCCTCAATAAGCAGTTTCGAACATGCATGCTAAACATG---CTG---------------------------------------------------------------------GGGATGGGG------------GATGGAGAGGAG------------GAAGTCTCTACAACTCAGTCAGTG---ACTGAA---------------------------------------------------GTCTCCAAAGTTGGTCCAGCTTAG------------------------------------------------------------------------------------

>KX768631_Mbifurca_SWS2A

ATG------------------------------------AGGTCCAACCGAGTCATAGAGTTT---CCAGAAGACTTCTGGATCCCAATCCCGTTGGACACAGACAACATTTCATCCCTCAGCCCTTTCTTG------GTCCCTCAGGACCACTTGGGGAATTCGGGAACGTTTTACGCCATGGCCGGATTCACGTTCTTTCTTTTTGTCGTCGGCACTTCCATCAACACCCTCACCATCGTGTGCACCATGCGGTACAAGAAGCTTCGCTCCCACCTCAACTACATCCTGGTAAACTTGGCCGTGGCGAACCTTCTGGTGTCTGTGGTCGGCTCCTTCACCACCTGCTTCTCCTTCACATTCAGATATTTCATCTTTGGGCCGCTAGCATGCAAGATCGAAGGGTTTGTCGCAACGCTAGGCGGTATGGTAAGCCTTTGGTCTTTGGCAGTTGTAGCTTTTGAAAGATGGCTGGTCATCTGCAAACCTCTTGGCAATGTTGCTTTCAAGCCT---------GAACATGCGACGGCTTGCTGTGCGATCACCTGGATCTTCGCGTTGACAGCCTCAGTTCCACCCTTGCTGGGATGGAGCAGGTATATCCCAGAAGGCCTCCAGTGCTCCTGCGGTCCAGACTGGTACACAACCAACAACAAATACAACAACGAGTCCTACGTCATGTTTCTCTTCTGCTTCTGCTTCGCCGCTCCATTCACCACCATCATTTTTTGTTATTCTCAGCTTCTCATAACACTTAAAATGGCAGCAAAGGCCCAAGCTGAGTCGGCCTCCACCCAGAAGGCTGAGAAGGAGGTGACTAGGATGGTGGTCGTCATGGTGCTGGGCTTCTTGGTGTGCTGGATGCCCTACGCCTCCTTCGCTCTTTGGGTCGTCAACAACCGTGGGCAAAGGTTCGACCTGAGATTAGGAACCCTGCCATCCTGCCTGTCGAAGGCCTCCACCGTCTACAATCCCGTCATCTACGTAGTCCTCAATAAGCAGTTTCGAACATGCATGATAAACATG---CTG---------------------------------------------------------------------GGGATGGGG------------GACGGAGAGGAG------------GAAGTCTCTACAACTCAGTCAGTG---ACTGAA---------------------------------------------------GTCTCCAAAGTTGGTCCAGCTTAG------------------------------------------------------------------------------------

>AB223056_Olatipes_SWS2_A

ATG------------------------------------AGGTTCATCAGTGGTGGGGAGCTG---CCAGATGACTTCTGGATACCAATTCCTTTGGACACTAATAACTTATCATTACTTAGCCCTTTCCTT------GTTCCTCAGGACCACTTGGGAAGTTCAGGGACTTTCTACGCAATGGCAGCCTTCATGTTCTTTCTGTTCGTGTTTGGCACTTCAATTAACAGCCTCACCATTGCATGCACCTTTCAAAACAAGAAGCTTCGATCCCACCTGAACTACATCCTGGTGAACCTGTCTGTGGCTAATCTTCTTGTGTCTGGCGTGGGCTCCTCCACTGCCTTTTGCTCCTTCGCTTGCAGATATTTTGTTTTTGGATCGCTGGCATGTAAGATTGAAGGTTTCGCAGCAACACTTGGAGGAATGGTGGGCCTGTGGTCTCTTGCTGTGATAGCCTTTGAAAGATGGCTGGTCATCTGCAAGCCACTTGGTAACTTTACTTTCAAGCCA---------GAACACGCGCTGGCTTGCTGCTTGGTTACCTGGGTGTGTGCTCTGGCAGCTGCAGCCCCTCCTCTGGCCGGATGGAGCAGGTACATCCCTGAAGGCCTGCAGTGCTCCTGTGGACCAGACTGGTACACAACAAACAACAAATACAACAATGAGTCCTATGTGATGTTTCTCTTCTGTTTCTGCTTCGCGGTTCCCTTCGCCACCATCGTCTTTTGCTACTCTCAGCTGCTAGTCACCCTGAAAATGGCAGCCAAGGCCCAAGCAGAGTCTGCGTCCACCCAGAAAGCAGAGCGAGAGGTGACCAGGATGGTGGTTGTCATGGTGCTGGGCTTTCTGGTATGCTGGATGCCGTATGCCTCCTTTGCTCTTTGGGTTGTCAACAATCGCGGCCACTCCTTTGACCTGAGGCTGGCGACCATTCCATCCTGCCTTTCAAAGGCCTCCACTGTGTACAATCCAGTTATTTATGTCCTCCTAAACAAGCAGTTCCGTTCCTGCATGTTGACGATG---CTG---------------------------------------------------------------------GGAATGGGT------------GGAGGAGAAGAG------------GAGGACTCAACA------TCAGTG---ACTGAA---------------------------------------------------GTCTCTAAAGTCGGACCAGCTTAG------------------------------------------------------------------------------------

>GU454734_Xhellerii_SWS1

------------------------------------------------------------------------------------------------------------------------------------------------------------------------------------------------ATGGGCTTCGTGTTCTTTGCAGGAACGCCGCTGAACCTGCTGGTCCTCCTGGCCACGGCCAAGTACAAGAAGCTCCGAGCTCCTCTCAACTACATCCTGGTCAACATCTCCTTCGCTGGCTTCATCTTCGTCACCTTCTCCGTCAGCCAGGTGTTCGTGGCCAGTCTGAGGGGCTACTACTTCCTGGGCTACACCTTGTGTGCATTGGAAGCGGCCATGGGCGCCGTGGCAGGTCTGGTGACGTCCTGGTCGCTCGCCGTGCTCTCCTTCGAGAGGTATCTGGTGATCTGTAAGCCGTTCGGAGCCTTCAAGTTCGGCAGC---------ACCCACGCCGCCGCCGCCGTGGTCTTCACCTGGTGCATGGGGGTGGGCTGCGCCTCGCCGCCTTTCTTCGGCTGGAGCAGGTACATCCCGGAAGGCCTGGGCTGCTCCTGCGGACCTGACTGGTACACCCACAACGAGGAGTACGGCTGCACCAGCTACATGTACTTCCTGCTGATCACCTGCTTCTGCATGCCTCTGTCCATCATCATCTTCTCCTACTCGCAGCTGCTGGGCGCGCTGAGAGCCGTTGCGGCGCAGCAGGCGGAGTCCGCCTCCACCCAGAAGGCTGAGAAGGAGGTGTCCAGGATGATCATCGTGATGGTGGGCTCCTTCGTCACCTGCTACGGCCCCTACGCCCTGACGGGCCTCTGGTACGCTAACTCAGAGGAAGTCAACAAACAC------------------------------------------------------------------------------------------------------------------------------------------------------------------------------------------------------------------------------------------------------------------------------------------------------------------------------------------------------------------------------------------------------------------------------------

>MN817658_Gaffinis_SWS1

---------------------------------------------------------------------------------------------------------------------------CCCTTCGAG------GGGTCCCAGCACTACCTGGCCCCGGCGTGGGCCTTTCACCTGCAGGCGCTCTTCATGGGCTTCGTCTTCTTTGTGGGAACGCCGCTGAACCTGCTGGTCCTCCTGGCCACGGCCAAATACAAGAAGCTCCGAGCTCCTCTCAACTACATCCTGGTCAACATCTCCTTCGCTGGCTTCATCTTTGTCACCTTCTCCGTCAGCCAGGTGTTTGTGGCCAGTCTGAGGGGCTACTACTTCCTGGGCTACACCTTGTGTGCATTGGAAGCGGCCATGGGCGCTGTGGCAGGTCTGGTGACATCCTGGTCACTCGCCGTGCTCTCCTTCGAGAGGTACTTGGTGATCTGTAAGCCGTTCGGAGCCTTCAAGTTCGGCAGC---------AGCCACTCTGCCGCCGCCGTGGTCTTCACCTGGTGCATGGGGGTGGGCTGCGCCTCACCGCCTTTCTTCGGCTGGAGCAGGTACATCCCGGAAGGCCTGGGCTGCTCCTGTGGACCCGACTGGTACACCCACAATGAGGAGTATGGCTGCACCAGCTACATGTACTTCCTGCTGATCACCTGCTTCTGCATGCCTCTGTCCATCATCATCTTCTCCTACTCGCAGCTGCTGGGCGCGCTGAGAGCCGTTGCGGCGCAGCAGGCAGAGTCCGCCTCCACCCAGAAGGCTGAGAAGGAGGTGTCCAGGATGATTATCGTGATGGTGGGCTCCTTCGTCACCTGCTACGGCCCCTACGCCCTGACGGGCCTCTGGTACGCCAACTCAGAGGAAGTCAACAAAGACTATCGGCTCGTCACCATCCCGGCGTTCTTCTCCAAGAGCTCCTGCGTCTACAACCCGCTCATCTACGCCTTCATGAATAAACAGTTCAATGCTTGCATCATGGAAATGGTGTTT---------------------------------------------------------------------GGGAAGAAA---------ATGGAGGAAGCTTCC------------GAAGTGTCT---------TCAAAG---ACTGAA---------------------------------------------------GTGTCCACA---------------------------------------------------------------------------------------------------

>HQ260685_Preticulata_SWS1

------------------------------------------------------------------------------------------------------------------------------------------------------------------------------------CAGGCGCTCTTCATGGGCTTCGTCTTCTTTGCGGGAACGCCGCTGAACCTGCTGGTCCTCCTGGCCACGGCCAAGTACAAGAAGCTCCGAGCTCCTCTCAACTACATCCTGGTCAACATCTCCTTCGCTGGCTTCATCTTCGTCACCTTCTCCGTCAGCCAGGTGTTCGTGGCCAGTCTGAGGGGCTACTACTTCCTGGGTTACACCTTGTGTGCATTGGAAGCCGCCATGGGCGCCGTGGCAGGTCTGGTGACGTCCTGGTCGCTCGCCGTGCTCTCCTTCGAGAGGTTCCTGGTGATCTGTAAACCGTTCGGAGCCTTCAAGTTCGGCAGC---------ACCCACGCCGCCGCCGCCGTGGTCTTCACCTGGTTCATGGGGGTGGGCTGCGCCTCGCCGCCTTTCTTCGGCTGGAGCAGGTACATCCCGGAAGGCCTGGGCTGCTCCTGCGGACCCGACTGGTACACCCACAATGAAGAGTACGGCTGCACCAGCTACATGTATTTCCTGCTGATCACTTGCTTCTGCATGCCTCTGTCCATCATCATCTTCTCCTACTCGCAGCTGCTGGGCGCGCTGAGAGCCGTTGCGGCGCAGCAGGCGGAGTCCGCCTCCACCCAGAAGGCTGAGAAGGAGGTGTCCAGGATGATCATCGTGATGGTGGGCTCCTTCGTCACCTGCTACGGCCCCTACGCCCTGACGGGCCTCTGGTACGCCAACTCAGAGGAAGTCAACAAAGACTACCGGCTCGTCACCATCCCTGCGTTCTTCTCCAAGAGCTCCTGCGTCTACAACCCGCTC------------------------------------------------------------------------------------------------------------------------------------------------------------------------------------------------------------------------------------------------------------------------------------------------------------------------------------------------------------------------

>KX768639_Pvelifera_SWS1

ATG---------------------------------------------------------------GGGAAACATTTTCACCTGTACGAGAACATCTCCAAGGTG---------------GACCCCTTCGAG------GGGTCCCAGCACTACCTGGCCCCGGCGTGGGCCTTTCACCTGCAGGCGCTCTTCATGGGCTTCGTCTTCTTTGCGGGAACGCCGCTGAACCTGCTGGTCCTCCTGGCCACGGCCAAGTACAAGAAGCTCCGAGCTCCTCTCAACTACATCCTGGTCAACATCTCCTTCGCTGGCTTCATCTTCGTCACCTTCTCCGTCAGCCAGGTGTTCGTGGCCAGTCTGAGGGGCTACTACTTCCTGGGTTACACCTTGTGTGCATTGGAAGCGGCCATGGGCGCCGTGGCAGGTCTGGTGACGTCCTGGTCGCTTGCCGTGCTCTCCTTCGAGAGGTACCTGGTGATCTGTAAACCATTCGGAGCCTTCAAGTTCGGCAGC---------TCCCACGCCGCCGCCGCCGTGGTCTTCACCTGGTTCATGGGGGTGGGCTGCGCCTCGCCGCCTTTCTTCGGCTGGAGCAGGTACATCCCGGAAGGCCTGGGCTGCTCCTGCGGACCCGACTGGTACACCCACAATGAGGAGTACGGCTGCACCAGCTACATGTATTTCCTGCTGATCACCTGCTTCTGCATGCCTCTGTCCATCATCATCTTCTCCTACTCGCAGCTGCTGGGCGCGCTGAGAGCCGTTGCGGCGCAGCAGGCGGAGTCCGCCTCCACCCAGAAGGCTGAGAAGGAGGTGTCCAGGATGATCATCGTGATGGTGGGCTCCTTTGTCACCTGCTATGGCCCCTACGCCCTGACGGGCCTCTGGTACGCCAACTCAGAGGAAGCCAACAAAGACTACCGGCTCGTCACCATCCCTGCGTTCTTCTCCAAGAGCTCCTGCGTCTACAACCCGCTCATCTACGCCTTCATGAACAAACAGTTCAATGCCTGCATCATGGAAATGGTGTTT---------------------------------------------------------------------GGGAAGAAA---------ATGGAGGAAGCGTCT------------GAAGTTTCT---------TCAAAG---ACTGAG---------------------------------------------------GTGTCCACAGCTTCATAA------------------------------------------------------------------------------------------

>KX768650_Pcaymanensis_SWS1

ATG---------------------------------------------------------------GGGAAACATTTTCACCTGTACGAGAACATCTCCAAGGTG---------------GATCCCTTCGAG------GGGTCCCAGCACTACCTGGCCCCGGCGTGGGCCTTTCACCTGCAGGCACTCTTCATGGGCTTCGTCTTCTTTGCGGGAACGCCGCTGAACCTGCTGGTCCTCCTGGCCACGGCCAAGTACAAGAAGCTCCGAGCTCCTCTCAACTACATCCTGGTCAACATCTCCTTCGCTGGCTTCATCTTCGTCACCTTCTCTGTCAGCCAGGTGTTCGTCGCCAGTCTGAGGGGCTATTACTTCCTGGGTTATACCTTGTGTGCATTGGAAGCGGCCATGGGCGCTGTGGCAGGTCTGGTGACGTCCTGGTCGCTTGCCGTGCTCTCCTTCGAGAGGTACCTGGTGATCTGTAAACCGTTTGGAGCCTTCAAGTTCGGCAGC---------ACCCACGCCGCCGCCGCCGTGGTCTTCACCTGGTTCATGGGGGTGGGCTGCGCCTCGCCGCCTTTCTTTGGCTGGAGCAGGTACATCCCGGAAGGCCTGGGCTGCTCCTGCGGACCCGACTGGTACACCCACAATGAAGAGTACGGCTGCACCAGCTACATGTATTTCCTGCTGATCACCTGCTTCTGCATGCCTCTGTCCATCATCATCTTCTCCTACTCGCAGCTGCTGGGCGCACTGAGAGCCGTTGCGGCGCAGCAGGCGGAGTCCGCCTCCACCCAGAAGGCTGAGAAAGAGGTGTCCAGGATGATCATCGTGATGGTGGGCTCCTTTGTCACCTGCTACGGCCCCTACGCCCTGACGGGCCTCTGGTACGCCAACTCAGAGGAAGCTAACAAAGACTACCGGCTCGTCACCATCCCTGCGTTCTTCTCCAAGAGCTCCTGCGTCTACAACCCGCTCATCTACGCCTTCATGAACAAACAGTTCAATGCCTGCATCATGGAAATGGTGTTT---------------------------------------------------------------------GGGAAGAAA---------ATGGAGGAAGCATCT------------GAAGTGTCT---------TCAAAG---ACTGAG---------------------------------------------------GTGTCCACAGCTTCATAA------------------------------------------------------------------------------------------

>KX768649_Lnigrofasciata_SWS1

ATG---------------------------------------------------------------GGGAAACATTTTCACCTGTACGAGAACATCTCCAAGGTG---------------GATCCCTTCGAG------GGGTCCCAGCACTACCTGGCCCCGGCGTGGGCCTTTCACCTGCAGGCGCTCTTCATGGGCTTCGTCTTCTTTGTGGGAACGCCGCTGAACCTGCTGGTCCTCCTGGCCACGGCCAAGTACAAGAAGCTCCAAGCTCCTCTCAACTACATCCTGGTCAACATCTCCTTCGCTGGCTTCATCTTCGTCACCTTCTCCGTCAGCCAGGTGTTCGTCGCCAGTCTGAGGGGCTACTACTTCCTGGGTTATACCTTGTGTGCATTGGAAGCGGCCATGGGCGCCGTGGCAGGTCTGGTGACGTCCTGGTCGCTTGCCGTGCTCTCCTTCGAGAGGTACCTGGTGATCTGTAAACCGTTCGGAGCCTTCAAGTTCGGCAGC---------ACCCACGCCGCCGCCGCCGTGGTCTTCACCTGGTTCATGGGGGTGGGCTGCGCCTCGCCGCCTTTCTTTGGCTGGAGCAGGTACATCCCGGAAGGCCTGGGCTGCTCCTGCGGACCCGACTGGTACACCCACAATGAAGAGTACGGCTGCACCAGCTACATGTATTTCCTGCTGATCACCTGCTTCTGCATGCCTCTGTCCATCATCATCTTCTCCTACTCGCAGCTGCTGGGCGCACTGAGAGCCGTTGCGGCGCAGCAGGCGGAGTCCGCCTCCACCCAGAAGGCTGAGAAAGAGGTGTCCAGGATGATCATCGTGATGGTGGGCTCCTTTGTCACCTGCTACGGCCCCTACGCCCTGACGGGCCTCTGGTACGCCAACTCAGAGGAAGCCAACAAAGACTATCGTCTCGTCACCATCCCTGCGTTCTTCTCCAAGAGCTCCTGCGTCTACAACCCGCTCATCTACGCCTTCATGAACAAACAGTTCAATGCCTGCATCATGGAAATGGTGTTT---------------------------------------------------------------------GGGAAGAAA---------ATGGAGGAAGCGTCT------------GAAGTGTCT---------TCAAAG---ACTGAG---------------------------------------------------GTGTCCACAGCTTCATAA------------------------------------------------------------------------------------------

>KX768651_Hformosa_SWS1

ATG---------------------------------------------------------------GGGAAACATTTTCACCTGTACGAGAACATCTCCAAGGTG---------------GACCCCTTCGAG------GGGTCCCAGCACTACTTGGCCCCGGCGTGGGCCTTTCACCTGCAGGCGCTCTTCATGGGCTTCGTCTTCTTTGCGGGAACGCCGCTGAACCTGCTGGTCCTCCTGGCCACGGCCAAGTACAAGAAGCTCCGAGCTCCTCTCAACTACATCCTGGTCAACATCTCCTTCGCTGGCTTCATCTTCGTCACCTTCTCCGTCAGCCAGGTGTTCGTGGCCAGTCTGAGGGGCTACTACTTCCTGGGCTACACCTTGTGTGCATTGGAAGCGGCCATGGGCGCCGTGGCAGGTCTGGTGACGTCCTGGTCGCTTGCCGTGCTCTCCTTCGAGAGGTACCTGGTGATCTGTAAACCATTCGGAGCCTTCAAGTTCGGCAGC---------ACCCACGCCGCCGCCGCCGTGGTCTTCACCTGGTTCATGGGAGTGGGCTGCGCCTCGTCGCCTTTCTTCGGCTGGAGCAGGTACATCCCGGAAGGCCTGGGCTGCTCCTGCGGACCCGACTGGTACACCCACAATGAGGAGTACGGCTGCACCAGCTACATGTATTTCCTGCTGATCACCTGCTTCTGCATGCCTCTGTCCATCATCATCTTCTCCTACTCGCAGCTGCTGGGCGCGCTGAGAGCCGTTGCGGCGCAGCAGGCGGAGTCTGCCTCCACCCAGAAGGCTGAGAAGGAGGTGTCCAGGATGATCATCGTGATGGTGGGCTCCTTCGTCACCTGCTACGGCCCCTACGCCCTGACGGGCCTCTGGTACGCCAACTCAGAGGAAGCCAACAAAGACTACCGGCTCGTCACCATCCCTGCATTCTTCTCCAAGAGCTCCTGCGTCTACAACCCGCTCATCTACGCCTTCATGAACAAACAGTTCAATGCCTGCATCATGGAAATGGTGTTT---------------------------------------------------------------------GGGAAGAAA---------ATGGAGGAAGCGTCT------------GAAGTTTCT---------TCAAAG---ACTGAG---------------------------------------------------GTGTCCACAGCTTCATAA------------------------------------------------------------------------------------------

>KX768646_Mbifurca_SWS1

ATG---------------------------------------------------------------GGGAAACATTTCCACCTGTACGAGAACATCTCCAAGGTG---------------GACCCGTTCGAG------GGGTCCCAGCACTACCTGGCCCCCGCCTGGGCCTTTCACCTGCAGGCGCTTTTCATGGGCTTCGTCTTCTTCGCCGGGACGCCGCTGAACCTGCTGGTCCTGCTGGCCACCGCCAAGTACAAGAAGCTCCGAGCTCCGCTCAACTACATCCTGGTCAACATCTCGTTCGCCGGCTTCATCTTCGTCACCTTCTCCGTCAGCCAGGTGTTCGTGGCCAGTCTGAGGGGCTACTACTTCCTGGGTTACACCCTGTGTGCCTTGGAAGCGGCCATGGGCGCCGTGGCAGGCCTGGTGACGTCCTGGTCGCTCGCCGTCCTGTCCTTCGAAAGGTTCCTGGTGATCTGCAAACCGTTCGGAGCCTTCAAGTTCGGCAGC---------AGCCACGCCGGCGCCGCCGTGGTCTTCTCCTGGTGCATGGGGGTCGGATGCGCCTCGCCGCCTTTCTTCGGCTGGAGCAGGTGCATCCCGGAAGGCCTGGGCTGCTCCTGCGGACCCGACTGGTACACACACAACGAGGAGTACGGCACCACCAGCTACATGTACTTCCTGCTGATCACCTGCTTCTGCATGCCTCTGTCCATCATCATCTTCTCCTATTCGCAGCTGCTGGGCGCGCTGAGAGCCGTGGCGGCGCAGCAGGCGGAGTCCGCCTCCACCCAGAAGGCGGAGAAGGAGGTGTCCAGGATGATCATCGTGATGGTGGGATCCTTCGTCACCTGCTACGGCCCCTACGCCCTGACGGGCCTCTGGTACGCCAACTCAGAGGAAGCCAACAAAGACTACAGACTCGTCACCATCCCTGCGTTCTTCTCCAAGAGCTCCTGCGTCTACAACCCGCTGATCTACGCCTTCATGAACAAACAGTTCAATGCCTGCATCATGGAAATGGTGTTT---------------------------------------------------------------------GGAAAGAAA---------ATGGAGGAAGCGTCT------------GAAGTGTCT---------TCAAAG---ACTGAG---------------------------------------------------GTGTCCACAGCTTCATAA------------------------------------------------------------------------------------------

>KX768647_Pminor_SWS1

ATG---------------------------------------------------------------GGGAAACATTTTCACCTGTATGAGAACATCTCCAAGGTG---------------GATCCCTTCGAG------GGGTCCCAGCACTACCTGGCCCCGGCGTGGGCCTTTCACCTCCAGGCGCTCTTCATGGGCTTCGTCTTCTTTGCGGGAACGCCGCTGAACCTGCTGGTTCTCCTGGCCACGGCCAAGTACAAGAAGCTCCGAGCTCCTCTCAACTACATCCTGGTCAACATCTCCTTTGCTGGCTTCATCTTCGTCACCTTCTCCGTCAGCCAGGTGTTCATGGCCAGTCTGAGGGGCTACTACTTCCTGGGTTACACCTTGTGTGCATTGGAAGCGGCCATGGGCGCCGTGGCAGGTCTGGTGACGTCCTGGTCGCTTGCAGTGCTCTCCTTCGAGAGGTACCTGGTAATCTGTAAACCGTTCGGAGCCTTCAAGTTCGGCAGC---------ACCCACGCCGCCGCCGCCGTGGTCTTCACTTGGTGCATGGGGGTGGGCTGCGCCTCGCCGCCTTTCTTCGGCTGGAGCAGGTATATCCCGGAAGGCCTGGGCTGCTCCTGCGGACCCGACTGGTACACCCACAATGAAGAGTACGGCACCACCAGCTACATGTATTTCCTGCTGACCACCTGCTTCTGCATGCCTCTGTCCATCATCATCTTCTCCTACTCGCAGCTGCTGGGCGCGCTGAGAGCCGTTGCTGCACAGCAGGCAGAGTCCGCCTCCACCCAGAAGGCTGAGAAGGAGGTGTCCAGGATGATCATCGTGATGGTGGGCTCCTTCGTCTCCTGCTACGGCCCCTACGCCCTGACGGGCCTCTGGTACGCCAACTCAGAGGAAGCCAACAAAGACTACCGGCTCGTCACCATCCCTGCGTTCTTCTCCAAGAGCTCCTGTGTCTATAACCCGCTCATCTACGCCTTCATGAACAAACAGTTCAATGCCTGCATCATGGAAATGGTGTTT---------------------------------------------------------------------GGGAAGAAA---------ATGGAGGAAGCGTCT------------GAAGTGTCT---------TCAAAG---ACTGAG---------------------------------------------------GTGTCCACAGCTTCATAA------------------------------------------------------------------------------------------

>AB223058_Olatipes_SWS1

ATG---------------------------------------------------------------GGAAAATACTTCTACCTGTATGAGAACATCTCCAAAGTG---------------GGCCCCTACGAC------GGGCCCCAGTACTACCTGGCCCCGACGTGGGCCTTCTACCTGCAGGCGGCCTTCATGGGCTTCGTGTTCTTTGTGGGGACGCCTCTGAACTTTGTCGTTCTTCTGGCGACGGCCAAATACAAGAAGCTTCGAGTCCCGCTCAACTACATTCTTGTCAACATCACCTTTGCTGGTTTCATCTTCGTCACCTTCTCTGTCAGCCAGGTGTTCCTGGCCAGCGTGAGGGGCTACTACTTCTTTGGTCAAACGCTGTGCGCCCTGGAAGCAGCAGTGGGCGCTGTGGCAGGCCTCGTGACGTCCTGGTCTCTGGCCGTCCTCTCCTTTGAGAGATACCTGGTTATCTGTAAACCATTTGGAGCCTTCAAGTTTGGCAGC---------AACCACGCCCTGGCTGCTGTCATCTTCACCTGGTTCATGGGGGTGGGCTGCGCCTGCCCACCTTTCTTTGGCTGGAGTCGGTACATCCCTGAAGGTCTGGGCTGCTCCTGTGGACCGGATTGGTACACGAACTGCGAGGAGTTCAGTTGCGCCAGCTATTCCAAGTTCCTCCTGGTGACCTGCTTCATCTGCCCCATCACCATCATCATTTTCTCCTACTCCCAGCTCCTGGGCGCGCTCAGAGCGGTCGCAGCACAGCAGGCCGAGTCAGCGTCCACGCAGAAGGCCGAGAAGGAAGTGTCCAGGATGATCATCGTCATGGTGGCGTCCTTCGTCACCTGCTACGGTCCGTACGCTCTGACCGCCCAGTACTACGCATACTCCCAGGATGAGAACAAAGACTACCGGCTCGTCACCATCCCAGCTTTCTTCTCCAAGAGCTCCTGCGTGTACAACCCGCTCATCTACGCCTTCATGAACAAACAGTTTAACGGCTGCATCATGGAGATGGTTTTC---------------------------------------------------------------------GGAAAGAAG---------ATGGAGGAAGCGTCT------------GAGGTGTCC---------TCCAAG---ACGGAG---------------------------------------------------GTGTCCACGGCCTCTTAA------------------------------------------------------------------------------------------

>HQ260682_Preticulata_LWS_4

---------------------------------------------------------------------------------------------AATCATACAAGA---------------GATCCTTTTGAG------GGACCAAACTACCATATCGCTCCTCGATGGGTTTACAACATCACAACAGTCTGGATGTGTTTTGTGGTCGTCTTAGCAGTCTTCACAAATGGTCTGGTCTTGGTAGCCACAGCAAGGTTCAAGAAACTCCGTCATCCCCTGAACTGGATCTTAGTCAACCTTGCCATTGCCGACCTCGGAGAGACGGTCTTTGCCAGCACCATCAGCGTGTGCAACCAGTTTTTTGGATATTTTATTTTGGGACATCCAATGTGCGTCTTTGAAGGCTACGTTGTCTCGACTTGTGGTATTGCTGCTCTTTGGTCCCTGACTGTCATCTCTTGGGAGAGATGGATTGTTGTGTGCAAACCTTTTGGAAATACAAAGTTTGATGCC---------AAATGGGCGGCAGCTGGGATCATGTTCTCCTGGGTCTGGTCGGCAGTGTGGTGTGCTCCTCCCGTCTTTGGATGGAGCAGGTACTGGCCCCATGGGTTGAAAACGTCCTGTGGACCCGATGTGTTCAGTGGAAGTGAGGACCCCGGTGTCAAGTCCTACATGATTGTCCTCATGATTACATGCTGCATCACTCCTCTGGCTGTCATCATCTTGTGCTACCTGGCAGTGTGGTTGGCCATCCGTGACATTGCTATGCAGCAGAAGGAATGCGAGTCGACCCAGAACGCCCAGAAGGAAGTATCTAGGATGGTTGTTGTCATGATCTTGGCTTATTGTGTATGTTGGGGACCTTACACCTTTTTTGCTTGCTTTGCCGCAGCCAACTCCGGATATGCCTTCCACCCGTTAGCCGCTGCCATGCCTGCATACTTTGCCAAAAGTGCCACCATCTACAACCCGGTCATCTATGTCTTCATGAACCGACAGTTCCGCACATGCATCATGCGGCTT---TTT---------------------------------------------------------------------GGGAAAGAG---------GTGGATGACGGCTCT------------GAAGTGTCCACA------TCAAAG---ACAGAG---------------------------------------------------GTCTCCTCTGTGGCTCCTGAATAA------------------------------------------------------------------------------------

>KX768573_Pminor_LWS_R

ATGGCAGAAGATTGGGGAAAGCAGGCGTTTGCTCCCTGGAAGAACAACGAAGAAACTACAAGG---GGCTCTGCTTTCACATACACAAACAGCAATCATACAAGA---------------GATCCTTTTGAG------GGACCAAACTACCACATCGCTCCCCGATGGGTTTACAACATCACGACAGTCTGGATGTGTTTTGTGGTCGTCTTATCAGTCTTCACAAACGGTCTGGTCTTGGTAGCCACGGCAAGGTTCAAGAAACTCCGTCATCCCCTGAACTGGATCTTAGTCAACCTTGCCATCGCCGACCTCGGAGAGACGGTCTTCGCCAGCACCATCAGCGTGTGCAACCAGTTTTTTGGGTATTTTATTTTGGGACATCCAATGTGCGTCTTTGAAGGCTATGTTGTCTCGACTTGTGGTATTACTGCACTTTGGTCCCTGACTGTCATCTCTTGGGAGAGATGGATTGTCGTGTGCAAACCTTTTGGAAATACCAAGTTTGATGCC---------AAATGGGCCACAGCTGGGATCGTGTTCTCCTGGGTCTGGTCGGCAGTGTGGTGTGCTCCTCCTGTCTTTGGATGGAGCAGGTATTGGCCCCATGGGTTGAAAACGTCCTGTGGACCCGATGTGTTCAGTGGAAGCGAGGACCCCGGTGTCAAGTCCTACATGATTGTCCTCATGATTACGTGCTGCGTAGCTCCTCTGGCTGTCATTGTCTTGTGCTACCTGGCAGTGTGGCTGGCCATCCGTGACATTGCTATGCAGCAGAAGGAATGCGAGTCGACCCAGAACGCCCAGAAGGAAGTATCTAGGATGGTTGTTGTCATGATCTTGGCTTATTGTGTTTGTTGGGGACCTTACACCTTGTTTGCTTGCTTTGCCGCAGCCAACCCCGGATATGCCTTCCACCCGTTAGCCGCTGCCTTACCTGCATACTTTGCCAAAAGTGCCACCATCTACAACCCGGTCATCTATGTCTTCATGAACCGACAGTTCCGCACATGCATCATGCGGCTT---TTT---------------------------------------------------------------------GGGAAAGAG---------GTGGATGACGGCTCT------------GAAGTGTCCACC------TCAAAG---ACAGAG---------------------------------------------------GTCTCCTCTGTGGCTCCTGAATAA------------------------------------------------------------------------------------

>KX768567_Pvelifera_LWS_R

ATGGCAGAAGATTGGGGAAAGCAGGCGTTTGCTCCCTGGAAGAACAATGAAGAAACTACAAGG---GGCTCTGCTTTCACATACACAAACAGCAATCATACAAGA---------------GATCCTTTTGAG------GGACCAAACTACCACATTGCTCCCCGATGGGTTTACAACATCACAACAGTCTGGATGTGTTTTGTGGTCGTCTTATCAGTCTTCACAAATGGTCTGGTCTTGGTAGCCACGGCAAGGTTCAAGAAACTCCGTCATCCCCTGAACTGGATCTTAGTCAACCTTGCCATTGCTGATCTCGGAGAGACGGTCTTTGCCAGCACCATCAGCGTGTGCAACCAGTTTTTTGGATATTTTATTTTGGGACATCCAATGTGCGTCTTTGAAGGCTACGTTGTCTCGACTTGTGGTATTGCTGCTCTTTGGTCCCTGACTGTCATCTCTTGGGAGAGATGGATTGTTGTATGCAAACCTTTTGGAAATACCAAGTTTGATGCC---------AAATGGGCGACAGCTGGGATCGTGTTCTCCTGGGTCTGGTCGGCAGTGTGGTGTGCTCCTCCTGTCTTTGGATGGAGCAGGTACTGGCCCCATGGGTTGAAAACGTCCTGTGGACCCGATGTGTTCAGTGGAAGTGAGGACCCCGATGTCAAGTCCTACATGATTGTCCTCATGATTACATGCTGCGTCACTCCTCTGGCTGTCATCGTCTTGTGCTACCTGGCAGTGTGGTTGGCCATCCGTGACATTGCTATGCAGCAGAAGGAATGCGAGTCGACCCAGAACGCCCAGAAGGAAGTATCTAGGATGGTTGTTGTCATGATCTTGGCTTTTTGTGTATGTTGGGGACCTTACGCCTTTTTTGCTTGCTTTGCCGCAGCCAACCCTGGATATGCCTTCCACCCGTTAGCTGCTGCCATACCTGCATACTTTGCCAAAAGTGCCACCATCTACAACCCGGTCATCTATGTCTTCATGAACCGACAGTTCCGCACATGTATCATGCGGCTT---TTT---------------------------------------------------------------------GGGAAAGAG---------GTGGACGATGGCTCT------------GAAGTGTCCACC------TCAAAG---ACAGAG---------------------------------------------------GTCTCCTCTGTGGCTCCTGAATAA------------------------------------------------------------------------------------

>KX768575_Lnigrofasciata_LWS_R

ATGGCAGAAGATTGGGGAAAGCAGGCGTTTGCTCCCTGGAAGAACAATGAAGAAACTACAAGG---GGCTCTGCTTTCACATACACAAACAGCAATCATACAAGA---------------GATCCTTTTGAG------GGACCAAACTACCACATCGCTCCCCGATGGGTTTACAACATCACAACAGCCTGGATGTGTTTTGTGGTTGTCTTATCAGTCTTCACAAACGGTCTGGTCTTGGTAGCCACAGCAAGGTTCAAGAAACTCCGTCATCCCCTGAACTGGATCTTAGTCAACCTTGCCATTGCCGACCTCGGAGAGACGGTCTTTGCCAGCACCATCAGCGTGTGCAACCAGTTTTTTGGATATTTTATTTTGGGACATCCAATGTGCGTCTTTGAAGGCTACGTTGTCTCGACTTGTGGTATTGCTGCTCTTTGGTCCCTGACTGTCATCTCTTGGGAGAGATGGATTGTTGTGTGCAAACCTTTTGGAAATACCAAGTTTGATGCC---------AAATGGGCGACAGCTGGGATCGCGTTCTCCTGGGTCTGGTCGGCAGTGTGGTGTGCTCCTCCCGTCTTTGGATGGAGCAGGTACTGGCCCCATGGGTTGAAAACGTCCTGTGGACCCGATGTGTTCAGTGGAAGTGAGGAGCCCGGTGTCAAGTCCTACATGATTGTCCTCATGATTACATGCTGCATCACTCCTCTGGCTGTCATCGTCTTGTGCTACCTGGCAGTGTGGTTGGCCATCCGTGACATTGCTATGCAGCAGAAGGAATGCGAGTCGACCCAGAATGCCCAGAAGGAAGTATCTAGGATGGTTGTTGTCATGATCTTGGCTTTTTGTGTATGTTGGGGACCTTACGCCTTTGTTGCTTGCTTTGCCGCAGCCAACCCTGGATATGCCTTCCACCCGTTAGCCGCTGCCATACCTGCATACTTTGCCAAAAGTGCCACCATCTACAACCCGGTCATCTATGTCTTCATGAACCGACAGTTCCGCACATGCATCATGCGGCTT---TTT---------------------------------------------------------------------GGGAAAGAG---------GTGGATGACGGCTCT------------GAAGTGTCCACC------TCAAAG---ACAGAG---------------------------------------------------ATCTCCTCTGTGGCTCCTGAATAA------------------------------------------------------------------------------------

>KX768576_Pcaymanensis_LWS_R

ATGGCAGAAGATTGGGGAAAGCAGGCGTTTGCTCCCTGGAAGAACAATGAAGAAACTACAAGG---GGCTCTGCTTTCACATACGCAAACAGCAATCATACAAGA---------------GATCCTTTTGAG------GGACCAAACTACCACATCGCTCCCCGATGGGTTTACAACATCACAACAGCCTGGATGTGTTTTGTGGTCGTCTTATCAGTCTTCACAAACGGTCTGGTCTTGGTAGCCACAGCAAGGTTCAAGAAACTCCGTCATCCCCTGAACTGGATCTTAGTCAACCTTGCCATTGCCGACCTCGGAGAGACGGTCTTTGCCAGCACCATCAGCGTGTGCAACCAGTTTTTTGGATATTTTATTTTGGGACATCCAATGTGCGTCTTTGAAGGCTACGTTGTCTCGACTTGTGGTATTGCTGCTCTTTGGTCCCTGACTGTCATCTCTTGGGAGAGATGGATTGTTGTGTGCAAACCTTTTGGAAATACCAAGTTTGATGCC---------AAATGGGCGACAGCTGGGATCGCGTTCTCCTGGGTCTGGTCGGCAGTGTGGTGTGCTCCTCCCGTCTTTGGATGGAGCAGGTACTGGCCCCATGGGTTGAAAACGTCCTGTGGACCCGATGTGTTCAGTGGAAGTGAGGAGCCCGGTGTCAAGTCCTACATGATTGTCCTCATGATTACATGCTGCATCACTCCTCTGGCTGTCATCGTCTTGTGCTACCTGGCAGTGTGGTTGGCCATCCGTGACATTGCTATGCAGCAGAAGGAATGCGAGTCGACCCAGAACGCCCAGAAGGAAGTATCTAGGATGGTTGTTGTCATGATCTTGGCTTTTTGTGTATGTTGGGGACCTTACGCCTTTGTTGCTTGCTTTGCCGCAGCCAACCCTGGATATGCCTTCCACCCGTTAGCCGCTGCCATACCTGCATACTTTGCCAAAAGTGCCACCATCTACAACCCGGTCATCTATGTCTTCATGAACAGACAGTTCCGCACATGCATCATGCGGCTT---TTT---------------------------------------------------------------------GGGAAAGAG---------GTGGATGACGGCTCT------------GAAGTGTCCACC------TCAAAG---ACAGAG---------------------------------------------------ATCTCCTCTGTGGCTCCTGAATAA------------------------------------------------------------------------------------

>KX768577_Hformosa_LWS_R

ATGGCAGAAGATTGGGGGAAACAGGCGTTTGCTCCCTGGAAGAACAATGAAGAAACTACAAGG---GGCTCTGCTTTCACATACACAAACAGCAATCATACAAAA---------------GATCCTTTTGAG------GGACCAAACTACCACATCGCTCCTCGATGGGTTTACAATATTACAACAGTCTGGATGTGTTTTGTGGTCGTCTCATCAGTCTTCACAAATGGCCTGGTCTTGGTAGCCACAGCAAAGTTTAAGAAACTCCGTCATCCCCTGAACTGGATCTTAGTCAACCTTGCCATTGCAGATCTTGGAGAGACGGTTTTTGCCAGCACTATCAGTGTGTGCAACCAGTTTTTTGGATATTTTATTTTGGGACATCCAATGTGCGTCTTTGAAGGCTACGTTGTCTCGACTTGTGGTATTGCTGCTCTTTGGTCTCTGACTGTCATCTCTTGGGAGAGATGGATTGTTGTATGCAAACCTTTTGGGAATACCAAGTTTGATGCC---------AAATGGGCAACAGCTGGGATCATGTTTTCCTGGGTCTGGTCAGCAGTGTGGTGTGCTCCTCCTCTCTTTGGATGGAGCAGGTACTGGCCTCATGGATTGAAAACTTCCTGTGGACCCGATGTGTTCAGTGGAAGTGAGGACCCCGGTGTCAAGTCCTACATGATCGTCCTCATGGTTACATGCTGCATCATTCCTCTGGCTGTCATCATCTTGTGCTACCTGGCAGTGTGGTTGGCCATTCGTGACATTGCTATGCAGCAGAAGGAATGCGAGTCAACCCAGAACGCCCAGAAGGAAGTATCTAGGATGGTTGTTGTCATGATCTTGGCTTATTGTATATGTTGGGGACCTTACACCGTTTTTGCTTGCTTTGCTGCAGCAAACCCTGGATATGCCTTCCACCCTTTAGCCGCTGCTATGCCTGCATACTTTGCCAAGAGTGCCACCATCTACAATCCGGTCATCTATGTCTTCATGAACCGACAGTTCTGCACATGCATCATGCGGCTC---TTT---------------------------------------------------------------------GGGAGAGAG---------GTGGATGACGGCTCT------------GAAGTGTCCACA------TCAAAG---ACAGAG---------------------------------------------------GTCTCCTCTGTGGCTCCTGCATAA------------------------------------------------------------------------------------

>KX768572_Mbifurca_LWS_R

ATGGCAGAAGATTGGGGAAAGCAGGCGTTTGCTCCCTGGAAGAACAATGAAGAAACTACAAGG---GGCTCTGCTTTCACATACACAAACAGCAATCATACAAGA---------------GATCCTTTTGAG------GGACCAAACTACCACATCGCTCCTCGATGGGTTTACAACATCACAACAGTCTGGATGTGTTTTGTGGTCGTCTTATCAGTTTTCACAAATGGTCTGGTCTTGGTAGCCACAGCAAGGTTCAAGAAACTCCGTCATCCCCTCAACTGGATCTTAGTCAACCTTGCCATTGCCGACCTCGGAGAGACGGTCTTTGCCAGCACCATCAGTGTGTGCAACCAGTTTTTTGGATATTTTATTTTGGGACATCCAATGTGCGTCTTTGAAGGCTACGTCGTCTCGACTTGTGGCATTGCTGCTCTTTGGTCCCTGACCGTCATTTCTTGGGAGAGATGGATTGTTGTATGCAAGCCTTTTGGAAATACCAAGTTTGATGCC---------AAATGGGCCACAGCTGGGATCGTGTTCTCCTGGGTCTGGTCGGCAGTGTGGTGTGCTCCTCCCGTCTTTGGATGGAGCAGGTACTGGCCCCATGGGTTGAAAACATCCTGTGGACCCGATGTGTTCAGTGGAAGTGAGGACCCTGGTGTCAAGTCCTACATGATTGTCCTCATGATTACATGCTGCGTCATTCCTCTGGCTGTCATCGTCTTGTGCTACCTGGCAGTGTGGTTGGCCATCAGCGACATTGATATGCAGCAGAAGGAATGCGAGTCGACCCAGAACGCCCAGAAGGAAGTATCTAGGATGGTTGTTGTCATGATCTTGGCTTATTGTGTATGTTGGGGACCTTACACCTTTTTTGCTTGCTTTGCCGCAGCCAACCCCGGATACGCCTTCCACCCGTTAGCCGCTGCCATGCCTGCATACTTTGCCAAAAGTGCCACCATCTACAACCCAGTTATCTATGTTTTCATGAACCGACAGTTCCGCACTTGCATCATGCGGCTT---TTT---------------------------------------------------------------------GGGAAAGAG---------GTGGATGACAGCTCT------------GAAGTGTCCACA------TCAAAA---ACAGAG---------------------------------------------------GTCTCCTCTGTGGCTCCTGAATAA------------------------------------------------------------------------------------

>AB223051_Olatipes_LWS_A

ATGGCAGAGGAGTGGGGAAAACAGGTTTTTGCTGCGAGGCGACACAATGAAGACACAACAAGA---GGCTCTGCTTTTACTTACACAAACAGCAATCACACCCGA---------------GATCCTTTTGAG------GGTCCTAACTACCACATTGCTCCTCGATGGGTCTACAACGTTGCCACAGTTTGGATGTTTTTTGTGGTGGTTCTGTCCGTCTTCACCAACGGCCTGGTCTTGGTGGCCACAGCAAAGTTCAAGAAACTGCGCCACCCACTAAACTGGATTTTGGTCAATCTTGCTATAGCTGACCTGGGAGAGACGGTGTTTGCCAGCACCATCAGTGTGTGCAACCAGTTCTTTGGTTACTTCATTCTGGGACACCCCATGTGCGTGTTTGAGGGCTATGTGGTCTCCACTTGTGGTATTGCTGCTCTTTGGTCTCTGACCATAATCTCCTGGGAAAGATGGGTAGTCGTGTGCAAACCATTTGGAAATGTCAAGTTTGATGCC---------AAGTGGGCCATAGGTGGAATCGTCTTCTCCTGGGTCTGGTCAGCAGTTTGGTGTGCACCTCCCGTCTTTGGATGGAGCAGGTACTGGCCTCATGGACTAAAAACCTCCTGTGGACCTGATGTGTTCAGTGGAAGCGATGACCCTGGAGTGCAGTCCTACATGATTGTTCTGATGATCACATGCTGCATCATTCCCCTGGCCATCATCATCCTGTGTTATCTTGCTGTCTGGCTGGCTATCCGTGCTGTTGCCATGCAGCAGAAGGAATCAGAGTCAACCCAGAAGGCTGAAAAAGAAGTGTCCAGGATGGTGGTGGTCATGATCGTTGCTTACTGTGTGTGCTGGGGACCCTACACCTTTTTCGCCTGCTTTGCTGCAGCCAACCCCGGATATGCCTTCCATCCTCTGGCTGCTGCCATGCCTGCTTATTTTGCAAAGAGCGCCACAATCTACAACCCCATCATCTATGTCTTCATGAACAGACAGTTCCGCACATGCATCATGCAGCTC---TTT---------------------------------------------------------------------GGCAAGCAG---------GTGGATGATGGTTCT------------GAAGTTTCTACA------TCAAAG---ACAGAG---------------------------------------------------GTCTCCTCTGTGGCTCCTGCATAA------------------------------------------------------------------------------------

>AB223052_Olatipes_LWS_B

ATGGCAGAGCAGTGGGGAAAACAGGTTTTTGCTGCGAGGCGACAAAATGAAGACACAACAAGA---GGCTCTGCTTTTACTTACACAAACAGCAATCACACTCGA---------------GATCCCTTTGAG------GGTCCTAACTACCACATTGCTCCTCGATGGGTCTACAACCTTGCCACACTTTGGATGTTTTTTGTGGTGGTTCTGTCCGTCTTCACCAACGGCCTGGTCTTGGTGGCCACAGCAAAGTTCAAGAAACTGCGCCACCCACTAAACTGGATTTTGGTCAATCTTGCCATAGCTGACCTGGGAGAGACGGTGTTTGCCAGCACCATCAGTGTGTGCAACCAGTTCTTTGGTTACTTCATTCTGGGACACCCCATGTGCGTGTTTGAGGGCTATGTGGTCTCCACTTGTGGTATTGCTGCTCTTTGGTCTCTGACCATAATCTCCTGGGAAAGATGGGTAGTCGTGTGCAAACCTTTTGGAAATGTCAAGTTTGATGCC---------AAGTGGGCCATAGGTGGAATCGTCTTCTCCTGGGTCTGGTCTGCAGTTTGGTGTGCACCTCCCGTCTTTGGATGGAGCAGGTACTGGCCTCATGGACTAAAAACCTCCTGTGGACCTGATGTGTTCAGTGGAAGCGATGACCCTGGAGTGCAGTCCTACATGATTGTTCTGATGATCACATGCTGCATCATTCCCCTGGCCATCATCATCCTGTGTTACCTTGCTGTCTGGCTGGCTATCCGTGCTGTTGCCATGCAGCAGAAGGAATCAGAGTCAACCCAGAAGGCTGAAAGAGAAGTGTCCAGGATGGTGGTGGTCATGATCGTTGCTTACTGTGTGTGCTGGGGACCCTACACCTTTTTCGCCTGCTTTGCTGCAGCCAACCCCGGATATGCCTTCCATCCTCTGGCTGCTGCCATGCCTGCTTATTTTGCAAAGAGCGCCACAATCTACAACCCCGTCATCTATGTCTTCATGAACAGACAGTTCCGCACATGCATCATGCAGCTC---TTT---------------------------------------------------------------------GGCAAACAG---------GTGGATGATGGTTCT------------GAAGTTTCTACA------TCAAAG---ACAGAG---------------------------------------------------GTCTCCTCTGTGGCTCCTGCATAA------------------------------------------------------------------------------------

>DQ075246_Xhellerii_LWS

---------------------------------------------------------------------------------------------------------------------------------------------------------------------------------------------------------------------------------------CTCGTCTTGGTGGCCACAGCAAAGTTCAAGAAACTTCGTCATCCTCTCAACTGGATCTTGGTCAACCTTGCCATTGCTGATCTTGGAGAGACAGTCTTCGCCAGCACTATCAGTGTGTGCAACCAGTTCTTTGGATATTTCATTCTGGGACACCCGATGTGTGTCTTCGAAGGCTATGTTGTCTCAACTTGTGGTATTGCTGCTCTTTGGTCCCTGACTATCATCTCTTGGGAGAGATGGATAGTTGTGTGCAAACCTTTTGGAAATGTCAAGTTCGATGCC---------AAGTGGGCCACAGCTGGAATAGTTTTCTCCTGGGTCTGGTCTGCAGTGTGGTGTGCTCCTCCCATCTTTGGATGGAGCAGGTATTGGCCTCATGGACTGAAAACATCCTGCGGGCCTGATGTGTTCAGTGGAAGTGAAGACCCTGGAGTCCAGTCCTACATGGTTGTCCTCATGATTACATGCTGCATCATTCCTCTGGCTATCATCATCTTGTGCTACCTGGCTGTGTGGTTGGCCATCCATGCTGTTGCTATGCAGCAGAAGGAATCTGAGTCGACCCAGAAGGCTGAGAAGGAAGTG------------------------------------------------------------------------------------------------------------------------------------------------------------------------------------------------------------------------------------------------------------------------------------------------------------------------------------------------------------------------------------------------------------------------------------------------------------------------------------------------------------------------------------------

>HQ260680_Preticulata_LWS_2

---------------------------------------------------------------------------------------------------------------------------------------------------------------------TGGGTTTACGATGTCGCAACGGTCTGGATGTGTATCGTGGTCGTTTTATCAGTCTTCACCAATGGCCTCGTTTTGGTGGCAACAGCAAAGTTCAAGAAACTTCGTCATCCTCTCAACTGGATCTTGGTCAACCTTGCCATTGCTGATCTTGGAGAGACTGTCTTTGCCAACACCATCAGTGTGTGCAACCAGTTCTTTGGATATTTCATTCTGGGACACCCAATGTGTGTCTTTGAAGGCTATGTTGTCTCAATTTGTGGAATTGCTGGGCTTTGGTCCCTGACTATCATCTCTTGGGAGAGATGGATAGTTGTGTGCAAACCATTTGGAAATGTCAAGTTTGACGCC---------AAGTGGGCCACAGCTGGAATACTTTTCTCCTGGGTCTGGCCTGCAGTGTGGTGTGCTCCTCCCATCTTTGGATGGAGCAGGTATTGGCCTCATGGACTGAAAACATCCTGTGGACCTGATGTGTTCAGTGGAAGTGAAGACCCTGGAGTCCAGTCCTACATGATTGTCCTCATGATTTCATGCTGCATCATTCCTCTGTCTATCATCATCTTGTGCTACCTGGCTGTATGGTTGGCCATCCGTGCTGTTGCTATGCAGCAGCTAGATAGTGAATCAACCCAGAAGGCTGAAAGAGAAGTGTCCAGGATGGTTATAGTCATGATCCTGGCTTTCTGTCTCTGCTGGGGACCATATGCCACTTTCGCCTGCTTTGCCGCAGCCAACCCCGGATACGCCTTCCATCCTTTGGCCGCTGCCATGCCTGCATACTTTGCCAAAAGCGCCACCATCTACAACCCTGTTATCTATGTCTTCATGAACCGACAGTTTCGCACATGCATCATGAGACTC---TTT---------------------------------------------------------------------GGCAAACAG---------ATGGATGATGATTCC------------GAAGTGTCCACA------TCGAAG---ACAGAG---------------------------------------------------GTCTCGTCTGTTGCACCTGAATAA------------------------------------------------------------------------------------

>KX768594_Mbifurca_LWS_2

ATGTCAGATGATTGGAGAAAACAGCCGTTTGCTGCCAGGCGGCACAATGAAGATACAACAAGG---GGCTCTTTATTCACATACACAAACAGCAATCAAACAAGA---------------GATCCTTTTGAA------GGACCAAACTACCACATCGCTCCTCGATGGGTTTATGATGTCGCAACAGTCTGGATGTGTATTGTGGTCGTTTTATCAGTCTTTACCAACGGCCTCGTTTTGGTGGCCACAGCAAAGTTCAAGAAACTTCGTCATCCTCTCAACTGGATCTTGGTCAACCTTGCCATTGCCGATCTTGGAGAGACAGTCTTTGCCAGTACCATCAGTGTGTGCAACCAGGTCTTTGGATATTTCATTCTGGGACACCCAATGTGTGTCTTTGAAGGCTATGTTGTCTCAATTTGTGGAATTGCTGGGCTTTGGTCCCTGACCATCATCTCTTGGGAAAGATGGATAGTTGTGTGCAAACCCTTTGGAAATGTCAAGTTTGATTCC---------AAGTGGGCCACAGCTGGAATACTTTTCTCCTGGGTCTGGCCTGCAGTGTGGTGCGCTCCTCCCATCTTTGGATGGAGCAGGTATTGGCCTCATGGACTGAAAACGTCCTGCGGACCTGATGTGTTCAGTGGAAGTGAAGACCCTGGAGTCCAGTCCTACATGATGGTCCTCATAATTACATGCTGCTTCATTCCTCTGGCTATCATCATCTTGTGCTACCTGGCAGTGTGGTTGGCCATCCGTGCTGTTGCTATGCAGCAGCTAGATAGTGAATCAACTCAGAAGGCTGAGAGAGAAGTGTCCAGGATGGTTGTAGTCATGATCCTGGCTTTCTGTCTCTGCTGGGGACCATATGCCACTTTCGCCTGCTTTGCCGCAGCCAACCCCGGATATGCATTCCATCCTTTGGCTGCTGCCATCCCTGCGTATTTAGCCAAAAGCGCTACCATCTATAACCCTGTTATCTATGTCTTCATGAATCGACAGTTTCGCACCTGCATCATGAAACTC---TTT---------------------------------------------------------------------GGCAAACAG---------ATGGATGATGATTCC------------GAAGTGTCCACA------TCGAAG---ACAGAG---------------------------------------------------GTCTCATCTGTTGCACCTGAATAA------------------------------------------------------------------------------------

>KX768599_Hformosa_LWS_2

ATGGCAGATGACTGGAAGAAACAGATGTTTGCTGCCAGGCGGCACAATGAAGATACAACAAGG---GGCTCCGCATTCATATACACAAACAGCAATCAAACAAGA---------------GATCCTTTTGAA------GGACCAAACTACCACATCGCTCCTCGATGGGTTTACAACGTCGCCACACTCTGGATGTGTATCGTGGTTGTTTTATCAATCTTCACCAACGGCCTCGTCTTGGTGGCCACAGCAAAGTTCAAGAGACTTCGTCATCCTCTCAACTGGATCTTGGTCAATCTTGCCATTGCTGATCTTGGAGAGACAGTCTTTGCCAGCACCATCAGTGTGTGCAACCAGATCTTTGGATATTTCATTCTGGGACACCCAATGTGTGTCTTCGAAGGCTATGTTGTCTCAATTTGTGGAATTGCTGGACTTTGGTCCCTGACTATCATCTCTTGGGAGAGATGGATAGTTGTGTGCAAACCCTTTGGAAATGTCAAGTTCGATGCC---------AGGTGGGCCACAGCTGGAATAGCTTTCTCCTGGGTCTGGCCTGCAGTGTGGTGTGCTCCTCCCATCTTTGGATGGAGCAGGTACTGGCCTCATGGACTGAAAACATCCTGTGGGCCTGATGTGTTCAGTGGAAGTGATGACCCAGGAGTCCAGTCCTACATGATTGTCCTTATGGTTACCTGCTGCATCATTCCTCTTTCTATCATCATCCTGTGCTACCTGGCTGTGTGGTTGGCCATCCGTGCTGTTGCTATGCAACAGCTAGATAGTGAATCAACCCAGAAGGCTGAGAGAGAAGTGTCCAGGATGGTTGTGGTAATGATCCTGGCTTTCTGTCTCTGCTGGGGACCATATGCCTCTTTTGCCTGCTTTGCCGCAGCCAACCCCGGATACGCCTTCCATCCTTTGGCTGCTTCCATCCCTGCATATTTAGCCAAAAGCGCCACCATCTATAACCCTGTTATCTATGTCTTCATGAACCGACAGTTCCGCACATGCATTATGAAACTC---TTT---------------------------------------------------------------------GGGAAAGAG---------ATGGATGATGATTCC------------GAAGTGTCCACA------TCGAAG---ACAGAA---------------------------------------------------GTCTCATCTGTTGCACCTGAATAA------------------------------------------------------------------------------------

>KX768595_Pminor_LWS_2

ATGTCAGATGATTGGAGAAAACAGCTGTTTGCTGCCAGGCGGCACAATGAAGATACAACAAGG---GGCTCTGCATTCACATACACAAACAGCAATCAAACAAGA---------------GATCCCTTTGAA------GGACCAAACTACCACATCGCTCCTCGATGGGTTTACAACATCTCCACACTCTGGATGTGTATCGTGGTCATTTTATCAGTCTTCACCAACGGCCTCGTCTTGGTGGCCACAGCAAAGTTCAAGATACTTCGTCATCCTCTCAACTGGATCTTGGTCAACCTTGCCATCGCTGATCTTGGAGAGACAGTCTTTGCCAGCACCATCAGTGTGTGCAACCAGTTCTATGGATATTTCATTCTGGGACACCCAATGTGTGTCTTTGAAGGCTATGTTGTCTCAATTTGTGGAATTGCTGGGCTGTGGTCCCTGACCATCATCTCTTGGGAGAGATGGATAGTTGTGTGCAAACCCTTTGGAAATGTCAAGTTCGATGCC---------AAGTGGGCCACAGGTGGAATACTTTTCTCCTGGGTCTGGTCTGCAGTGTGGTGCGCTCCTCCCATCTTTGGATGGAGCAGGTACTGGCCTCATGGACTGAAAACATCCTGTGGACCTGATGTGTTCAGTGGAAATGAAGACCCTGGAATTCAGTCCTACATGATTGTCCTTGTCATTACCTGCTGCATCATTCCTCTGAGTATCATCATCTTGTGCTACCTGGCTGTGTGGTTGGCCATCCGTTCTGTTGCTATGCAGCAGCTAGATAGTGAATCAACCCAGAAGGCGGAGAGAGAAGTGTCCAGGATGGTTGTAGTCATGATCCTGGCTTTCTGTCTCTGCTGGGGACCATATGCCACTTTCGCCTGCTTTGCTGCAGCCAACCCCGGATACGCCTTTCATCCTTTGGCCGCTGCAATACCTGCATATTTAGCCAAAAGCGCCACCATCTACAACCCTGTTATCTATGTCTTCATGAACCGACAGTTTCGCACATGCATCATGCAGCTC---TTT---------------------------------------------------------------------GGCAAACAG---------GTGGATGATGATTCC------------GAAGTGTCCACA------TCGAGG---ACAGAG---------------------------------------------------GTCTCATCTGTTGCACCTGAATAA------------------------------------------------------------------------------------

>KX768597_Lnigrofasciata_LWS_2

ATGTCAGATGATTGGAGAAAACAGCCGTTTGCTGCCAGGTGGCATAATGAGGATACAACAAGG---GGCTCTGCATTCACATACACAAACAGCAATCAAACAAGA---------------GATCCCTTTGAA------GGACCAAACTACCACATTGCTCCTCGATGGGTTTATGATGTTGCAACGGTCTGGATGTGTATCGTGGTCGTTTTATCAGTCTTCACCAATGGCCTCGTCTTGGTGGCCACAGCAAAGTTCAAGAAACTTCGTCATCCTCTCAACTGGATCTTGGTCAACCTTGCCATTGCTGATCTTGGAGAGACAGTCTTTGCCAGCACTATCAGTGTGTGCAACCAGTTCTTTGGATATTTCATTCTGGGACACCCAATGTGTGTCTTTGAAGGCTATGTTGTCTCAATTTGTGGAATTGCTGGGCTTTGGTCCCTGACCATCATCTCTTGGGACAGATGGATAGTTGTGTGCAAACCTTTTGGAAATGTCAAGTTCGATGCC---------AAGTGGGCTACAGGTGGAATACTTTTCTCTTGGGTCTGGCCTGCAGTGTGGTGCGCTCCTCCCATCTTTGGATGGAGCAGGTACTGGCCTCATGGACTGAAAACGTCCTGCGGACCTGATGTGTTCAGTGGAAATGAAGACCCTGGAGTCCAGTCCTACATGATTGTGCTCATGATTACATGCTGCATCATTCCTCTGGCTATCATCATCTTGTGCTACCTGGCTGTGTGGTTGGCCATCCGTGCTATTGCTATGCAGCAGCTAGATAGTGAATCAACCCAGAAGGCTGAGAGAGAAGTGTCCAGGATGGTTGTAGTCATGATCCTGGCTTTCTGTCTCTGCTGGGGACCATATGCCACTTTCGCCTGCTTTGCCGCTGCCAACCCCGGATACGCCTTCCATCCTTTGGTCGCTGCCATGCCTGCATATTTAGCCAAAAGCGCCCCCATCTACAACCCTGTTATCTATGTCTTCATGAACCGACAGTTTCGCACATGCATCATGAAACTC---TTT---------------------------------------------------------------------GGCAAACAG---------ATGGATGATGATTCT------------GAAGTTTCCACA------TCGAAG---ACAGAG---------------------------------------------------GTCTCGTCTGTTGCACCTGAATAA------------------------------------------------------------------------------------

>KX768598_Pcaymanensis_LWS_2

ATGTCAGATGATTGGAGAAAACAGCCGTTTGCTGCCAGGTGGCACAATGAGGATACAACAAGG---GGCTCTGCATTCACATACACAAACAGCAATCAAACAAGA---------------GATCCCTTTGAA------GGACCAAACTATCACATTGCTCCTCGATGGGTTTATGATGTTGCAACGGTCTGGATGTGTATCGTGGTTGTTTTATCAGTCTTCACCAATGGCCTTGTCTTGGTGGCCACAGCAAAGTTCAAGAAACTTCGTCATCCTCTCAACTGGATCTTAGTCAACCTTGCCATTGCTGATCTTGGAGAGACAGTCTTTGCCAGCACTATCAGTGTGTGCAACCAGTTCTTTGGATATTTCATTCTGGGACACCCAATGTGTGTCTTTGAAGGCTATGTTGTCTCAATTTGTGGAATTGCTGGGCTTTGGTCCCTGACCATCATCTCTTGGGAGAGATGGATAGTTGTGTGCAAACCTTTTGGAAATGTCAAGTTCGATGCC---------AAGTGGGCCACGGGTGGAATAGTTTTCTCCTGGGTCTGGCCTGCAGTGTGGTGCGCTCCTCCCATCTTTGGATGGAGCAGGTACTGGCCTCATGGACTGAAAACGTCCTGCGGACCTGATGTGTTCAGTGGAAATGAAGACCCTGGAGTCCAGTCCTACATGATTGTGCTCATGATTACATGCTGCATCATTCCTCTGGCTATCATCATCTTGTGCTACCTGGCTGTGTGGTTGGCCATCCGTGCTATTGCTATGCAGCAGCTAGATAGTGAATCAACCCAGAAGGCTGAGAGAGAAGTGTCCAGGATGGTTGTAGTCATGATCCTGGCTTTCTGTCTCTGCTGGGGACCATATGCCACTTTCGCCTGCTTTGCCGCTGCCATCCCCGGATACGCCTTCCATCCTTTGGCCGCTGCCGTGCCTGCATATTTAGCCAAAAGCGCCACCATCTACAACCCTGTTATCTATGTCTTCATGAACCGACAGTTTCGCACATGCATCATGAAACTC---TTT---------------------------------------------------------------------GGCAAACAG---------ATGGATGATGATTCT------------GAAGTTTCCACA------TCGAAG---ACAGAG---------------------------------------------------GTCTCGTCTGTTGCACCTGAATAA------------------------------------------------------------------------------------

>KX768589_Pvelifera_LWS_2

ATGTCAGATGATTGGAGAAAACAGCCGTTTGCTGCCAGGTGGCACAATGAGGATACAACAAGG---GGCTCTGCATTCACATACACAAACAGCAATCAAACAAGA---------------GATCCTTTTGAA------GGACCAAACTACCACATCGCTCCTCGATGGGTTTATGATGTCGCAACGGTCTGGATGTGTATTGTGGTCGTTTTATCAGTCTTCACCAACGGCCTCGTCTTGGTGGCCACAGCAAAGTTCAAGAAACTTCGTCATCCTCTCAATTGGATCTTGGTCAACCTTGCCATCGCTGATCTTGGAGAGACGGTCTTTGCCAGCACCATCAGTGTGTGCAACCAGTTCTTTGGATATTTCATTCTGGGACACCCAATGTGTGTCTTTGAAGGCTATGTTGTCTCAATTTGTGGAATTGCTGGGCTTTGGTCCCTGACCATCATCTCTTGGGAGAGATGGATAGTTGTGTGCAAACCCTTTGGAAATGTCAAGTTCGATGCC---------AAGTGGGCCACAGGTGGAATAGTTTTCTCCTGGGTCTGGCCTGCAGTGTGGTGCGCTCCTCCCATCTTTGGATGGAGCAGGTACTGGCCTCATGGACTGAAAACGTCCTGCGGACCTGATGTGTTCAGTGGAAGCGATGACCCAGGGGTCCTGTCCTACATGATTGTCCTCATGATTACATGCTGCATCATTCCTCTGGCTATCATCATCTTGTGCTACTTGGCTGTGTGGTTGGCCATCCGTGCTGTTGCTATGCAGCAGCTAGATAGTGAATCAACCCAGAAGGCTGAGAGAGAAGTGTGCAGGATGGTTGTAGTCATGATCCTGGCTTTCTGTCTCTGCTGGGGACCATATGCCACTTTTGCCTGCTTTGCCGCAGCCAACCCCGGATACGCCTTCCATCCTTTGGCCGCTGCCATGCCTGCATACTTTGCCAAAAGCGCCACCATCTACAACCCTGTTATCTATGTCTTCATGAACCGACAGTTTCGCACATGCATCATGAAACTC---TTT---------------------------------------------------------------------GGCAAACAG---------ATGGATGATGATTCC------------GAAGTGTCCACA------TCGAAG---ACAGAG---------------------------------------------------GTCTCGTCTGTTGCACCTGAATAA------------------------------------------------------------------------------------

>Danio_rerio_LWS1

ATGGCAGAGCATTGGGGAGATGCAATTTATGCAGCCCGGCGAAAGGGAGATGAAACCACAAGG---GAAGCAATGTTCACATATACCAACAGTAATAACACCAAG---------------GATCCCTTTGAG------GGTCCCAATTACCACATTGCCCCTCGATGGGTGTACAATGTTGCAACAGTCTGGATGTTCTTTGTGGTTGTCGCCTCAACCTTCACCAATGGCCTGGTACTGGTGGCCACGGCCAAATTCAAGAAGCTCCGTCACCCTCTCAACTGGATCCTGGTCAACCTTGCTATAGCTGATCTGGGAGAGACTCTGTTTGCCAGCACAATCAGTGTCATTAACCAATTTTTCGGCTACTTTATCCTAGGACATCCCATGTGTATTTTTGAAGGCTACACTGTGTCAGTATGTGGTATTGCTGCACTGTGGTCGTTGACTGTCATCTCTTGGGAAAGATGGGTGGTTGTCTGTAAACCATTTGGAAATGTCAAGTTTGATGCT---------AAATGGGCATCTGCTGGCATTATCTTCTCCTGGGTTTGGGCTGCTGCTTGGTGTGCACCTCCCATCTTTGGCTGGAGCAGATACTGGCCTCATGGTCTGAAGACCTCCTGTGGCCCTGATGTCTTCAGTGGAAGCGAGGACCCTGGAGTTCAGTCCTACATGGTGGTGCTCATGATCACCTGCTGTATCATCCCTCTAGCTATTATCATTCTCTGCTACATTGCTGTGTACCTGGCCATCCATGCTGTCGCCCAGCAGCAGAAGGATTCTGAGTCCACACAGAAGGCCGAGAAGGAAGTGTCCAGAATGGTGGTTGTCATGATCTTCGCCTACTGTTTCTGCTGGGGTCCTTACACGTTCTTCGCCTGCTTTGCAGCTGCAAACCCAGGCTATGCCTTCCACCCACTGGCAGCAGCCATGCCTGCCTACTTTGCCAAGAGCGCCACCATCTACAACCCCGTCATTTATGTCTTCATGAACCGACAGTTCCGCGTATGCATCATGCAGCTC---TTT---------------------------------------------------------------------GGAAAGAAG---------GTGGATGATGGCTCT------------GAGGTGTCCACA------TCCAAA---ACAGAA---------------------------------------------------GTGTCTTCTGTGGCTCCTGCATAA------------------------------------------------------------------------------------

>Danio_rerio_LWS2

ATGGCAGAG---TGGGCCAATGGGGCATTTGCCGCGAGACGGCGAGGGGACGAAACAACAAGG---GACAACGCTTTCTCATATACCAACAGCAATAACACAAGG---------------GATCCCTTTGAG------GGTCCCAATTACCACATTGCCCCTCGATGGGTGTACAATGTTGCAACAGTCTGGATGTTCTTTGTGGTTGTCGCCTCAACCTTCACCAATGGCCTGGTACTGGTGGCCACGGCCAAATTCAAGAAGCTCCGTCACCCTCTCAACTGGATCTTGGTCAACCTTGCTATAGCTGATCTGGGAGAGACTCTGTTTTCCAGCACAATCAGCGTCATCAATCAGGTTTTCGGCTACTTTATACTCGGACATCCCATGTGTATTTTTGAAGGCTACACTGTGTCAGTATGTGGTATTGCTGGACTGTGGTCGTTGACTGTCATCTCTTGGGAAAGATGGGTGGTTGTCTGTAAACCATTTGGAAATGTCAAGTTTGATGGT---------AAATGGGCATCTGCTGGCATTATCTTCTCCTGGGTTTGGGCTGCTGTTTGGTGTGCACCTCCCATCTTTGGCTGGAGCAGGTATTGGCCTCATGGTCTGAAGACCTCCTGTGGACCTGATGTGTTTGGAGGAAACGAGGACCCCGGAGTCCAGTCCTACATGCTGGTCCTAATGATCACCTGTTGCATCCTTCCTCTTGCTATCATCATTCTCTGCTACATTGCTGTGTTCCTGGCCATCCATGCTGTTGCCCAGCAGCAGAAGGATTCTGAGTCCACACAGAAGGCCGAGAAGGAAGTGTCCAGAATGGTGGTTGTCATGGTCCTTGCTTTCTGCCTTTGTTGGGGTCCGTACACGGCCTTTGCCTGCTTTGCTGCTGCAAACCCAGGCTATGCCTTCCACCCACTGGCAGCAGCCATGCCTGCCTACTTTGCCAAGAGCGCCACCATCTACAACCCCATCATTTATGTCTTCATGAACCGACAGTTCCGCGTATGCATCATGCAGCTC---TTT---------------------------------------------------------------------GGAAAGAAG---------GTGGATGATGGCTCT------------GAGGTGTCCACA------TCCAAA---ACAGAA---------------------------------------------------GTGTCTTCTGTGGCTCCTGCATAA------------------------------------------------------------------------------------
