# Supplementary material for: Phylogenetic analysis and ontogenetic changes in the cone opsins of the western mosquitofish (Gambusia affinis)
Source: PLoS One. 2020 Oct 13;15(10):e0240313. doi: 10.1371/journal.pone.0240313 (PMC7553354; doi:10.1371/journal.pone.0240313)
Supplement: S2 Appendix — (DOCX) [file pone.0240313.s004.docx]

>LWS3_P_minor

-----------------------------------------------------------GGTGAAGGCACCCAGCAAA-CGCAGAAGGTTGTAAGCTAGAGGAAGGTGTGATAGCCGAGAAAAGCTCTGCAGAGATCAGC

>LWS3_H_formosa

---------------------------------------------------------------------CCCAGCAAA-CGCAGAAGGTTATAAGCCAGAGGAAAGTGAGACAGCTGAGAGAAGATCTCCTGAGATCAGA

>LWS3_P_picta

----------------------------GATAA-GCGTTGGTATATAAGGAGAAGTGTGAGATGAAGCACCCAGCAAA-CACAGAAGGTTATCAGCCAGAGGAAGGTGTGACAGCCAAGAGAAGCTCTCCAGAGATCAGG

>LWS3_P_parae

----------------------------------------------------AAGTGTGAGATGAAGCACCCAGCAAA-CGCAGAAGGTTATCAGCCAGAGGAAGGTGTGACAGCCGAGAGAAGCTCTCCAGAGATCAGG

>LWS3_P_bifurca

-----------------------------------------TATATAAGGAGAAGTGTGAGATGAAGCACCCAGCAAA-CGCAGAAGGTTATCAGCCAGAGGAAGGTGTGACAGCCGAGAGAAGCTCTCCAGTGATCAGG

>LWS3_X_helleri

GATTTTACAGTAATCCCATCAGAGGTCAGATAA-GCGTTGGTATATAAGGAGAAGTGTGAGGTGAAGCACCCAGCAAA-CGCAGAAGGTTATAAGCCAGAGGAAGGTTTGACAGCCGAGAGAAGATCTCAAGAGATCAGG

>LWS3_G_affinis

GATTTTAAAGTAATCCCATCAGAGATCAGATAA-GCGGTGGTATATAAAGAGAAGTGTGAGGTGAAGCACCCAGGGAA-CACAGAAGGTTATAAGCCAGAGGAAGGTGTGACAGCCGAGAGAAGATCTCCAGAGATCAGG

>LWS3_P_wingei

GATTTTAAAGTAATCCCATCAGCGATCAGATAA-GCGTTGGTATATAAGGAGAAGTGTGTGGTGAAGCACCCAGCAAA-CGCAGAAGGTTGTAAGCCAGAGGAAGGTGTGACAGCTGAGAGAAGCTCTCCAGAGATCAGG

>LWS3_P_petenensis

---------------------------------------------------------------------CCCAGCAAA-CGCAGAAGGTTATAAGCCAGAGGAAGGTGTGACAGCTGAGAGAAGCTCTCAAGAGATCAGG

>LWS3_P_vittata

-------------------------------------------------------------------------------CGCAGAAGGTTATAAGCCAGAGGAAGGTGTGACAGCTGAAAGAAGCTCTCCAGAGATCAGG

>LWS3_P_caymanensis

------------------------------------------------------------GGTGAAGCACCCAGCAAA-CGCAGAAGGTTATAAGCCAGAGGAAGGTGTGACAGCTGAAAGAAGCTCTCCAGAGATCAGG

>LWS3_P_nigrofasciata

-------------------------------------------------------------------------------CGCAGAAGGTTATAAGCCAGAGGAAGGTGTGACAGCTGAAAGAAGCTCTCCAGAGATCAGG

>LWS3_P_velifera

----------------------------GATAA-GCGTTGGTATATAAGGAGAAGTGTGAGGTGAAGCACCCAGCAAA-CGCAGAAGGTTATAAGCCAGAGGAAGGTGTGACAGCTGAGAGAAGCTCTCCAGAGATCAGG

>LWS3_P_latipinna

-----------------------------------------------------------------------------------GAAGGTTATAAGCCAGAGGAAGGTGTGACAGCTGAGAGAAGCTCTCCAGAGATCAGG

>LWS3_P_mexicana

---------------------------------------------------------------------------------CAGAAGGTTATAAGCCAGAGGAAGGTGTGACAGCTGAGAGAAGCTCTCCAGAGATCAGG

>LWS3_P_reticulata

----------------------------------------GTATATAAGGAGAAGTGTGAGGTGAAGCACCCAGCAAA-CGCAGAAGGTTATAAGCCAGAGGAAGGTGTGACAGCTGAGAGAAGCTCTCCAGAGATCAGG

>LWS1_G_affinis

-----------------------------AGGGTGTCTGACTTGCTCCACATGTCAGCAGAGGCAACAGTCACGATCACCTCGGGAGGTTACAAGTGAGAGGAAGGTCTGAGAGCTGAGAAACCTTCTTCCCAGACCAGG

>LWS1_P_picta

--------------------------------------------------------------TGTGAAGTGCAGATCACCTAGGGAGGTTACAAGTGAGAGGACG-------AGCTGAGAAACCTTCTTTAAAGATTAGA

>LWS1_P_parae

-----------------------------------------------------------------------CAGATCACCTAGGGAGGTTACAAGTGAGAGGACG-------AGCTGAGAAACCTTCTTTAAAGATTAGG

>LWS1_P_bifurca

-----------------------------------------------------------------------------------GGAGGTTACAAGTGAGAGGACG-------AGCTGAGAAACCTTCTTTAAAGATTAGG

>LWS1_P_reticulata

--------------------------------------------------------------TGTGAAGTGCAGATCACCTAGGGAGGTTACAAGTGAGAGAAAG-------AGCTGAGAAACCTTCTTTCCAGATCAGG

>LWS1_P_wingei

GATTGTGAAGTAATCCGCTCAGAGGTCACCGAG-GCGTCGGTATAAAAGCTAAAGTGTGAAGTG-------CAGATCACCTAGGGAGGTTACAAGTGAGAGGAAG-------AGCTGAGAAACCTTCTTTCCAGATCAGG

>LWS1_X_helleri

GATTGTGAAGTAATCCGCTCAGAGGTCACTGAATGCAACGGTATAAAAGCTAAAGTGTGATGTGAAGTGC--AGATCACCTCGGGAGGTTACAAGTGAGAGGAAGGTCTGAGAGCTGAGAAACCTTCTTCCCAGATCAGG

>LWS1_H_formosa

--------------------------------------------------------------TGTGAAGTGCAGATCACCTAGGGAGGTTACAAGTTAGAGGAAGGTCTGAGAGCTGAGAAACCTTCTTCCCAGATCAGA

>LWS1_P_nigrofasciata

---------------------------------------------------------------------------------------------------------------------------TTTATTTCCAGATCAGG

>LWS1_P_minor

--------------------------------------------------------------TGTGAAGTGCAGATCACCTAGCGAAGTTACAAGTGAGAGGAAG-------AACTGAGGAACATTATTTCCAGATCAGG

>LWS1_P_vittata

-------------------------------------------------------------------------------CTAGGGAGGTTACAAGTGAGAGGAAG-------AACTGAGGAACATTATTTCCAGATCAGG

>LWS1_P_caymanensis

--------------------------------------------------------------TGTGAAGTGCAGATCACCTAGGGAGGTTACAAGTGAGAGGAAG-------AACTGAGGAACATTATTTCCAGATCAGG

>LWS1_P_mexicana

-------------------------------------------------------------TTGTGAAGTGCAGATCACCTAGGGAGGTTACAAGTGAGAAGAAG-------AACTGAGGAACATTATTTCTAGATCAGG

>LWS1_P_latipinna

-------------------------------------------------------------TTGTGAAGTGCAGATCACCTAGGGAGGTTACAAGTGAGAAGAAG-------AACTGAGGAACATTATTTCCAGATCAGG

>LWS1_P_velifera

----------------------------------------------------------------------------CACCTAGGGAGGTTACAAGTGAGAAGAAG-------AACTGAGGAACATTATTTCCAGATCAGG

>LWS1_P_petenensis

--------------------------------------------------------------------------ATCACCTAGGGAGGTTACAAGTGAGAAGAAG-------AACTGAGGAACATTATTTCCAGATCAGG

>LWSR_P_picta

-------------------------------------------------------------------------------TCCACAAGGT-ACAAGCCAGAGGAAGATCAAGCAGCTCAGATTGTCTTTCCA-AGATCAGG

>LWSR_P_latipinna

------------------------------------------------------------------------------------------------------AAGATCAAGCAGCTCAGATTGTCTTTCCA-AGATCAGG

>LWSR_P_velifera

--------------------------------------------------------------------------------CCACAAGGTTACAAGCCAGAGGAAGATCAAGCAGCTCAGATTGTCTTTCCA-AGATCAGG

>LWSR_P_petenensis

---------------------------------------------------------------------------------CACAAGGTTACAAGCCAGAGGAAGATCAAGCAGCTCAGATTGTCTTTCCA-AGATCAGG

>LWSR_G_affinis

GGTCTAAAAGTAATTCCATCAGAGTTCAGTGAA-CTGCGGGTGTATAAGCTAAAGTATGAGGATAAGCACCGCACAAA-CCCACAAGGTTACAAGCCAGAGGAAGATCAAGCAGCTCAGATCGTCTTTCCA-AGATCAAG

>LWSR_P_nigrofasciata

--------------------------------------------------------------------------------CCACAAGGTTACAAGCCAGAGGAAGATCAAGCAGCTCAGATCGTCTTTCTA-AGATCAGG

>LWSR_P_wingei

GGTTTTAGAGTAATCCCATCAGAGGTCAGTGAA-CTGCAGGTGTATAAGCTAAAGTATGAGGATAAGCACCGCACAAA-CCCACAAGGTTACAAGCCAGAGGAAGATCAAGCAGCTCAGATCGTCTTTCCA-AGATCAGG

>LWSR_X_helleri

GGTCTTAAAGTAATCCCATCAGAGTTCAGTGAA-CTGCGGGTGTATAAGCTAAAGTATGAGGATAAGCACCGCACAAA-CCCACAAGGTTACAAGCCAGAGGAAGATCAAGCAGCTCAGATCGTCTTTCCA-AGATCAGG

>LWSR_P_mexicana

--------------------------------------------------------------------------------CCACAAGGTTACAAGCCAGAGGAAGATCAAGCAGCTCAGATCGTCTTTCCA-AGATCAGG

>LWSR_P_reticulata

-----------------------------------------------------------------------------------------------------GAAGATCAAGCAGCTCAGATCGTCTTTCCA-AGATCAGG

>LWSR_P_vittata

--------------------------------------------------------------------------------CCACAAGGTTACAAGCCAGAGGAAGATCAAGCAGCTCAGATCGTCTTTCCA-AGATCAGG

>LWSR_P_caymanensis

--------------------------------------------------------------------------------CCACAAGGTTACAAGCCAGAGGAAGATCAAGCAGCTCAGATCGTCTTTCCA-AGATCAGG

>LWSR_P_parae

--------------------------------------------------------------------------------------------AAGCCAGAGGAAGATCAAGCAGCTCAGATCGTCTTTCCA-AGATCAGG

>LWSR_P_bifurca

------------------------------------------------------------------------------------AAGGTTACAAGCCAGAGGAAGATCAAGCAGCTCAGATCGTCTTTCCA-AGATCAGG

>LWSR_P_minor

--------------------------------------------------------------------------------CCACAAGGTTACAAGCCAGAGGAAGATCAAGCAGCTCAGATCGTCTTTCCA-AGATCAGG

>LWSR_H_formosa

--------------------------------------------------------------------------------CCACAAGGTTACAAGCCAGAGGAAGATCAAGCAGCTCAGATCGTCTTTCCA-AGATCAGG

>LWS2_H_formosa

--------------------------------------------------------------------------------TCTCAAGGTTATAAAC----------------AACCAAGTACCGTTTTCTA-AGAACAA-

>LWS2_X_helleri

GGTTTTAAAGTAATCCCATCAGAGTTCTGTGAA-GCGTAGGTATATAAGCTGACGTCTGATGTGAGGCACCCAGCAAAACTCTCAAGGTTATAAAC-------------GACAACTAAGTACCTCTTTCGA-AGAACAAG

>LWS2_P_minor

--------------------------------------------------------------------CCCCAGCAAAACTCTCAAGGTTATAAAC-------------AACAACTAAGTATCTCTATC---AGACCAGG

>LWS2_P_mexicana

---------------------------------------------------------------------CCCAGCAAAACTCTCAAGGTTATAAAC-------------GACAACTAAGTATCTCTATCAA-AGACCAGG

>LWS2_P_velifera

---------------------------------------------------------------------CCCAGCAAAACTCTCAAGGTTATAAAC-------------GACAACTAAGTATCTCTATCAA-AGACCAGG

>LWS2_P_petenensis

---------------------------------------------------------------------CCCAGCAAAACTCTCAAGGTTATAAAC-------------GACAACTAAGTATCTCTATCAA-AGACCAGG

>LWS2_P_latipinna

----------------------------------------------------------------------------------TCAAGGTTATAAAC-------------GACAACTAAGTATCTCTATCAA-AGACCAGG

>LWS2_P_nigrofasciata

-------------------------------------------------------------------------------CTCTCAAGGTTATAAAC-------------GACAACTAAGTATCTCTATCAA-AGACCAGG

>LWS2_P_vittata

-------------------------------------------------------------------------------CTCTCAAGGTTATAAAC-------------GACAACTAAGTATCTCGATCAA-AGACCAGG

>LWS2_P_caymanensis

---------------------------------------------------------------------CCCAGCAAAACTCTCAAGGTTATAAAC-------------GACAACTAAGTATCTCGATCAA-AGACCAGG

>LWS2_P_picta

---------------------------------------------------------------------CCCAGCAAAACTCTCAAGGTTATAAAC-------------GGCAACTAAGTACCTCTATCAA-AGAATAGG

>LWS2_P_bifurca

------------------------------------------------------------------------------------AAGGTTATAAAC-------------GGCAACTAAGTACCTCTATCAA-AGAATAGG

>LWS2_P_parae

-------------------------------------------------------------------------------CTCTCAAGGTTATAAAC-------------GACAACTAAGTACCTCTATCAA-AGAATAGG

>LWS2_P_reticulata

---------------------------------------------------------------------------AAAACTCTCAAGGTTATAAAC-------------GACAACTAAGTACCTCTATCAA-AGAATAGG

>LWS2_P_wingei

------AAACTAATCCCATCAGAGTTCTGTGAA---------GCGAAGGCTGAAGTCTGATGTGAGGCAACCAGCAAAACTCTCAAGGTTATAAAC-------------GACAACTAAGTACCTCTATCAA-AGAATAGG

>JF262088_Oreochromis_niloticus

GATTGTGACCTAATCCAATCAAAGGTTTGTGAA-GCATAGGTATAAAAGCAAAAGTCAGATCTGTAGCACCAAGCAG--CCAAGGAGGTAGCAAGTGACAAGAAAGGCTAACAGCTCAGGACCTCCTTCTA-AGAAAAGA
